# High Plasma Vitamin B12 and Cancer in Human Studies: A Scoping Review to Judge Causality and Alternative Explanations

**DOI:** 10.3390/nu14214476

**Published:** 2022-10-25

**Authors:** Rima Obeid

**Affiliations:** Department of Clinical Chemistry and Laboratory Medicine, Saarland University Hospital, D-66424 Homburg, Germany; rima.obeid@uks.eu; Tel.: +49-6841-1630711

**Keywords:** cancer, cancer mortality, cancer risk, carcinogenic, causal inference, haptocorrin, intake, vitamin B12

## Abstract

Patients with cancer have been reported to show elevated plasma concentrations of vitamin B12, thus causing uncertainties regarding safety of vitamin B12. We conducted a systematic literature search and a scoping review of human studies published in PubMed between January 2005 and March 2022, to investigate the association between vitamin B12 (concentrations of B12 biomarkers, intake, and genetic determinants) and cancer. Except for liver cancer, the association between plasma vitamin B12 concentrations and cancer was not consistent across the studies. Vitamin B12 intake from food, or food and supplements, showed even less consistent associations with cancer. There was no evidence for temporality, coherence, or a biologically meaningful dose-response relationship between plasma vitamin B12 concentrations and cancer. Genetically determined high plasma vitamin B12 was likely to be associated with cancer. Available randomized controlled trials have used a high dose of multivitamin supplements and cancer was the unplanned outcome, thus the causality of B12 in cancer cannot be judged based on these trials. Additionally, low plasma vitamin B12 concentrations were common in patients with cancer. Therefore, there is not sufficient evidence to assume that high plasma vitamin B12, high B12 intake, or treatment with pharmacological doses of vitamin B12, is causally related to cancer. Low vitamin B12 status in patients with cancer needs to be diagnosed and treated in order to prevent the hematological and neurological sequela of the deficiency.

## 1. Introduction

Vitamin B12 (cobalamin) is needed to maintain body functions. The liver and the kidney are the main vitamin B12-storing organs. Vitamin B12 deficiency can be caused by low intake from foods or by malabsorption disorders. Anemia and irreversible neurological symptoms could develop if patients are not timely treated with the vitamin.

Patients who cannot absorb vitamin B12 need a lifelong treatment. Thus, the safety of vitamin B12 (i.e., cyanocobalamin) is a key aspect. There is so far no evidence to support setting a tolerable upper intake level for vitamin B12. 

High concentrations of vitamin B12 in plasma are common and have shown associations with cancer and cancer-related mortality. However, studies have also shown either no association or even low plasma vitamin B12 to be associated with cancer, suggesting that high plasma B12 concentrations could be an epiphenomenon rather than causally related to the pathophysiology of cancer. In general, the interpretation of the overall evidence is difficult due to discrepancy in the results according to the exposure (i.e., B12 intake versus plasma levels), cancer types, and study designs. Moreover, uncertainty regarding a possible role of vitamin B12 in promoting the growth of cancer cells can delay the treatment of patients with B12 deficiency. The relationship between vitamin B12 and cancer deserves in depth evaluation.

There are two vitamin B12-binding proteins in the circulation [1]. Transcobalamin I (TCN1, also known as haptocorrin, R-factor, or R-protein) binds vitamin B12 and prevents its degradation in the acidic pH of the stomach. In the duodenum, vitamin B12 is released from TCN1 and binds to intrinsic factor that facilitates its uptake in the enterocytes. Transcobalamin II (TCN2) binds approximately 20% of vitamin B12 in the circulation to form holotranscobalamin (holoTC) that is delivered to tissues where the vitamin is stored or used to support cell metabolism. Eighty percent of plasma total vitamin B12 is bound to TCN1 (known as holohaptocorrin), a fraction of a so far unknown physiological functions. Beside low plasma levels of vitamin B12 in people with vitamin B12 deficiency, the plasma concentrations of methylmalonic acid (MMA) become elevated due to the impaired activity of the mitochondrial methylmalonyl-coenzyme A (CoA) mutase, that converts succinyl-CoA to methylmalonyl-CoA. Recent studies suggested that the dysregulation of propionate metabolism leading to the accumulation of MMA may be related to cancer cell invasiveness [2,3].

If high plasma or serum vitamin B12 would be causally related to cancer, it is expected that treatment with vitamin B12 (that raises plasma vitamin B12) would also cause cancer. Similarly, higher vitamin B12 intake from foods, including supplements, could be associated with cancer. However, the association between B12 intake and cancer could be confounded by the consumption of animal foods such as red meat that shows associations with plasma B12 and cancer in the same time. In addition, genetic factors could lead to the high expression of TCN1 in cancer tissues, which may in theory trap more vitamin B12 in plasma. Figure S1 shows the acyclic graph of the potential association between vitamin B12 and cancer.

We conducted a scoping review of human studies based on a systematic literature search in PubMed since 2005 to investigate the “direction” and the “nature” of the association between vitamin B12 and cancer. The evidence was derived from several types of exposure (plasma concentrations of B12 or its biomarkers, B12 intake from foods or supplements, and genetically determined high plasma vitamin B12). We also evaluated the fulfillment of key causality criteria, investigated gaps in knowledge, possible evidence against a causal relationship, and possible alternative explanations.

## 2. Materials and Methods

We conducted a systematic search and scoping review of studies published between January 2005 and March 2022 to investigate whether vitamin B12-related exposures (concentrations in serum or plasma, intake from diet and/or supplements, treatment with B12, and genetic determinants of B12) are associated with cancer. The study was conducted according to a priori protocol (unpublished).

The populations of interest were people with pre-cancer conditions, those recently diagnosed with cancer, those who developed cancer or cancer-related outcomes such as death or recurrent cancers. The controls are people who were free of cancer or those with cancer, but with better outcomes (i.e., survived).

The exposures of interest were concentrations of vitamin B12 biomarkers (plasma vitamin B12, MMA, and holoTC), vitamin B12 intake (from foods and/or supplements), intervention with vitamin B12 (any form, duration, or combination with nutrients or drugs), or genetic variants related to B12 metabolism. The search also included studies reporting on plasma concentrations of B12-binding proteins (TCN1 and TCN2) that could provide mechanistic explanation for the associations. All effect size measures such as odds ratio (OR) and 95% confidence intervals (95%CI), hazard ratio (HR), relative risk (RR and 95%CI), mean (standard deviation, SD), and median (and any measure of data dispersion), were eligible.

The outcome could be neoplasia (pre-cancer), cancer, cancer recurrence, cancer mortality, cancer progression, or biomarkers of cancer progression or severity. All types and locations of cancer were included. Eligible study designs were case-control, nested case control, cross-sectional studies among patients with cancer, cohort studies (longitudinal), and randomized controlled trials (RCTs). Studies based on pooled individual participant data of multiple studies were also eligible, since we did not plan to run a meta-analysis. Eligible sources of data were primary studies or secondary data from health register, hospital records, or previous RCTs. Existing systematic reviews and meta-analysis (usually on specific exposures and outcomes), individual participant meta-analysis, and mendelian randomization studies were eligible. Key studies that were published before 2005 and included in previous meta-analyses were also eligible.

The exclusion criteria were case reports, conference abstracts, animal studies, cell-culture studies, systematic reviews without meta-analysis, expert opinions, narrative reviews, multiple publications from the same population, and studies published in languages other than English. The authors of the original articles were not contacted to obtain missing information. We did not plan to extend existing meta-analyses or run novel meta-analyses. Elevated plasma concentrations of vitamin B12 due to immune complexes [4,5] or macro-transcobalamin were not a subject of the present review [6].

### Search Methodology

The systematic search in PubMed used the search strings shown in Table S1. The reference lists of published systematic reviews and meta-analyses were screened to identify additional relevant studies. An additional hand search among the subsequent citing articles was conducted in PubMed. The search did not focus on genetic studies, due to the complexity of these studies and difficult interpretation with regard to the direction and directness of the association with cancer. The search, screening, and data extraction were conducted by the author.

We extracted data on first author, PubMed identification number, publication year, country of origin of the participants, study design, cohort name/or data source, the country of origin, the date of recruitments, study primary question, the number of participants, the type of cancer, age, sex, the follow up time in longitudinal or treatment studies, plasma B12 concentrations or other B12 markers measured in patients/controls, the inclusion and exclusion criteria of the original studies, B12 analytical method, effect size (i.e., RR, OR, HR), what was considered as exposed/not exposed, reference categories, and key results. In RCTs, B12 dose, the route of administration, the duration of treatment, combination with other vitamins, the comparator, and the primary outcomes were documented. In studies on vitamin B12 intake from food and supplements, the B12 intakes, plasma B12 concentrations if measured, and methods of the measurement of both exposures (i.e., food frequency questionnaire for intake) were recorded. In studies of genetic variants, we documented the name of the variant and the genotype used as a reference group (if reported).

In all studies, we focused on the maximally adjusted effect size if both crude and adjusted models were reported. The variables that were adjusted for were documented. The results of the independent studies were tabulated and summary tables were prepared for this publication according to the type of cancer.

## 3. Results

The search yielded 238 potentially relevant articles. After first wave screening of title and abstracts, 99 candidate articles were eligible for full-text screening. An additional 42 studies were identified from the references of the included articles, articles citing those, and a hand search. The final appraisal included 118 studies (Figure S2, study flow diagram). The excluded studies (*n* = 23) and the reason for exclusion are shown in Table S2.

### 3.1. Vitamin B12 and Liver Cancer

Six studies (four case-control and two cohort studies) addressed the association between vitamin B12 (plasma concentration and/or intake) and hepatic cancer or mortality after the diagnosis of hepatic cancer [7,8,9,10,11,12]. The exposure in five of those studies was serum/plasma B12, while one study additionally investigated vitamin B12 intake [7] and one study investigated only vitamin B12 intake [12]. The reference categories of plasma B12 concentrations in the individual studies were based on the within-study data distribution and showed large variations between the studies (i.e., reference categories of plasma B12 were <699 ng/L (or 516 pmol/L); 227–265 pmol/L; <154 pmol/L; and 200–600 pmol/L). People with low vitamin B12 concentrations (<200 pmol/L) were excluded in only one study [11].

All studies were consistent in showing that higher plasma vitamin B12 concentrations were associated with hepatic cancer or with short-term mortality after diagnosing cancer, compared to when the concentrations were lower (Table 1).

Vitamin B12 intake did not differ between patients with liver cancer and the controls and the intake was also not associated with the plasma concentrations of B12 [7]. No association was found between pre-diagnostic B12 intake and all-cause mortality and hepatic cancer-specific mortality during 791 days follow up among patients recently diagnosed with liver cancer [12].

### 3.2. Critical Evaluation

The liver stores large amounts of B12. When liver cells are damaged, vitamin B12 is released into the plasma. Lin et al. found that higher plasma B12 concentrations were associated with liver cancer, while B12 intake did not differ between cases and controls [7]. These results, combined with the lack of association between pre-diagnostic B12 intake and cancer [12], strongly suggest that high plasma B12 is not explained by high vitamin B12 intake. Lin et al. also reported associations between high plasma vitamin B12 concentrations and tumor size, the degree of liver injury, and tumor progression [7], suggesting that with progression of the tumor and liver damage, vitamin B12 (of so far unknown origin) is increasingly trapped in the circulation.

Simonsen et al. found that vitamin B12 concentrations were equally elevated in patients with a variety of liver diseases, but without liver cancer [9]. Although all studies we identified showed that high plasma B12 is associated with a higher risk of liver cancer, or cancer-mortality, these results are not likely to be specific for cancer and seem instead to be specific to liver damage of any cause. Liver tissue damage is a risk factor for liver cancer and will cause high plasma B12 concentrations in the same time. Therefore, the degree of liver damage could drive the association between plasma B12 and liver cancer.

One study excluded participants with plasma B12 concentrations below 200 pmol/L [11]. Another study reported that plasma B12 concentrations below 185 pmol/L were more prevalent in patients with liver cancer (14.7%) than in the controls (8.6%) [8]. Therefore, vitamin B12 deficiency could be common among patients with liver cancer, which could be related to inability of the damaged liver to metabolize or store B12.

There was insufficient evidence to suggest a biologically meaningful threshold for the association between plasma vitamin B12 concentrations and the risk of liver cancer [10], or the risk of mortality after diagnosing liver cancer [11]. For example, Chang et al. reported significantly higher OR for liver cancer within the physiological range of serum B12 concentrations (i.e., Q3 229–324 pmol/L and Q4 > 324 pmol/L) [10].

A cohort study based on health register data showed a higher 30-days mortality after diagnosing several types of cancer (including liver cancer) when vitamin B12 concentrations measured up to 1 year before diagnosis of cancer were above 800 pmol/L [11]. Among patients with newly diagnosed cancer who had their plasma vitamin B12 measured up to 1 year before the diagnosis, 6.6% of the cases had plasma B12 concentrations above 800 pmol/L (and 93.4% lower than this cut-off value) [11]; whereas, among patients who were diagnosed more than 1 year after the measurement of their plasma B12, only 4.4% had plasma B12 above 800 pmol/L [11]. Therefore, high plasma concentrations of vitamin B12 were more common shortly (1 year) before the diagnosis of cancer, but less common when measured at earlier time points before the diagnosis of cancer. Considering the long time needed for liver cancer progression and the chronic liver damage that precede the diagnosis of cancer, it is unlikely that high plasma B12 will lead to tumor progression and death within 1 year. Irrespective of vitamin B12 concentrations, the late diagnosis of liver cancer is associated with short survival. The disease stage at which the diagnosis of liver cancer is made will depend on the health care system, screening programs, and the history of chronic liver disorders, that also increase the likelihood that the patients will be on regular health checks. Studies based on register data (i.e., [11]) are likely to be confounded by indication, as explained in Figure S3. In addition, excluding patients with B12 deficiency may introduce a selection bias. It is also likely that vitamin treatment and supplement users are under-reported in data from health registries.

**Figure 1:**
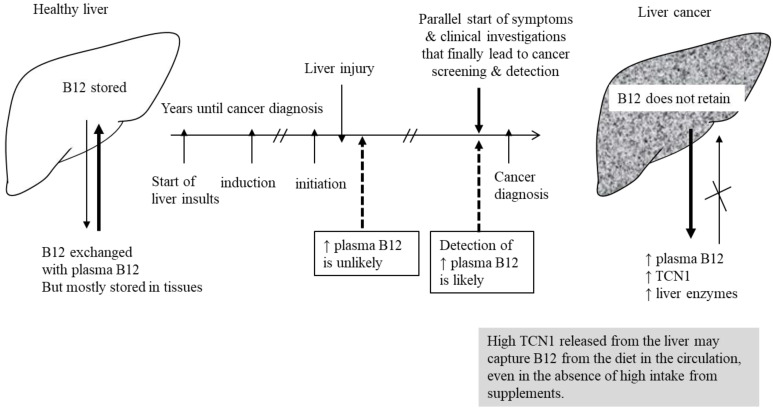
Disturbed physiology of vitamin B12 in patients with liver cancer leading to high concentrations of B12 in plasma.

Concentrations of TCN1 have been shown to be elevated in the plasma of patients with several liver disorders, including cancer [9]. In line with this result, Liu et al. found that mRNA expression of TCN1 is upregulated in colon cancer tissues compared to adjacent tissues [13]. TCN1 gene expression is upregulated in tumor cells, which could be related to cellular events that are typical to cancer, such as apoptosis and inflammation. The excessive release of TCN1 from cancer tissues could lead to capturing B12 and trapping it in the circulation, instead of storing the vitamin in the tissues. A possible explanation of high plasma vitamin B12 concentrations in liver cancer is shown in Figure 1.

### 3.3. Vitamin B12 and Other Type of Cancers

Supplemental Data File S1 shows a critical appraisal of the literature identified on the topic of vitamin B12 and cancers of the esophagus and stomach, pancreas, breast, ovarian, prostate, lung, kidney, bladder, colon, and rectum, in addition to studies on mixed types of cancer or pediatric cancers. The key results of the studies are summarized in Tables S3–S10.

### 3.4. Genetic and Mendelian Randomization Studies

We collected data from 16 studies on genetic variants affecting vitamin B12 metabolism, or vitamin B12 transport, and the risk of different cancers (Table S11). Several meta-analysis, and mendelian randomization studies, showed mixed results regarding the association between genetically determined high B12 and the risk of cancer [14,15,16,17,18,19,20]. The most common single variant reported in relation to cancer was the methionine synthase MTR A2756G (rs1805087) (chromosome 1q43), which is an A-to-G transition at base-pair 2756 and leads to a change from aspartic acid to glycine at codon 919 (D919G). The CC genotype of this variant is associated with higher serum folate concentrations [21] and lower homocysteine concentrations [22], although the functional impact of this variant on cancer is unknown. Numerous polymorphisms in genes related to vitamin B12 metabolism or transport have been studied in relation to cancer risk (i.e., rs526934 in the TCN1 gene, and cubulin haplotypes [23]). However, vitamin B12-related variates showed different associations with plasma vitamin B12 concentrations that vary between populations [24] and could show interactions with vitamin B12 intake. This heterogeneity in the definition of “genetically determined high vitamin B12” makes data interpretation difficult.

In the meta-analysis of Yu et al., there was a reduced risk of acute lymphoblastic leukemia and colon cancer in subjects carrying MTR 2756GG genotype [17]. Lu et al. found no significant association between the MTR A2756G polymorphism and breast cancer risk for the GG versus AA genotype, while in the stratified analysis, significantly decreased breast cancer risks was found for the GG/AG versus the AA genotype [16]. In a mendelian randomization study, Tsilidis et al. reported that a genetically predicted high vitamin B12 level was associated with higher OR and (95% CI) for colorectal cancer [20]. In contrast, Guo et al. reported no association between genetically predicted vitamin B12 concentration and low malignant epithelial ovarian cancers after removing one outlier study [19].

Genetic studies were heterogeneous, but generally rather supportive of an association between genetically determined high plasma vitamin B12 and cancers. However, the interpretation of this association as causal is not possible due to uncertainties of whether the genetic factors are directly related to cancer, or via interaction with intake/status of nutrients. It is also not known whether the genotype may affect TCN1 or other confounders, as discussed below. Zhong et al. suggested that the heterogeneity in the results on the association between MTR A2756G and breast cancer could be due to selection bias or confounding not accounted for in the individual studies [18].

The basic principle of mendelian randomization is that genetic variants that either alter the level of, or mirror the biological effects of, plasma vitamin B12 that itself could affect the risk of cancer should be related to cancer risk to the extent predicted by their influence on plasma B12. However, this principle assumes that there is no other direct effect of the genetic variants on the risk of cancer that goes via alternative ways (either directly affecting cancer growth or by affecting some confounder). There is no evidence to suggest that these assumptions are valid.

### 3.5. Randomized Controlled Trials, Their Secondary Analyses and Meta-Analyses

We did not identify any trial on vitamin B12 monotherapy or supplementation, and cancer. To the best of our knowledge, there are no such trials at present (Table S12). We identified five secondary publications from four independent RCTs [25,26,27,28,29], in addition to one meta-analysis of RCTs [30]. In all studies, vitamin B12 was administered together with high doses of folic acid and vitamin B6. Two publications found no increase in the risk of cancer [26,28]; two publications reported an increased risk of cancer or cancer mortality [25,29]; and one study found a higher risk of cancer in a subgroup of patients with diabetes [27]. The meta-analysis of RCTs does not support an association between treatment with the B-vitamins and the risk of cancer [30].

## 4. Discussion

### 4.1. Does High Plasma B12 Concentrations or High B12 Intake Cause Cancer

From the studies discussed above and in Supplemental Data File S1 and Tables S3–S11, we evaluated a possible causal link between elevated plasma vitamin B12 and cancer, according to the Bradford-Hill criteria of causality (Table 2).

### 4.2. Alternative Mechanisms That Could Be in Play

It is in theory possible that high plasma vitamin B12 can be caused by subclinical and manifested cancers; but why is this observation not consistent across the studies?

TCN1 is a cobalamin-binding glycoprotein found in blood, salivary and mucosal secretions. Tissues enriched with TCN1 are salivary gland, urothelial cells, basal respiratory cells, pancreatic cells, and immune cells [43,44]. TCN1 is not responsible for delivering B12 into the tissues (possibly with exception of the liver). This protein is overexpressed in cancer tissues [45,46]. Sheppard et al. suggested that tumor tissues showing high unsaturated binding capacity could be the source of the increased binding protein in the serum [46]. Moreover, with exception to liver cancer, tumors of the brain, the heart, and the lung, they were associated with higher vitamin B12 binding capacity compared to the normal tissues [46].

TCN1 has been suggested as a potential marker for granulocyte differentiation [43] and an unfavorable prognostic marker in renal and lung cancers [47]. In line with this prognostic role, Wang et al. have shown that TCN1 protein and mRNA were lower in patients sensitive to neoadjuvant chemotherapy versus those who were not sensitive for this treatment [48]. Burger et al. reported 10 times more R-type protein in liver tumor tissues than in normal liver tissue samples from the same patient [49]. The authors speculated about increased synthesis and secretion of this R-type protein by the tumor itself, to explain the elevated plasma concentrations of this protein in the patient [49].

Neutrophils are determinately important for cell repair and defense against insults including inflammation and cancer [50,51]. These immune cells are typically recruited to the affected cellular site and are capable of eliminating pathogens by multiple mechanisms [52]. These cells originate from hematopoietic cords in the bone marrow and have a short lifespan after their release into the circulation. Mature neutrophils are present in large reservoir in the liver, the lung, and the spleen. One hypothesis is that the tissue reservoir of the neutrophils (expressing TCN1) can surround the tumor and attack it, thus explaining the high staining of TCN1 or its gene expression in tumor cells, compared to adjacent cells [13,49,53], and the association between high TCN1 expression in the tumor and low patient survival [53].

**Figure 2:**
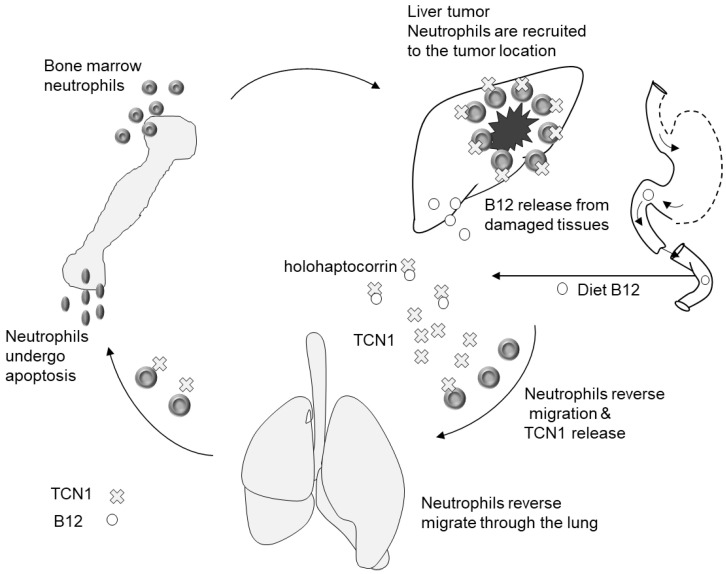
Possible pathomechanisms explaining elevated plasma vitamin B12 in patients with some cancers. Haptocorrin (TCN1) is overexpressed in cancer tissues and this expression is related to the stage of tumor, apoptosis, and inflammation processes. The short-living neutrophils originate from the bone marrow. The neutrophils express high level of TCN1 and are engaged in the body defense against cancer tumor. Neutrophils-originated-TCN1 may capture B12 and explain its retention in plasma.

TCN1 may have a role in cancer progression or in the response of the body against cancer. This suggestion is supported by: (1) the finding that TCN1 mRNA is overexpressed in cancer tissues compared to adjacent tissues [13]; (2) TCN1 expression is associated with apoptosis and inflammation (i.e., cellular events that are typical to cancer) [53]; and (3) high tissue TCN1 is associated with the presence of invasive tumors, higher tumor markers, metastasis to regional lymph [53], and low response to neoadjuvant chemotherapy [48]. Larger and more aggressive tumors appear to produce more TCN1 that could be, in theory, released to the blood and become available to capture vitamin B12, and cause high B12 in people with cancer (Figure 2).

An open question is whether vitamin B12 binders (TCN1 and TCN2) are upregulated to the same degree in patients with cancer. Carmel and Hollander found very high concentrations of TCN2 in patients with chronic lymphocytic leukemia, multiple myeloma, and other types of proliferative cancers [54]. The authors suggested that TCN2 elevation may resemble an acute phase response to cancer infection and inflammation. However, there were also uncertainties about the origin of TCN2, and the potential effect of genetic factors since TCN2 was not uniquely elevated in all of the participants [54]. The authors also suggested the possibility of impaired TCN2 elimination from the circulation in patients with cancer [54]. The kinetics and predictors of vitamin B12 binding proteins in patients with cancer deserve in depth investigation.

Three studies reported parallel measurements of plasma total B12 and holoTC concentrations in patients with esophageal squamous cell carcinoma, prostate, and lung cancers [38,55,56]. We calculated the ratio of mean (or median) plasma B12/to holoTC concentrations (both in pmol/L) in the patients and the controls from each study. The aim was to judge whether plasma B12 concentrations (mostly representing vitamin B12 bound to TCN1) were proportionally higher than B12 levels bound to transcobalamin in the patients, versus the controls. This ratio ranged between 3.0 and 5.4 in the different studies, but did not appear to differ between the patients and the corresponding controls from the same study. In a study on lung cancer, the plasma B12/holoTC ratio was 3.6 in patients with adeno–carcinoma, 2.7 in patients with squamous cell carcinoma, and 3.2 in patients with large cell carcinoma [56]. A similar plasma B12/holoTC ratio in patients with cancer and the controls suggests that the proportion of B12 bound to TCN1 to that bound to transcobalamin is unchanged in patients with cancer, thus both B12-binding proteins may be upregulated to the same extent.

The successful treatment of cancer has been shown to be associated with lowering vitamin B12 concentrations in studies with repeated measurements of plasma B12 concentrations [57]; whereas, curative cancer treatment was not associated with a reduction of plasma B12 concentrations [57], suggesting that the presence of cancer is the cause of B12 elevation in plasma and not vice versa.

The association between high plasma B12 and cancer, or cancer mortality, was present in a series of cohort studies based on Danish health register data [11], whereas results of case-control studies were inconclusive. This difference could be due to the design of the cohort studies and possible bias, and unknown confounding, as explained in Figure S3.

The proportion of intracellular versus extracellular vitamin B12 in patients with cancer is not known. Intracellular B12 could be low even when B12 is accumulating in the circulation. Vitamin B12 and its analogues have been used as radiopharmaceuticals to visualize tumors depending on the affinity of tumor tissues to the labelled-B12 [58]. An accumulation of labelled-vitamin B12 in tumor tissues suggests that these tissues were depleted of the vitamin prior to the visualization. In line with this suggestion, higher tissue-accumulation of radiolabelled adenosylcobalamin was found among patients with high baseline plasma vitamin B12, who (according to the present knowledge) may have more advanced cancer [59]. Moreover, patients with low-grade malignancies showed less accumulation of the labelled B12 compound and did not show good imagining of the tumor [59]. High plasma B12 concentrations do not necessarily mirror vitamin B12 availability to tissues.

### 4.3. Additional Arguments against a Causal Role of B12 in the Etiology of Cancer and Possible Non-Causal Explanations

We found no clear pattern of the association according to adenocarcinomas (affecting an organ or a gland) and squamous cell carcinoma (affecting the squamous epithelium), suggesting that vitamin B12 has no functional role in tumor pathophysiology. Additionally, low plasma/serum B12 concentrations were associated with cancer or were found to be more common in patients with cancer than in the controls [8,37,60] (i.e., cervical cancer), suggesting that the availability of vitamin B12 is not determinately important for cancer progression.

The lack of coherent results from studies on B12 intake and plasma concentrations argues against the causality of B12 in cancer. Studies on vitamin B12 intake from foods, or foods and supplements, were less consistent than those on blood concentrations. Additionally, low B12 intake has also been shown to be associated with cancer [61,62]. Studies collecting nutritional intake data in the past years are subject to recall bias and are not a reliable source of evidence on causality. Thus, vitamin B12 intake has not been reliably shown to be associated with cancer. High plasma vitamin B12 concentrations (despite comparable intake) in patients with some cancers, compared to the controls, suggest changes in B12 homeostasis that lead to capturing and trapping vitamin B12 in the blood of the patients, despite a generally comparable dietary intake of the vitamin.

The lack of a threshold for the association between B12 intake and cancer argues against causality. Vitamin B12 intake was mostly within the levels seen in the general population (up to 15 µg/d) (examples are shown in Tables S4 and S5). Marley et al. found that B12 from foods, but not from foods and supplements, was associated with pancreas cancer [63]. This indicates the absence of threshold and a possible confounding by other components in the diet associated with B12, whereas, other studies did not show significant associations with food B12 or total B12 [38,64,65,66]. Associations with an intake level within the population intake range could be driven by other food components, or dietary patterns that are rich in B12.

Mendelian randomization studies have shown associations between SNPs related to high plasma B12 and cancers. This may contradict the inconsistent evidence from studies on plasma B12 concentrations. However, careful interpretation of genetic and mendelian randomization studies in term of causal inference is needed. Using genetic determinants of high B12 as an instrumental variable relies on two key assumptions: (1) the SNPs should not show direct associations with cancer; and (2) the SNPs should not show associations with the confounders. Both assumptions have not been formally investigated, but available studies suggest that these assumptions might not be fulfilled. For instance, if the genetic variants that are associated with B12 are also associated with TCN1, and if TCN1 is associated with cancer, then there is an alternative causal path between the genotype and cancer, other than that via vitamin B12.

### 4.4. Studies Needed to Clarify the Nature of the Association between High Plasma B12 and Cancer

Cohort studies with multiple follow up time points, and blood collection early enough in the course of cancer, can provide evidence about the time point when plasma B12 raise in blood during cancer progression. Studies measuring plasma B12 in subjects undergoing cancer screening programs can inform if plasma B12 is elevated in subclinical yet undiagnosed cancers. Moreover, studies should measure plasma concentrations of holoTC and MMA to distinguish between vitamin B12 status and solely elevated vitamin B12 in plasma.

The follow up measurements of vitamin B12 in patients diagnosed with cancer following different treatment regimens can clarify whether plasma vitamin B12 concentrations decline during the course of treatment. This would also mean that many patients could experience a clinically relevant vitamin B12 deficiency that needs to be treated. Well-designed placebo RCTs with vitamin B12 monotherapy are not available. These studies cannot include deficient subjects, thus also limiting their clinical relevance.

Regarding the molecular mechanisms, future studies may detect neutrophil enrichment in tumors and the co-localization of TCN1 with other structural or functional proteins or proliferative markers, to explain the role of TCN1 in cancer development (cancer progression or arrest). Moreover, studying the uptake of labelled vitamin B12 into tumor tissues versus normal tissues, and relating this to plasma concentrations of vitamin B12, may show whether plasma concentrations of the vitamins are reflecting tissue content or demands.

### 4.5. Vitamin B12 Deficiency in Patients with Cancers

Several studies have shown that low plasma vitamin B12 concentrations are associated with cancer [32,33,34,36,37]. Therefore, many patients could undergo extensive cancer treatments while having low vitamin B12 status. Low vitamin B12 status is associated with anemia and serious and irreversible neurological symptoms. The prevalence of low vitamin B12 status in patients with cancer could be underestimated. Vitamin B12 deficiency could occur despite the normal plasma concentrations of vitamin B12 and measurement of plasma concentrations of the metabolic marker, MMA, in this target group could be important to detect intracellular B12 deficiency. Since we found no firm evidence for a causal association between vitamin B12 and cancer, low vitamin B12 status in patients with cancer should be treated to prevent neurological damage and increase the tolerance of cancer treatment, such as chemotherapy.

### 4.6. Limitations of the Present Study

First, the literature search in PubMed was limited to the last 10 years. Therefore, we could have missed key studies. However, the studies that we identified are likely to be representative of the literature on the topic. Second, we did not plan to conduct a meta-analysis that would have required us to contact the authors to obtain additional data. We also did not evaluate the risk of bias and quality of the studies. However, the results of this research can be taken as a starting point for future studies to focus on common cancers and vitamin B12 biomarkers or mechanistic studies, to explain why plasma B12 concentrations could be elevated in some patients with cancer.

## 5. Conclusions

High plasma B12 concentrations were consistently associated with cancer of the liver, the organ that stores and metabolizes vitamin B12. Otherwise, the associations were inconsistent. High vitamin B12 intake was not likely to explain high plasma vitamin B12 in patients with cancer. Overall, genetically-determined high B12 concentrations showed associations with cancer. A direct relationship between genetic determinants of B12 and cancer is principally possible, although not yet proven. Randomized control trials using high dose multivitamin supplements are not suitable to judge a possible causality of B12 in cancer. In addition, studies with null results are less likely to be published (possible publication bias).

There was insufficient evidence on temporality, biologically meaningful threshold, or dose-response associations. In addition, there was no distinctive pattern of the associations with adenocarcinomas and squamous cell carcinoma. This does not support a functional role of B12 in cancer initiation or progression, but rather suggests that elevated B12 could be an epiphenomenon in some cancers. Therefore, unless the mechanisms of elevated B12 in some, but not all cancers are clarified, high plasma concentrations of B12 accidentally detected in people not receiving supplements should not be considered as a diagnostic or prognostic parameter. The conventional treatment of cancer lowered plasma B12 concentrations, suggesting that the active tumor was the source of high vitamin B12 concentrations in the circulation.

Although there are several gaps in knowledge, we regard the evidence on a causal association between high plasma vitamin B12 concentrations, vitamin B12 intake, or treatment with pharmacological doses of vitamin B12 and cancer, as insufficient. Low plasma vitamin B12 concentrations are common in patients with cancer. Due to the lack of evidence on harm, it is necessary to diagnose and treat vitamin B12 deficiency in patients with cancer who need such a treatment.

## Figures and Tables

**Table 1:** Observational studies on plasma/serum vitamin B12 concentrations (p-B12 or s-B12) or B12 intake and liver cancer.

Study, Design	Exposure/Outcome	High B12 vs. Reference Category	Results Summary	Adjustments/Limitations
Lin et al., 2010 [7], Case-control	P-B12 (and B12 intake) in relation to survival in patients with hepatocellular carcinoma 90 cases and 90 controls, “Taiwan”	Tertile T1 < 699 ng/L * = reference T2; 699–1500 ng/L T3; >1500 ng/L B12 > 699 (cut-off) vs. < 699 ng/L B12 intake did not differ by tertiles of plasma B12: in T1 2.8 ± 2.0 µg/d; in T2 2.6 ± 2.0 µg/d; in T3 2.4 ± 1.5 µg/d	HR (95%CI) of survival 2.95 (1.22–7.11) 3.24 (0.99–10.60) 2.88 (1.26–6.60) B12 intake: 3.0 ± 5.7 μg/d in patients vs. 4.3 ± 8.3 μg/d in the age and sex matched controls.	Adjustments not clear for what. High B12 associated with low albumin, hemoglobin, erythrocytes count, and alanina amino transferase, but with high alpha-fetal protein and tumor size. High B12 concentrations associated with malnourishment, liver injuries, and tumor progression.
Cui et al., 2016 [8], Case-control	P-B12 among 312 patients with hepatic cancer and 325 controls “China”	lowest Q1 (227–265) = reference Q2 (266–406) Q3 (407–589) Q4 (590–1478) pmol/L	OR (95%CI) for liver cancer 1.43 (0.72–2.81) 0.63 (0.31–1.25) 2.01 (1.02–3.98)	Adjusted for age, sex, smoking, and Hepatitis B surface antigen. Small sample size and uncontrolled confounders. Higher proportion of patients with hepatic cancer had low B12 concentrations than in the controls.
Simonsen et al., 2014 [9], Case-control	P-B12 in 120 hepatic cancers and 46 controls and 102 patients with chronic liver diseases “Denmarkand Australia”	Median (range) of p-B12 = 500 (120–1480) pmol/L in patients with liver cancer vs. 330 (140–800) pmol/L in the controls	P < 0.001	Also TCN1 was elevated in plasma of patients with chronic liver diseases and those with liver cancer.
Chang et al., 2015 [10], Case-control	P-B12 in 204 cases with liver cancer and 415 controls “China”	p-B12, pmol/L Q1 (<154) = reference Q2 (154–229) Q3 (229–324) Q4 (>324)	OR (95%CI) = 1.00 1.37 (0.59–3.16) 4.27 (2.00–9.10) 9.90 (4.80–20.44)	Adjusted for age, sex, BMI, education, smoking, H.Pylori (in stomach cancer), Hepatitis B infection and aflatoxin (in liver cancer), and other micronutrients. Found positive association between p-B12 and esophagus, stomach cancer for the Q4 vs. Q1. But the association with liver cancer was stronger.
Arendt et al., 2016 [11], Cohort	P-B12 measured in the previous year/30-day mortality post diagnosis. 327 liver cancers were identified in health registers data of B12 measurements “Denmark”	< 200 pmol/L excluded 200–600 pmol/L = reference 601–800 pmol/L > 800 pmol/L	mortality risk ratio 1.0 1.2 (0.6–2.5) 3.0 (1.7–5.3)	Adjusted for age, sex, calendar year, Charlson comorbidity score index, and cancer stage. Analyzing the data by cancer type do not consistently support that the mortality is higher in patients with high B12. Excluded all B12 levels < 200 pmol/L, possible confounding by indication, and underreported supplements.
He et al., 2022 [12], Cohort	B12 intake in relation to mortality among 905 newly diagnosed hepatic cancer patients were recruited in the Guangdong Liver Cancer Cohort “China”	Median (P25, P75) of B12 intake, µg/d Q1 0.4 (0.1, 0.7) = reference Q2 1.1 (1.0, 1.2) Q3 1.6 (1.5, 1.8) Q4 2.8 (2.3, 4.3)	Median B12 (IQR) intake in 12 months pre diagnosis of cancer 1.3 (0.9, 2.0) μg/d. HR (95% CI) for all-cause and hepatic cancer-specific mortality during the follow up of 791 days according to intake quartiles 1.04 (0.76–1.42) 0.86 (0.61–1.20) 0.83 (0.61–1.13) For hepatic cancer specific mortality 1.04 (0.76–1.42) 0.86 (0.61–1.20) 0.83 (0.61–1.13)	Adjusted for sex, age, BMI, energy intake, physical activity, and education level, smoking, alcohol drinking, presence of chronic diseases (hypertension, diabetes, dyslipidemia, fatty liver disease, and cirrhosis), Barcelona Clinic Liver Cancer stage (0, A, B, C), and treatment (surgery, other treatments).

* 0.74 to convert from ng/L to pmol/L. BMI, body mass index; CI, confidence intervals; HR, hazard ratio; OR, odds ratio; P, plasma; Q, quartile or quintile; T1 through 3, Tertiles.

**Table 2:** Evaluation of the causal link between high vitamin B12 (concentration or intake) and cancer according to Bradford Hill criteria.

Criteria	Results	Criteria Fulfillment
Strength of association	The strength of the associations varied between studies on the same type of cancer (except for liver cancer) and between types of cancer. In general, the association appears to be confounded by the tumor size, stage of cancer at diagnosis, and general health condition of the patients [7]. Adjustments for confounders were performed in some, but not all studies. The adjustments were sometimes insufficient (i.e., [31]). Residual confounding is very likely.	Not fulfilled
Consistency	The direction of the association was not consistent across all studies and all types of cancer. Additionally, low B12 was associated with cancer [32,33,34,35,36,37] and high B12 was associated with lower risk of cancer [38,39]. In studies reporting plasma B12 and MMA, or B12 and holoTC [38,39,40], the results of non-significant association or protective association with B12 were confirmed by MMA and holoTC. Studies on vitamin B12 intake as an exposure variable were also not consistent and did not support that high plasma B12 in some cancers could be due to high B12 intake. The majority of the studies on B12 concentrations or intake found non-significant associations. Nevertheless, high plasma B12 showed consistent association only with liver cancer (Table 1). This inconsistency strongly suggests that vitamin B12 itself (i.e., its role as a cofactor in one carbon metabolism and mitochondrial metabolism) is not the explanation or the cause of cancer progression. A U-shape association (high risk of cancer at low and high plasma B12) is also not supported by the results of this search.	Not fulfilled, except for liver cancer
Specificity	High plasma B12 concentration is not specific for cancers. All disorders (other than cancer) that affect the liver cause high concentrations of vitamin B12 in plasma [9]. Thus, high plasma B12 is the result of tissue and cell damage secondary to cancer or other disorders that damage the cells.	Not fulfilled
Temporality	Early stages of cancer (i.e., hyperplastic polyps [41,42] were not associated with high plasma B12 concentrations. If high plasma B12 is not present early enough in the course of cancer, it is not plausible that B12 can have a role in tumor progression (the cause must precede the effect in a due time). The health register studies consistently showing that plasma B12 concentrations in the year before cancer diagnosis is associated with higher mortality are subject to bias (Figure S3).	Not fulfilled
Biological gradient	We found no evidence of a threshold for the association between high plasma B12 or B12 intake and cancer. The threshold associated with high risk was rarely in the supraphysiological blood range, but often in the range needed for physiological body functions, which is not biologically meaningful if plasma B12 would be a risk or a safety marker. Several studies have used data-driven cut-off (i.e., quartiles or quintiles). Many of these studies reported positive associations between plasma B12 and the OR or RR of cancer within the population reference range of plasma B12. On the other hand, there could be underestimation of supplement use in observational studies making the associations subject to confounding by underreported intake. Most studies that showed positive associations between high plasma B12 or B12 intake and cancer did not show a dose-response association.	Not fulfilled
Plausibility	High plasma B12 was argued to play a role in cancer progression due to its role as a cofactor in one carbon metabolism or in the mitochondria. However, from the studies presented here, we found no support for a causal role of B12 in promoting cancer growth or death due to cancer. In addition, elevated MMA (i.e., a marker of B12 deficiency) has been linked to cancer progression [2]. When B12 is high (thus MMA is low), we would expect low MMA to be associated with cancer. But this was not the case in the studies reviewed here. TCN1 has been shown in several studies to be overexpressed in cancer tissues [13]. TCN1 carries B12 in plasma but is not responsible for delivering B12 into the cells, thus it is in theory possible that high expression of TCN1 is causing high plasma B12, while intracellular B12 is not elevated. The more likely explanation is that raised plasma B12 is caused by cancer (among other systemic disorders) and not vice versa.	Not fulfilled
Coherence	We focused on the literature since 2005. Since the early reports on the association between elevated plasma B12 and cancer, there have been many negative studies. The theory of elevated plasma B12 being causally related to cancer progression is not comprehensive regarding various aspects of the exposure-to-disease paradigm.	Not fulfilled
Experimental evidence	RCTs with B12 alone are not available. RCTs with multivitamins were not designed to answer the question and were un-blinded in one trial [29] after the intervention and before studying the outcome of cancer.	No evidence (RCTs)
Analogy	If high plasma B12 would cause cancer, then factors that increase plasma B12 would also cause cancer. This is a rather weak criterion and cannot be definitely judged in case of B12. For example, all liver damaging diseases would increase B12 and are risk factors of cancer in the same time. But they are better judged as confounders. Additionally, high B12 intake that is usually associated with higher plasma level showed even less convincing association with cancer, suggesting no analogy.	Not clear/Not fulfilled

## Data Availability

Not applicable.

## Supplementary Materials

The following supporting information can be downloaded at: https://www.mdpi.com/article/10.3390/nu14214476/s1, Figure S1: directed acyclic graph (DAG) showing the concept of studying the association between vitamin B12 and cancer. Figure S2: study flow diagram. Figure S3: directed acyclic graph (DAG) showing the association of high vitamin B12 levels and future cancers or death from cancer in studies based on health registry data. Table S1: search strings applied for PubMed. Table S2: studies excluded in the full text stage and the reasons for exclusion. Table S3: observational studies on plasma/serum B12 or B12 intake and cancers of the esophagus and stomach. Table S4: observational studies on plasma/serum B12 or B12 intake and cancers of the pancreas. Table S5: observational studies on plasma/serum B12 or B12 intake and breast and cervical cancers. All studies are case-control studies, except when otherwise indicated. Table S6: observational studies on plasma/serum B12 or B12 intake and prostate cancer. All studies are of case-control design, unless otherwise indicated. Table S7: observational studies on plasma/serum B12 or B12 intake and kidney and urothelial bladder cancers. Table S8: observational studies on plasma/serum B12 or B12 intake and colorectal cancers. Table S9: observational studies on plasma/serum B12 or B12 intake and lung cancers. Table S10: all other cancers and studies with mixed types of cancer. Table S11: studies on genetic factors and mendelian randomization Table S12: randomized controlled trials on intervention including vitamin B12 and cancer or cancer-related outcomes.
