# Supplementary material for: High Plasma Vitamin B12 and Cancer in Human Studies: A Scoping Review to Judge Causality and Alternative Explanations
Source: Nutrients. 2022 Oct 25;14(21):4476. doi: 10.3390/nu14214476 (PMC9658086; doi:10.3390/nu14214476)
Supplement: Supplementary file 1 [file nutrients-14-04476-s001.zip › nutrients-1954610-supplementary.pptx]

## Slide 1
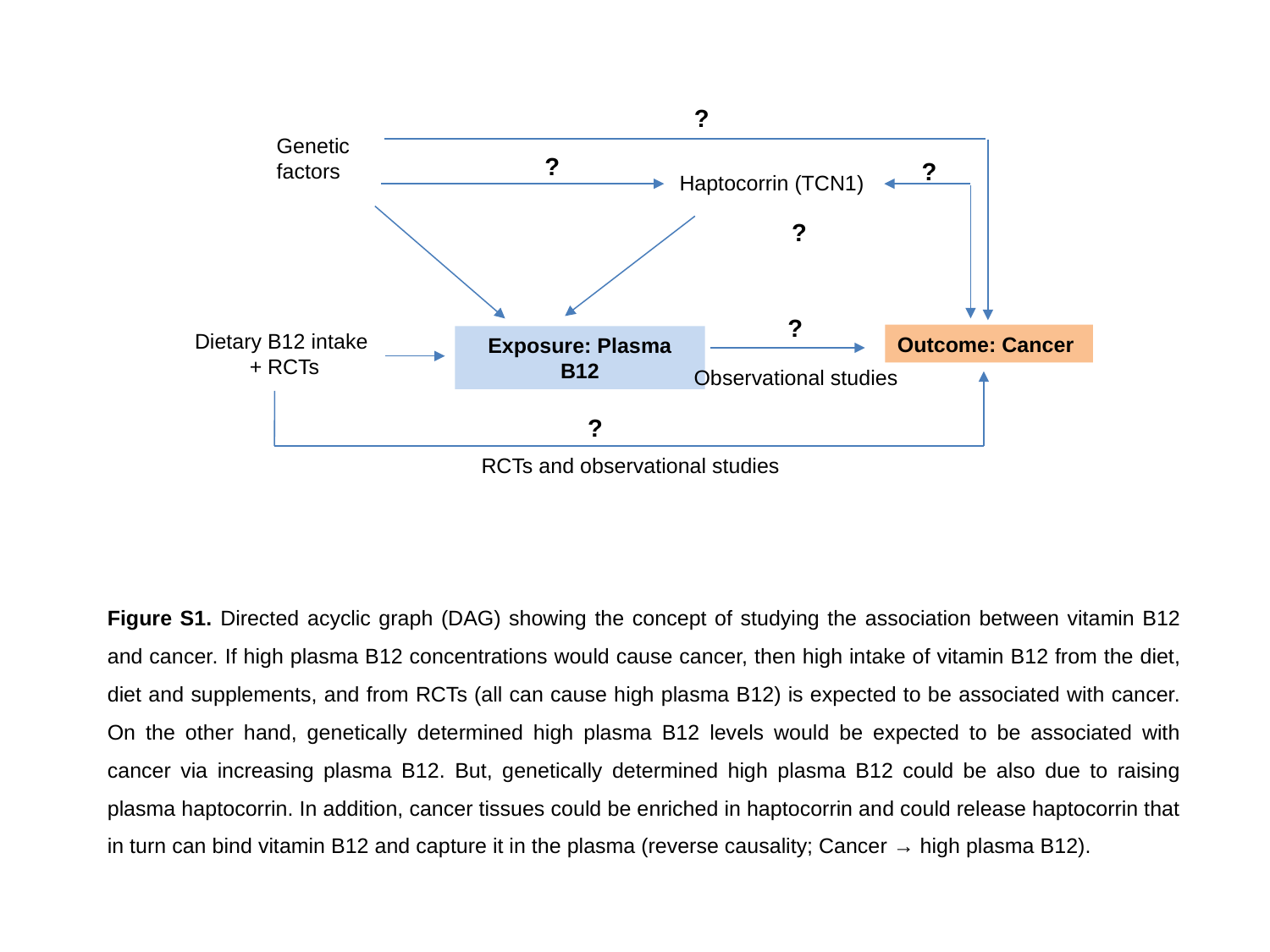

?
Genetic factors
?
?
Haptocorrin (TCN1)
?
Dietary B12 intake
+ RCTs
Outcome: Cancer
Exposure: Plasma B12
Observational studies
RCTs and observational studies
?
?
Figure S1. Directed acyclic graph (DAG) showing the concept of studying the association between vitamin B12 and cancer. If high plasma B12 concentrations would cause cancer, then high intake of vitamin B12 from the diet, diet and supplements, and from RCTs (all can cause high plasma B12) is expected to be associated with cancer. On the other hand, genetically determined high plasma B12 levels would be expected to be associated with cancer via increasing plasma B12. But, genetically determined high plasma B12 could be also due to raising plasma haptocorrin. In addition, cancer tissues could be enriched in haptocorrin and could release haptocorrin that in turn can bind vitamin B12 and capture it in the plasma (reverse causality; Cancer → high plasma B12).

## Slide 2
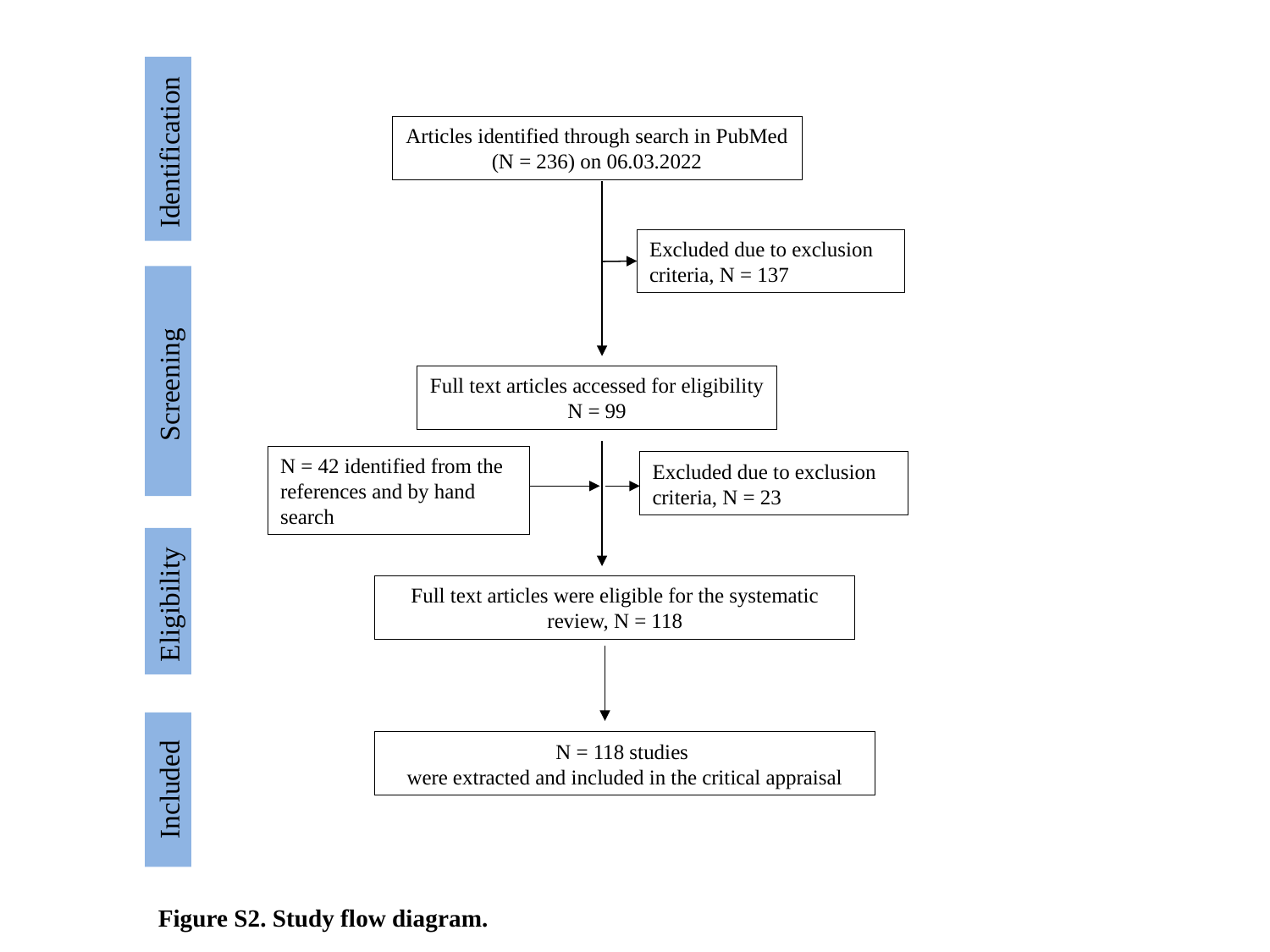

Articles identified through search in PubMed (N = 236) on 06.03.2022
Identification
Excluded due to exclusion criteria, N = 137
Screening
Full text articles accessed for eligibility
N = 99
N = 42 identified from the references and by hand search
Excluded due to exclusion criteria, N = 23
Full text articles were eligible for the systematic review, N = 118
Eligibility
N = 118 studies
were extracted and included in the critical appraisal
Included
Figure S2. Study flow diagram.

## Slide 3
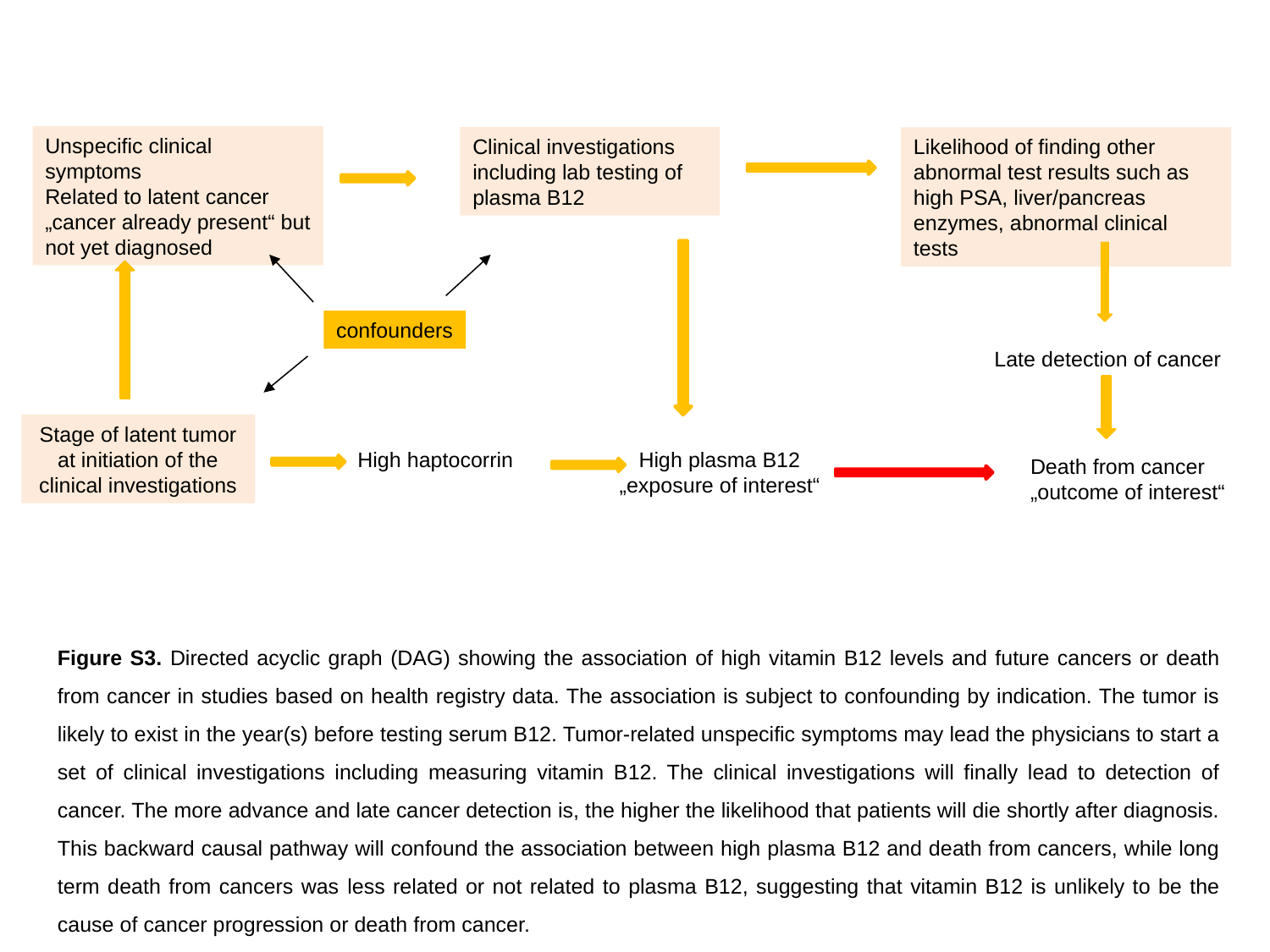

Unspecific clinical symptoms
Related to latent cancer
„cancer already present“ but not yet diagnosed
Clinical investigations including lab testing of plasma B12
Likelihood of finding other abnormal test results such as high PSA, liver/pancreas enzymes, abnormal clinical tests
confounders
Late detection of cancer
Stage of latent tumor
at initiation of the clinical investigations
High plasma B12
„exposure of interest“
High haptocorrin
Death from cancer
„outcome of interest“
Figure S3. Directed acyclic graph (DAG) showing the association of high vitamin B12 levels and future cancers or death from cancer in studies based on health registry data. The association is subject to confounding by indication. The tumor is likely to exist in the year(s) before testing serum B12. Tumor-related unspecific symptoms may lead the physicians to start a set of clinical investigations including measuring vitamin B12. The clinical investigations will finally lead to detection of cancer. The more advance and late cancer detection is, the higher the likelihood that patients will die shortly after diagnosis. This backward causal pathway will confound the association between high plasma B12 and death from cancers, while long term death from cancers was less related or not related to plasma B12, suggesting that vitamin B12 is unlikely to be the cause of cancer progression or death from cancer.
