# Supplementary material for: High Plasma Vitamin B12 and Cancer in Human Studies: A Scoping Review to Judge Causality and Alternative Explanations
Source: Nutrients. 2022 Oct 25;14(21):4476. doi: 10.3390/nu14214476 (PMC9658086; doi:10.3390/nu14214476)
Supplement: Supplementary file 1 [file nutrients-14-04476-s001.zip › Supplemental Data File B12 & Cancers 19.09.22.pdf]

Supplemental Data File

High plasma vitamin B12 and cancer in human studies: a scoping review to judge causality and alternative explanations

Author: Rima Obeid

Critical appraisal of the association between vitamin B12 and different types of cancer

1- Vitamin B12 and esophagus and stomach cancers

We identified 12 observational studies on vitamin B12 and esophagus and/or stomach cancers in addition to 2 meta-analyses that investigated the association between vitamin B12 intake and esophageal cancer [1] and gastric cancer [2]. The results are heterogeneous (**Table S3**).

Seven studies investigated the association between vitamin B12 intake and esophagus and/ or stomach cancers (3 observational studies and 1 meta-analysis showed positive results). In addition, the association between plasma/serum markers (B12 alone, B12 and holoTC, or B12 and MMA) and cancer was addressed in 7 studies (4 studies showed significant associations). One study investigated vitamin B12 intake and plasma levels of vitamin B12 and holoTC in the same population [3].

Pan et al., reported that vitamin B12 intake [median B12 intake in the controls = 5.38 (3.98–7.31) µg/d vs. in cases 5.40 (4.14–7.39) µg/d] was not significantly different between the cases and controls [3]. The same study has shown that both higher plasma B12 and higher holoTC concentrations were associated with lower OR (95%CI) for esophageal squamous cell carcinoma (i.e., high B12 protective) [3]. Three additional studies on vitamin B12 intake and esophagus and/ or stomach cancers showed no significant associations [4-6].

Khairan et al., found a positive association between vitamin B12 intake (Q4 and Q5 versus Q1) and esophageal cancer in a Japanese cohort study [7]. Subgroup analysis showed that the association was confined to people who never used alcohol, while in alcohol drinkers (low and high consumption), there were no significant associations. The study did not find associations with the source of B12 from the diet. However, the interaction between alcohol and vitamin B12-associated risk could suggest more prevalent use of underreported supplements among non-alcohol drinkers. A recent publication from the same cohort found that vitamin B12 intake was not associated with gastric cancer, even in the subgroup with high risk for gastric cancer (H. pylori antibody status and atrophic gastritis) [8].

Sharp et al., found that high vitamin B12 intake (Q4 ≥ 9.7 µg/d vs. Q1 ≤ 6.4 µg/d) was related to higher OR (95%CI) for esophageal adenocarcinoma and Barrett's esophagus, but with lower OR (95%CI) for reflux esophagitis in the third quartile versus Q1 [9]. The inconsistent results between esophageal adenocarcinoma, Barrett's esophagus, and reflux esophagitis are not biologically plausible and suggest unknown confounding or chance results. Barrett's esophagus is considered to be a premalignant condition. Patients with esophageal adenocarcinoma were more likely to consume higher amounts of red meat compared to the controls [10], suggesting that confounding by other components of the diet is possible.

Among seven studies with plasma/serum biomarkers as exposure variables, 3 case-control studies showed either a protective effect or no association between concentrations of vitamin B12 and esophageal cancer [11-13]. In contrast, four studies showed higher risk of esophageal cancer or mortality in patients with higher plasma vitamin B12 levels [12,14-16]. Additionally, a study with

parallel measurement of plasma vitamin B12 and holoTC [3] and one study with parallel measurement of vitamin B12 and MMA concentrations [13] found that high plasma B12 was associated with lower OR (95%CI) for cancer (i.e., high B12 is protective) in general or in subgroups [3,13]. In general this association was not confirmed by holoTC and MMA.

In summary, 6 observational studies (2 on B12 intake and 4 on plasma B12 concentrations) showed significantly increased risk of esophageal or gastric cancer or higher cancer-related mortality when vitamin B12 intake or concentrations were high compared to when they were low. The remaining 6 studies showed either null association or protective effects of high B12.

The meta-analysis on B12 intake and esophageal cancer showed that higher intake was associated with higher risk for esophageal cancer [OR (95%CI) = 1.30 (1.05, 1.62)] [1]. The estimates used in the meta-analysis were not adjusted for confounders (education, sex, smoking, alcohol, animal foods, etc.) and for the source of vitamin B12 in the diet. Animal foods such as red meat are good sources of vitamin B12 and in the same time have been shown to be associated with cancer risk. He et al., [2] reported no significant association between B12 intake and the risk of gastric cancer OR = 0.88 (0.69-1.12). The study even suggested that high intake of vitamin B12 might reduce the risk of gastric cancer in H. pylori-negative people OR = 0.83 (0.62-0.99), but increase the cancer risk in H. pylori-positive people OR = 1.66 (1.27-2.16) [2]. High vitamin B12 intake might increase the risk of non-cardia gastric cancer OR = 1.15 (1.01-1.33). Moreover, a decreased risk of gastric cancer at high B12 intake has been reported in non-smokers OR = 0.83 (0.71-0.96) but not in smokers [2].

If high vitamin B12 intake would cause cancer (i.e., [1]), this is expected to go through raising plasma vitamin B12 concentrations. However, there was no consistent evidence to support this hypothesis in the literature on plasma B12 combined with MMA or plasma vitamin B12 combined with holoTC concentrations. Chronic use of acid lowering-drugs in patients at risk for gastric cancer is a confounder that influences B12 absorption [17] and is likely to cause vitamin B12 deficiency. This factor was not taken into account in available observational studies on this topic.

In a cohort study, high plasma B12 measured up to 1 year before diagnosis of gastric cancer was associated with 30 days mortality after cancer diagnosis, while mortality on long term (for 31-90d mortality, p value = 0.07; and for 91-365d mortality p = 0.90) was not higher in patients with high plasma vitamin B12 [15]. Therefore, it is likely that high plasma vitamin B12 in patients with a yet undiagnosed cancer is the result of having cancer and not a contributor to the progress of the disease.

Chang et al., reported high OR for cancer among the third and fourth quartiles of plasma vitamin B12 concentrations (Q3 229-324 pmol/L, and Q4 > 324 pmol/L) [14]. These levels are largely within the population reference range, which is not biologically meaningful (lack of threshold). In addition, the associations between B12 intake and cancer are subject to confounding due to many other lifestyle factors associated with high B12 intake (from foods and supplements). For example, Jessri, et al., reported large differences between cases and controls with regard to dietary patterns (hot foods and beverages and fried and barbecued meals) that could be independently associated with cancer [4].

Taken together, the results on the association between vitamin B12 intake or plasma biomarkers and esophagus and/ or stomach cancers were mixed and largely inconsistent between the studies. Inconsistent results were also observed for the two related exposures; B12 intake and plasma B12 concentrations. The results of the meta-analysis showing associations between vitamin B12 intake and cancer are not in line with the majority of observational studies on plasma vitamin B12 concentrations and esophageal cancer, suggesting that other dietary components associated with high B12 intake might drive these associations between B12 intake and cancer. Overall, there is no support for a causal association between vitamin B12 intake or status biomarkers on the one hand and esophagus and/ or stomach cancers on the other hand. In addition, esophagus and/ or stomach cancers could be associated with low vitamin B12 concentrations [i.e., [3]] that need to be treated to prevent subsequent development of neurological or hematological symptoms in the patients who are already fragile due to cancer and cancer-related therapies.

2- Vitamin B12 and pancreas cancer

Nine observational studies reported on the association between vitamin B12 and pancreas cancer and 1 meta-analysis of observational studies (**Table S4**). Four studies were on plasma/serum vitamin B12 concentrations and 6 studies on B12 intake as exposure variables. The meta-analysis included 6 studies on B12 intake and 3 studies on plasma B12 concentrations [18]. This meta-analysis reported none-significant associations [18].

Three of the 4 studies on plasma vitamin B12 concentrations found no association between plasma B12 and pancreas cancer [19-21]. Whereas, Arendt et al., reported increased risk of 30-days mortality after diagnosing pancreas cancer in patients with elevated vitamin B12 concentrations [15]. Of the 6 studies on vitamin B12 intake [21-26], one study found higher vitamin B12 intake (from foods but not total intake from food and supplements) to be associated with pancreas cancer [26].

The inconsistency between results on vitamin B12 intake from foods and foods plus supplements in the study of Gong et al., [26] does not support a dose-response relationship or the presence of a threshold. The study with parallel measurements of vitamin B12 intake (from foods and foods plus supplements) and B12 concentrations in the same participants did not find significant associations of the intakes or the concentrations with pancreas cancer [21].

Overall, there is no consistent evidence that high vitamin B12 intake or plasma concentrations are associated with pancreas cancer.

3- Breast, ovarian and cervical cancers

We identified 17 studies on vitamin B12 and breast cancer including one meta-analysis and 1 study on ovarian cancer (**Table S5**). 7 of 17 observational studies were on plasma/serum B12 concentrations and breast cancer [15,27-32] and 11 were on B12 intake (one study reported intake and plasma levels [27]) as exposure variables [27,33-42]. The meta-analysis included 4 studies on plasma levels and 14 studies on B12 intake [43].

Women recently diagnosed with breast cancer and high plasma vitamin B12 concentrations detected one year prior to cancer diagnosis showed higher 30 days mortality compared to women with lower B12 concentrations [15]. All of the remaining studies measuring plasma/serum vitamin B12 concentrations as an exposure variable reported no significant associations between plasma/serum vitamin B12 and cancer (or no consistent associations across the vitamin B12 percentiles [27-32].

Three studies reported lower risk of breast cancer in women (or subgroups of women, eg. MTHFR TT) with higher vitamin B12 intake (versus lower intake) [33,39,44]. All other studies on vitamin B12 intake and breast cancer, except one study [45], reported no significant associations between B12 intake and the risk of breast cancer in the fully adjusted models. Wu et al, reported no significant association between B12 intake or serum concentrations and breast cancer in their meta-analysis [43].

Five studies considered plasma/serum vitamin B12 concentrations as an exposure variable in relation to the risk of cervical cancer, while 1 study measured both plasma concentrations and intake of B12 in the same time. The 5 studies reported either lower risk of cervical cancer at high plasma B12 concentrations or no significant associations (4 on B12 levels and 1 included additionally intake) [46-50].

Piyathilake et al., reported higher percentage of women with cervical cancers with low plasma vitamin B12 concentrations (< 200 pg/ml) compared to the controls (18.4 versus 10.3 %) [49], suggesting that physicians need to be aware of vitamin B12 deficiency in this group and accordingly provide supplemental vitamin B12 to prevent comorbidities due to deficiency. Three studies on cervical cancers were of small sample size and included Asian populations (India, Thailand, and Korea) [46-48] who may have had low vitamin B12 status on long term due to lifestyle or cultural factors. A recent Chinese cohort study found no significant association between B12 intake and ovarian cancer, but the intake in this study was remarkably lower than in other studies [37].

Thus, most of the studies (though not all) reported either non-significant association or inverse associations between B12 (intake or blood concentrations) and breast, ovarian or cervical cancers.

4- Prostate cancer

We identified 11 studies including one meta-analysis addressing the association between concentrations of serum/plasma B12 (or holoTC) or vitamin B12-binding proteins and prostate cancer. Three partly overlapping studies reported high serum vitamin B12 concentrations to be associated with prostate cancer [15,51,52]. 5 studies found no association between serum vitamin B12 concentrations and prostate cancer [31,53-56] (**Table S6**).

de Vogel et al., found no significant association between high serum B12 or low MMA concentrations and prostate cancer [53]. Hultdin et al., reported significantly higher OR (95%CI) for prostate cancer at higher serum vitamin B12 concentrations [52,52,52], but the study did not adjust for potential confounders and the associations were seen within the population reference range (Q2 238-302, Q3 302-370, Q4 > 370 pmol/L) vs. Q1 (< 238 pmol/L) [52] which is not biologically meaningful. Collin et al., reported higher serum haptocorrin, but not serum B12 or holoTC concentrations to be associated with higher OR for prostate cancer [54]. Moreover, concentrations of serum B12 and its binding proteins were not associated with concentrations of prostate specific antigen velocity (a surrogate marker for prostate cancer progression) in patients with prostate cancer [56]. In contrast, higher serum B12 was associated with higher OR for prostate cancer in one analysis of individual patient's data [51] and a meta-analysis [54] [i.e., pooled OR = 1.10 (1.01-1.19) per 100 pmol/L higher B12].

Vidal et al., found no significant association between vitamin B12 intake > 25μg/d (vs. ≤ 25 μg/d) and prostate cancer in general [57]. There were also no associations among subgroups of patients with high grade or low-grade prostate cancer.

In general, there is only limited evidence that high vitamin B12 could be associated with prostate cancer and this association could be linked to elevated TCN1. However, some studies were subject to confounding and vitamin B12 levels were not related to a prognostic marker of prostate cancer, suggesting that vitamin B12 levels have no prognostic values.

5- Kidney and bladder cancers

Ten studies were identified including one meta-analysis on vitamin B12 and kidney or bladder cancers [15,58-65]. 7 of 10 studies measured B12 plasma levels and 6 studies including one meta-analysis considered B12 intake as an exposure [61,62,64-66] (**Table S7**).

1 of 7 studies on plasma levels reported higher B12 levels to be associated with cancer outcome (30-days mortality) [15]. In contrast, 1 study reported lower OR (95%CI) for renal cancer, but no association with HR for all-cause mortality of cancer [60]. In a large pooled data analysis from 4 cohort studies (2915 bladder cancer cases and 530,012 non-cases), Boot et al., found a rather protective effect of high intake of vitamin B12 in men and women versus low intakes on the risk of bladder cancer [66]. The meta-analysis included only 2 studies on serum B12 and 2 studies on B12 intake and did not find significant associations [65]. All other studies on vitamin B12 intake or plasma concentrations and renal or bladder cancer did not show significant associations.

Therefore, the majority of the observational studies did not show significant associations between high serum/plasma B12 concentrations and renal or bladder cancers, one study showed inverse association between B12 intake and bladder cancer risk and one study showed higher mortality risk at high plasma B12.

6- Colorectal cancers

We identified 11 observational studies (9 on plasma B12 markers and 2 on tissue TCN1); 2 studies [15,67] showed that higher plasma B12 concentrations were associated with colon cancer or cancer-related outcomes. Bystrom et al., found that patients with low vitamin B12 concentrations (< 300 pmol/L) had better prognosis [67], while Arendt et al., found higher 30-day mortality in patients recently diagnosed with colon cancer and had high plasma B12 concentrations measured in the year before the diagnoses [15] (**Table S8**).

In contrast, lower plasma B12 concentrations were observed in patients with colon cancer than in the controls [68,69]. While the remaining studies found no significant associations between plasma B12 concentrations and colon cancer [70-74].

Hyperplastic polyps are considered to be a pre-cancer stage. Two studies found no association between serum vitamin B12 and hyperplastic polyps, suggesting that high serum B12 is not likely to occur long time before cancer is diagnosed [68,74], thus providing evidence against the temporality that is an important causal criterion.

The causes of high serum vitamin B12 concentrations among patients with cancer are not known. Two studies have provided clues towards mechanisms. Liu et al., studied mRNA expression of TCN1 in colon cancer tissues and adjacent tissues and found that TCN1 gene expression is upregulated in tumor cells and is related to cellular events that are typical to cancer such as apoptosis and inflammation [75]. In line with this, Zhu et al., have shown that higher staining of TCN1 was related to larger and invasive tumors, higher tumor markers, and metastasis to regional lymph, while lower staining of TCN1 in tissues was related to significantly longer survival of the patients compared to the medium and high TCN1 expression groups [76]. Patients with low, medium, and high levels of TCN1 immunoexpression had different 5-year survival (88.9%, 50.0%, and 40.0%, respectively) with lowest survival at higher tissue expression of TCN1 [76]. Also high expression of TCN1 in colon cancer tissues predicted worse prognosis and less response to neoadjuvant chemosensitivity of colon cancer [75]. Therefore, TCN1 is upregulated in larger and more aggressive tumors. In general, TCN1 is not responsible for delivering B12 into the cells. If TCN1 is released from tumor tissues, it is theoretically available to capture more vitamin B12 and cause high plasma vitamin B12 in some cancers. Vitamin B12 is delivered into the cells bound to transcobalamin (as holoTC) via the cellular transcobalamin receptor. The function of TCN1 in tumor tissue is not known, but deserves more investigations.

In summary, the majority of the studies have shown no association between plasma vitamin B12 concentrations and colorectal cancer. Two studies reported higher serum B12 to be associated with colon cancer. There was no evidence that patients with polyps (pre-stage of cancer) have higher vitamin B12 concentrations suggesting that high serum B12 is not likely to exist in an early stage of cancer and thus it is unlikely to contribute to cancer progression. Tumor tissues express high level of TCN1. The degree of TCN1 expression is associated with cancer grade and worse progression while the function of this B12-transporting protein in the pathophysiology of cancer is not clear.

7- Lung cancers

Six studies addressed the association between B12 and lung cancer [15,77-81] (**Table S9**).

Fanidi et al., found that higher serum B12 concentrations are associated with higher OR for overall lung cancer, adenocarcinoma and large cell carcinoma [78]. However, significant associations were reported within the physiological range of B12 [i.e., Q3 426-531 pmol/L], which is not biologically meaningful. Arendt et al., found higher 30-day mortality after diagnosis of cancer in patients with high B12 concentrations [15]. The study of Lo-Bisgaard et al did not find significant associations with concentrations of B12 or holoTC, but found that higher plasma levels of haptocorrin and transcobalamin (B12-binding proteins) were associated with lung cancer [77]. This is in line with studies on colon cancer where tissue expression of TCN1 was elevated [75,76]. A recent study

suggested that tissue expression of TCN1 could be a prognostic marker that distinguishes between lung adenocarcinoma and non-lung adenocarcinoma samples [82].

Brasky et al., found that high B12 intake (> 55 µg/d) was associated with lung cancer in men, but not in women [80]. However, this study is subject to recall bias since the information on B12 intake was self-reported using questionnaires covering the last 10 years [80]. In addition, people who remained free of cancer differed in many health and lifestyle aspects from those who developed cancer, suggesting that the association between vitamin B12 intake and cancer could be driven by confounders that influence the intake of the vitamin and the risk of cancer in the same time. A Chinese study on B12 intake and overall lung cancer reported higher HR (95%CI) of lung cancer in people with higher intake of B12 [81]. This association was significant in the subgroup of men, patients with adenocarcinoma and in patients with a shorter follow up time of ≤ 2 years. No associations were found in women, by smoking status or among patients followed for longer than 2 years [81]. However, vitamin B12 intakes were rather low in this study [mean ± SD = Q3 2.28 ± 0.11; Q4 2.70 ± 0.14; and Q5 3.61 ± 0.71 µg/d] vs. 2.33 ± 0.89 µg/d in the lowest quintile [81].

8- Other cancers or mixed cancers

We identified 19 studies on the association between vitamin B12 and mixed or rare types of cancer including one meta-analysis. The results were mixed (no association, significant increase in the risk of cancer, and protective effect of high B12) (**Table S10**).

Several studies showed that low rather than high serum or plasma concentrations of B12 were associated with cancer [83-87]. Three studies with low number of participants investigated serum/plasma vitamin B12 concentrations in patients with head and neck cancers [85,88,89]. One study found that low B12 concentrations were associated with head cancers [85] and the other 2 studies found no significant associations. A recent cohort study reported no association between higher vitamin B12 intake and cancer mortality among men or women, and according to subgroups of age, smoking or BMI [90].

A recent study in hypopharyngeal squamous cell carcinoma and head and neck squamous cell carcinoma with poor prognosis, found higher TCN1 expression in patients with lower sensitivity to neoadjuvant chemotherapy, indicating that low TCN1 expression predicts better neoadjuvant chemotherapy treatment response. TCN1 has been suggested as an independent prognostic biomarker for both overall survival and disease-free survival in patients with advanced cancers [91]. Silencing of TCN1 using siRNA sensitized FaDu cells to cisplatin treatment with increased cell apoptosis, suggesting that tissue expression of TCN1 (secretory protein) could be used for personalized treatment of cancers [91].

Taken together, the results of studies on mixed cases with cancer confirm the inconsistency of the association reported in our specific types of cancers.

Reference List

1. Qiang Y., Li Q., Xin Y., Fang X., Tian Y., Ma J., Wang J., Wang Q., Zhang R., Wang J. et al. Intake of Dietary One-Carbon Metabolism-Related B Vitamins and the Risk of Esophageal Cancer: A Dose-Response Meta-Analysis. Nutrients. **2018**, 10.

2. He J., Fu H., Li C., Deng Z., Chang H. Association between Vitamin B12 and Risk of Gastric Cancer: A Systematic Review and Meta-Analysis of Epidemiological Studies. Nutr. Cancer **2022**, 1-11.

3. Pan D., Wang S., Su M., Sun G., Zhu X., Ghahvechi C.M., Guo Z., Wang N., Zhang Z., Cui M. Vitamin B12 may play a preventive role in esophageal precancerous lesions: a case-control study based on markers in blood and 3-day duplicate diet samples. Eur. J. Nutr. **2021**, 60, 3375-3386.

4. Jessri M., Rashidkhani B., Hajizadeh B., Jessri M., Gotay C. Macronutrients, vitamins and minerals intake and risk of esophageal squamous cell carcinoma: a case-control study in Iran. Nutr. J. **2011**, 10, 137.

5. Xiao Q., Freedman N.D., Ren J., Hollenbeck A.R., Abnet C.C., Park Y. Intakes of folate, methionine, vitamin B6, and vitamin B12 with risk of esophageal and gastric cancer in a large cohort study. Br. J. Cancer **2014**, 110, 1328-1333.

6. Ibiebele T.I., Hughes M.C., Pandeya N., Zhao Z., Montgomery G., Hayward N., Green A.C., Whiteman D.C., Webb P.M. High intake of folate from food sources is associated with reduced risk of esophageal cancer in an Australian population. J. Nutr. **2011**, 141, 274-283.

7. Khairan P., Sobue T., Eshak E.S., Zha L., Kitamura T., Sawada N., Iwasaki M., Inoue M., Yamaji T., Shimazu T. et al. Association of dietary intakes of vitamin B12, vitamin B6, folate, and methionine with the risk of esophageal cancer: the Japan Public Health Center-based (JPHC) prospective study. BMC. Cancer **2021**, 21, 982.

8. Khairan P., Sobue T., Eshak E.S., Zha L., Kitamura T., Sawada N., Iwasaki M., Inoue M., Yamaji T., Iso H. et al. Association of B Vitamins and Methionine Intake with the Risk of Gastric Cancer: The Japan Public Health Center-based Prospective Study. Cancer Prev. Res. (Phila) **2022**, 15, 101-110.

9. Sharp L., Carsin A.E., Cantwell M.M., Anderson L.A., Murray L.J. Intakes of dietary folate and other B vitamins are associated with risks of esophageal adenocarcinoma, Barrett's esophagus, and reflux esophagitis. J. Nutr. **2013**, 143, 1966-1973.

10. O'Doherty M.G., Cantwell M.M., Murray L.J., Anderson L.A., Abnet C.C. Dietary fat and meat intakes and risk of reflux esophagitis, Barrett's esophagus and esophageal adenocarcinoma. Int. J. Cancer **2011**, 129, 1493-1502.

11. Ren J., Murphy G., Fan J., Dawsey S.M., Taylor P.R., Selhub J., Qiao Y., Abnet C.C. Prospective study of serum B vitamins levels and oesophageal and gastric cancers in China. Sci. Rep. **2016**, 6, 35281.

12. Miranti E.H., Stolzenberg-Solomon R., Weinstein S.J., Selhub J., Mannisto S., Taylor P.R., Freedman N.D., Albanes D., Abnet C.C., Murphy G. Low vitamin B12 increases risk of gastric cancer: A prospective study of one-carbon metabolism nutrients and risk of upper gastrointestinal tract cancer. Int. J. Cancer **2017**, 141, 1120-1129.

13. Vollset S.E., Igland J., Jenab M., Fredriksen A., Meyer K., Eussen S., Gjessing H.K., Ueland P.M., Pera G., Sala N. et al. The association of gastric cancer risk with plasma folate, cobalamin, and methylenetetrahydrofolate reductase polymorphisms in the European Prospective Investigation into Cancer and Nutrition. Cancer Epidemiol. Biomarkers Prev. **2007**, 16, 2416-2424.

14. Chang S.C., Goldstein B.Y., Mu L., Cai L., You N.C., He N., Ding B.G., Zhao J.K., Yu S.Z., Heber D. et al. Plasma folate, vitamin B12, and homocysteine and cancers of the esophagus, stomach, and liver in a Chinese population. Nutr Cancer **2015**, 67, 212-223.

15. Arendt J.F., Farkas D.K., Pedersen L., Nexo E., Sorensen H.T. Elevated plasma vitamin B12 levels and cancer prognosis: A population-based cohort study. Cancer Epidemiol. **2016**, 40, 158-165.

16. Mayne S.T., Risch H.A., Dubrow R., Chow W.H., Gammon M.D., Vaughan T.L., Farrow D.C., Schoenberg J.B., Stanford J.L., Ahsan H. et al. Nutrient intake and risk of subtypes of esophageal and gastric cancer. Cancer Epidemiol. Biomarkers Prev. **2001**, 10, 1055-1062.

17. Jung S.B., Nagaraja V., Kapur A., Eslick G.D. Association between vitamin B12 deficiency and long-term use of acid-lowering agents: a systematic review and meta-analysis. Intern Med J. **2015**, 45, 409-416.

18. Wei D.H., Mao Q.Q. Vitamin B6, vitamin B12 and methionine and risk of pancreatic cancer: a meta-analysis. Nutr. J. **2020**, 19, 111.

19. Chuang S.C., Stolzenberg-Solomon R., Ueland P.M., Vollset S.E., Middtun O., Olsen A., Tjonneland A., Overvad K., Boutron-Ruault M.C., Morois S. et al. A U-shaped relationship between plasma folate and pancreatic cancer risk in the European Prospective Investigation into Cancer and Nutrition. Eur J Cancer **2011**.

20. Stolzenberg-Solomon R.Z., Albanes D., Nieto F.J., Hartman T.J., Tangrea J.A., Rautalahti M., Sehlub J., Virtamo J., Taylor P.R. Pancreatic cancer risk and nutrition-related methyl-group availability indicators in male smokers. J. Natl. Cancer Inst. **1999**, 91, 535-541.

21. Schernhammer E., Wolpin B., Rifai N., Cochrane B., Manson J.A., Ma J., Giovannucci E., Thomson C., Stampfer M.J., Fuchs C. Plasma folate, vitamin B6, vitamin B12, and homocysteine and pancreatic cancer risk in four large cohorts. Cancer Res **2007**, 67, 5553-5560.

22. Stolzenberg-Solomon R.Z., Pietinen P., Barrett M.J., Taylor P.R., Virtamo J., Albanes D. Dietary and other methyl-group availability factors and pancreatic cancer risk in a cohort of male smokers. Am. J Epidemiol. **2001**, 153, 680-687.

23. Huang J.Y., Butler L.M., Wang R., Jin A., Koh W.P., Yuan J.M. Dietary Intake of One-Carbon Metabolism-Related Nutrients and Pancreatic Cancer Risk: The Singapore Chinese Health Study. Cancer Epidemiol. Biomarkers Prev. **2016**, 25, 417-424.

24. Marley A.R., Fan H., Hoyt M.L., Anderson K.E., Zhang J. Intake of methyl-related nutrients and risk of pancreatic cancer in a population-based case-control study in Minnesota. Eur. J. Clin. Nutr. **2018**, 72, 1128-1135.

25. Jansen R.J., Robinson D.P., Frank R.D., Anderson K.E., Bamlet W.R., Oberg A.L., Rabe K.G., Olson J.E., Sinha R., Petersen G.M. et al. Fatty acids found in dairy, protein and unsaturated fatty acids are associated with risk of pancreatic cancer in a case-control study. Int. J. Cancer **2014**, 134, 1935-1946.

26. Gong Z., Holly E.A., Bracci P.M. Intake of folate, vitamins B6, B12 and methionine and risk of pancreatic cancer in a large population-based case-control study. Cancer Causes Control **2009**, 20, 1317-1325.

27. Lin J., Lee I.M., Cook N.R., Selhub J., Manson J.E., Buring J.E., Zhang S.M. Plasma folate, vitamin B-6, vitamin B-12, and risk of breast cancer in women. Am. J Clin Nutr. **2008**, 87, 734-743.

28. Matejcic M., de B.J., Ricci C., Biessy C., Perrier F., Huybrechts I., Weiderpass E., Boutron-Ruault M.C., Cadeau C., His M. et al. Biomarkers of folate and vitamin B12 and breast cancer risk: report from the EPIC cohort. Int. J. Cancer **2017**, 140, 1246-1259.

29. Agnoli C., Grioni S., Krogh V., Pala V., Allione A., Matullo G., Di G.C., Tagliabue G., Pedraglio S., Garrone G. et al. Plasma Riboflavin and Vitamin B-6, but Not Homocysteine, Folate, or Vitamin B-12, Are Inversely Associated with Breast Cancer Risk in the European Prospective Investigation into Cancer and Nutrition-Varese Cohort. J. Nutr. **2016**, 146, 1227-1234.

30. Houghton S.C., Eliassen A.H., Zhang S.M., Selhub J., Rosner B.A., Willett W.C., Hankinson S.E. Plasma B-vitamin and one-carbon metabolites and risk of breast cancer before and after folic acid fortification in the United States. Int. J. Cancer **2019**, 144, 1929-1940.

31. Essen A., Santaolalla A., Garmo H., Hammar N., Walldius G., Jungner I., Malmstrom H., Holmberg L., Van H.M. Baseline serum folate, vitamin B12 and the risk of prostate and breast cancer using data from the Swedish AMORIS cohort. Cancer Causes Control **2019**, 30, 603-615.

32. Kim S.J., Zuchniak A., Sohn K.J., Lubinski J., Demsky R., Eisen A., Akbari M.R., Kim Y.I., Narod S.A., Kotsopoulos J. Plasma folate, vitamin B-6, and vitamin B-12 and breast cancer risk in B. Am. J. Clin. Nutr. **2016**, 104, 671-677.

33. Hatami M., Vahid F., Esmaeil A.M., Sadeghi M., Ameri F., Eini-Zeinab H., Jamshidi-Naeini Y., Hossein D.S. The Vitamins Involved in One-Carbon Metabolisms are Associated with Reduced Risk of Breast Cancer in Overall and Subtypes. Int. J. Vitam. Nutr. Res. **2020**, 90, 131-140.

34. Maruti S.S., Ulrich C.M., White E. Folate and one-carbon metabolism nutrients from supplements and diet in relation to breast cancer risk. Am. J Clin Nutr. **2009**, 89, 624-633.

35. Ma E., Iwasaki M., Kobayashi M., Kasuga Y., Yokoyama S., Onuma H., Nishimura H., Kusama R., Tsugane S. Dietary intake of folate, vitamin B2, vitamin B6, vitamin B12, genetic polymorphism of related enzymes, and risk of breast cancer: a case-control study in Japan. Nutr. Cancer **2009**, 61, 447-456.

36. Ma E., Iwasaki M., Junko I., Hamada G.S., Nishimoto I.N., Carvalho S.M., Motola J., Jr., Laginha F.M., Tsugane S. Dietary intake of folate, vitamin B6, and vitamin B12, genetic polymorphism of related enzymes, and risk of breast cancer: a case-control study in Brazilian women. BMC. Cancer **2009**, 9, 122.

37. Xu H.L., Gong T.T., Liu F.H., Wei Y.F., Chen H.Y., Yan S., Zhao Y.H., Gao S., Jiao Y.S., Wu Q.J. Pre-diagnosis Dietary One-Carbon Metabolism Micronutrients Consumption and Ovarian Cancer Survival: A Prospective Cohort Study. Front Nutr. **2022**, 9, 873249.

38. Chou Y.C., Chu C.H., Wu M.H., Hsu G.C., Yang T., Chou W.Y., Huang H.P., Lee M.S., Yu C.P., Yu J.C. et al. Dietary intake of vitamin B(6) and risk of breast cancer in Taiwanese women. J. Epidemiol. **2011**, 21, 329-336.

39. Yang D., Baumgartner R.N., Slattery M.L., Wang C., Giuliano A.R., Murtaugh M.A., Risendal B.C., Byers T., Baumgartner K.B. Dietary intake of folate, B-vitamins and methionine and breast cancer risk among Hispanic and non-Hispanic white women. PLoS. ONE. **2013**, 8, e54495.

40. Shrubsole M.J., Shu X.O., Li H.L., Cai H., Yang G., Gao Y.T., Gao J., Zheng W. Dietary B vitamin and methionine intakes and breast cancer risk among Chinese women. Am. J Epidemiol. **2011**, 173, 1171-1182.

41. Stevens V.L., McCullough M.L., Sun J., Gapstur S.M. Folate and other one-carbon metabolism-related nutrients and risk of postmenopausal breast cancer in the Cancer Prevention Study II Nutrition Cohort. Am. J Clin Nutr. **2010**, 91, 1708-1715.

42. Zhang C.X., Ho S.C., Chen Y.M., Lin F.Y., Fu J.H., Cheng S.Z. Dietary folate, vitamin B6, vitamin B12 and methionine intake and the risk of breast cancer by oestrogen and progesterone receptor status. Br. J. Nutr. **2011**, 106, 936-943.

43. Wu W., Kang S., Zhang D. Association of vitamin B6, vitamin B12 and methionine with risk of breast cancer: a dose-response meta-analysis. Br. J. Cancer **2013**, 109, 1926-1944.

44. Maruti S.S., Ulrich C.M., Jupe E.R., White E. MTHFR C677T and postmenopausal breast cancer risk by intakes of one-carbon metabolism nutrients: a nested case-control study. Breast Cancer Res **2009**, 11, R91.

45. Bassett J.K., Baglietto L., Hodge A.M., Severi G., Hopper J.L., English D.R., Giles G.G. Dietary intake of B vitamins and methionine and breast cancer risk. Cancer Causes Control **2013**, 24, 1555-1563.

46. Pathak S., Bajpai D., Banerjee A., Bhatla N., Jain S.K., Jayaram H.N., Singh N. Serum one-carbon metabolites and risk of cervical cancer. Nutr. Cancer **2014**, 66, 818-824.

47. Ragasudha P.N., Thulaseedharan J.V., Wesley R., Jayaprakash P.G., Lalitha P., Pillai M.R. A case-control nutrigenomic study on the synergistic activity of folate and vitamin B12 in cervical cancer progression. Nutr. Cancer **2012**, 64, 550-558.

48. Kwanbunjan K., Saengkar P., Cheeramakara C., Tangjitgamol S., Chitcharoenrung K. Vitamin B12 status of Thai women with neoplasia of the cervix uteri. Southeast Asian J. Trop. Med Public Health **2006**, 37 Suppl 3, 178-183.

49. Piyathilake C.J., Macaluso M., Alvarez R.D., Bell W.C., Heimburger D.C., Partridge E.E. Lower risk of cervical intraepithelial neoplasia in women with high plasma folate and sufficient vitamin B12 in the post-folic acid fortification era. Cancer Prev. Res. (Phila) **2009**, 2, 658-664.

50. Tong S.Y., Kim M.K., Lee J.K., Lee J.M., Choi S.W., Friso S., Song E.S., Lee K.B., Lee J.P. Common polymorphisms in methylenetetrahydrofolate reductase gene are associated with risks of cervical intraepithelial neoplasia and cervical cancer in women with low serum folate and vitamin B12. Cancer Causes Control **2011**, 22, 63-72.

51. Price A.J., Travis R.C., Appleby P.N., Albanes D., Barricarte G.A., Bjorge T., Bueno-de-Mesquita H.B., Chen C., Donovan J., Gislefoss R. et al. Circulating Folate and Vitamin B and Risk of Prostate Cancer: A Collaborative Analysis of Individual Participant Data from Six Cohorts Including 6875 Cases and 8104 Controls. Eur. Urol. **2016**.

52. Hultdin J., Van G.B., Bergh A., Hallmans G., Stattin P. Plasma folate, vitamin B12, and homocysteine and prostate cancer risk: a prospective study. Int J Cancer **2005**, 113, 819-824.

53. de Vogel S., Meyer K., Fredriksen A., Ulvik A., Ueland P.M., Nygard O., Vollset S.E., Tell G.S., Tretli S., Bjorge T. Serum folate and vitamin B12 concentrations in relation to prostate cancer

risk--a Norwegian population-based nested case-control study of 3000 cases and 3000 controls within the JANUS cohort. Int J Epidemiol. **2013**, 42, 201-210.

54. Collin S.M. Folate and B12 in prostate cancer. Adv. Clin Chem **2013**, 60, 1-63.

55. Johansson M., Appleby P.N., Allen N.E., Travis R.C., Roddam A.W., Egevad L., Jenab M., Rinaldi S., Kiemeney L.A., Bueno-de-Mesquita H.B. et al. Circulating concentrations of folate and vitamin B12 in relation to prostate cancer risk: results from the European Prospective Investigation into Cancer and Nutrition study. Cancer Epidemiol. Biomarkers Prev. **2008**, 17, 279-285.

56. Collin S.M., Metcalfe C., Refsum H., Lewis S.J., Smith G.D., Cox A., Davis M., Marsden G., Johnston C., Lane J.A. et al. Associations of folate, vitamin B12, homocysteine, and folate-pathway polymorphisms with prostate-specific antigen velocity in men with localized prostate cancer. Cancer Epidemiol. Biomarkers Prev. **2010**, 19, 2833-2838.

57. Vidal A.C., Grant D.J., Williams C.D., Masko E., Allott E.H., Shuler K., McPhail M., Gaines A., Calloway E., Gerber L. et al. Associations between Intake of Folate, Methionine, and Vitamins B-12, B-6 and Prostate Cancer Risk in American Veterans. J. Cancer Epidemiol. **2012**, 2012, 957467.

58. Ben Fradj M.K., Mrad D.K., Kallel A., Bibi M., Ben R.S., Sanhaji H., Nouira Y., Feki M. Interaction Effects of Plasma Vitamins A, E, D, B9, and B12 and Tobacco Exposure in Urothelial Bladder Cancer: A Multifactor Dimensionality Reduction Analysis. Nutr. Cancer **2019**, 71, 1382-1389.

59. Vrieling A., Bueno-de-Mesquita H.B., Ros M.M., Kampman E., Aben K.K., Buchner F.L., Jansen E.H., Roswall N., Tjonneland A., Boutron-Ruault M.C. et al. One-carbon metabolism biomarkers and risk of urothelial cell carcinoma in the European prospective investigation into cancer and nutrition. Int. J. Cancer **2019**, 145, 2349-2359.

60. Johansson M., Fanidi A., Muller D.C., Bassett J.K., Midttun O., Vollset S.E., Travis R.C., Palli D., Mattiello A., Sieri S. et al. Circulating biomarkers of one-carbon metabolism in relation to renal cell carcinoma incidence and survival. J. Natl. Cancer Inst. **2014**, 106.

61. Gibson T.M., Weinstein S.J., Mayne S.T., Pfeiffer R.M., Selhub J., Taylor P.R., Virtamo J., Albanes D., Stolzenberg-Solomon R. A prospective study of one-carbon metabolism biomarkers and risk of renal cell carcinoma. Cancer Causes Control **2010**, 21, 1061-1069.

62. Bock C.H., Ruterbusch J.J., Holowatyj A.N., Steck S.E., Van Dyke A.L., Ho W.J., Cote M.L., Hofmann J.N., Davis F., Graubard B.I. et al. Renal cell carcinoma risk associated with lower intake of micronutrients. Cancer Med **2018**, 7, 4087-4097.

63. Ben Fradj M.K., Ouanes Y., Hadj-Taeib S., Mrad D.K., Bibi M., Jmal K., Sanhaji H., Nouira Y., Feki M. Prognostic Significance of Plasma Folate and Cobalamin Concentrations in Non-Muscle-Invasive Bladder Cancer: A Prospective Cohort Study. Cancer Invest **2021**, 39, 240-250.

64. Cho E., Giovannucci E.L., Joh H.K. Nutrients related to one-carbon metabolism and risk of renal cell cancer. Cancer Causes Control **2013**, 24, 373-382.

65. Clasen J.L., Heath A.K., Scelo G., Muller D.C. Components of one-carbon metabolism and renal cell carcinoma: a systematic review and meta-analysis. Eur. J. Nutr. **2020**, 59, 3801-3813.

66. Boot I.W.A., Wesselius A., Yu E.Y.W., Brinkman M., van den Brandt P., Grant E.J., White E., Weiderpass E., Ferrari P., Schulze M.B. et al. Dietary B group vitamin intake and the bladder cancer risk: a pooled analysis of prospective cohort studies. Eur. J. Nutr. **2022**, 61, 2397-2416.

67. Bystrom P., Bjorkegren K., Larsson A., Johansson L., Berglund A. Serum vitamin B12 and folate status among patients with chemotherapy treatment for advanced colorectal cancer. Ups. J Med Sci. **2009**, 114, 160-164.

68. Al-Ghnaniem R., Peters J., Foresti R., Heaton N., Pufulete M. Methylation of estrogen receptor alpha and mutL homolog 1 in normal colonic mucosa: association with folate and vitamin B-12 status in subjects with and without colorectal neoplasia. Am. J. Clin. Nutr. **2007**, 86, 1064-1072.

69. Cheng T.Y., Makar K.W., Neuhouser M.L., Miller J.W., Song X., Brown E.C., Beresford S.A., Zheng Y., Poole E.M., Galbraith R.L. et al. Folate-mediated one-carbon metabolism genes and interactions with nutritional factors on colorectal cancer risk: Women's Health Initiative Observational Study. Cancer **2015**, 121, 3684-3691.

70. Dahlin A.M., Van G.B., Hultdin J., Johansson I., Hallmans G., Palmqvist R. Plasma vitamin B12 concentrations and the risk of colorectal cancer: a nested case-referent study. Int. J. Cancer **2008**, 122, 2057-2061.

71. Ghorbani M., Azghandi M., Khayami R., Baharara J., Kerachian M.A. Association of MTHFR C677T variant genotype with serum folate and Vit B12 in Iranian patients with colorectal cancer or adenomatous polyps. BMC. Med Genomics **2021**, 14, 246.

72. Eussen S.J., Vollset S.E., Hustad S., Midttun O., Meyer K., Fredriksen A., Ueland P.M., Jenab M., Slimani N., Boffetta P. et al. Plasma vitamins B2, B6, and B12, and related genetic variants as predictors of colorectal cancer risk. Cancer Epidemiol. Biomarkers Prev. **2010**, 19, 2549-2561.

73. Lee J.E., Li H., Giovannucci E., Lee I.M., Selhub J., Stampfer M., Ma J. Prospective study of plasma vitamin B6 and risk of colorectal cancer in men. Cancer Epidemiol. Biomarkers Prev. **2009**, 18, 1197-1202.

74. Chen F.P., Lin C.C., Chen T.H., Tsai M.C., Huang Y.C. Higher plasma homocysteine is associated with increased risk of developing colorectal polyps. Nutr. Cancer **2013**, 65, 195-201.

75. Liu G.J., Wang Y.J., Yue M., Zhao L.M., Guo Y.D., Liu Y.P., Yang H.C., Liu F., Zhang X., Zhi L.H. et al. High expression of TCN1 is a negative prognostic biomarker and can predict neoadjuvant chemosensitivity of colon cancer. Sci. Rep. **2020**, 10, 11951.

76. Zhu X., Yi K., Hou D., Huang H., Jiang X., Shi X., Xing C. Clinicopathological Analysis and Prognostic Assessment of Transcobalamin I (TCN1) in Patients with Colorectal Tumors. Med Sci. Monit. **2020**, 26, e923828.

77. Lo-Bisgaard T., Espelund U., Frystyk J., Rasmussen T.R., Nexo E., Arendt J.F.H. Vitamin B12 and its binding proteins in patients with non-small cell lung cancer referred to fast-track diagnostic work-up for lung cancer. Scand. J. Clin. Lab Invest **2020**, 80, 14-19.

78. Fanidi A., Carreras-Torres R., Larose T.L., Yuan J.M., Stevens V.L., Weinstein S.J., Albanes D., Prentice R., Pettinger M., Cai Q. et al. Is high vitamin B12 status a cause of lung cancer? Int. J. Cancer **2019**, 145, 1499-1503.

79. Tastekin D., Erturk K., Bozbey H.U., Olmuscelik O., Kiziltan H., Tuna S., Tas F. Plasma homocysteine, folate and vitamin B12 levels in patients with lung cancer. Exp Oncol. **2015**, 37, 218-222.

80. Brasky T.M., Ray R.M., Navarro S.L., Schenk J.M., Newton A.M., Neuhouser M.L. Supplemental one-carbon metabolism related B vitamins and lung cancer risk in the Women's Health Initiative. Int. J. Cancer **2020**, 147, 1374-1384.

81. Luu H.N., Wang R., Jin A., Koh W.P., Yuan J.M. The association between dietary vitamin B12 and lung cancer risk: findings from a prospective cohort study. Eur. J. Cancer Prev. **2021**, 30, 275-281.

82. Li H., Guo L., Cai Z. TCN1 is a potential prognostic biomarker and correlates with immune infiltrates in lung adenocarcinoma. World J. Surg. Oncol. **2022**, 20, 83.

83. Ilhan I.E., Sari N., Yesil S., Eren T., Tacyildiz N. Anthropometric and Biochemical Assessment of Nutritional Status in Pediatric Cancer Patients. Pediatr. Hematol. Oncol. **2015**, 32, 415-422.

84. Maritsi D.N., Vartzelis G., Dana H., Kosmidis H., Douna V., Kossiva L. Plasma Folate, Vitamin B12 and Homocysteine Levels in Children with Solid Tumors at Diagnosis; Results from a Pediatric Referral Centre. Indian J. Pediatr. **2016**, 83, 483-484.

85. Gorgulu O., Selcuk T., Ozdemir S., Sayar C., Beyazit Y., Akbas Y. Evaluation of the roles of serum vitamin B(12), folate and homocysteine levels in laryngeal squamous cell carcinoma. J. Int. Med Res. **2010**, 38, 2047-2052.

86. Kharb S., Kumar S., Kundu Z.S. Homocysteine, a biomarker of osteosarcoma. J. Cancer Res. Ther. **2015**, 11, 51-53.

87. Wu Y.H., Wu Y.C., Chu F.Y., Cheng S.J., Sun A., Chen H.M. Significantly higher frequencies of hematinic deficiencies and hyperhomocysteinemia in oral precancer patients. J. Formos. Med Assoc. **2019**, 118, 1299-1307.

88. Almadori G., Bussu F., Galli J., Cadoni G., Zappacosta B., Persichilli S., Minucci A., Giardina B., Maurizi M. Serum levels of folate, homocysteine, and vitamin B12 in head and neck squamous cell carcinoma and in laryngeal leukoplakia. Cancer **2005**, 103, 284-292.

89. Nacci A., Dallan I., Bruschini L., Traino A.C., Panicucci E., Bruschini P., Mancini V., Rognini F., Fattori B. Plasma homocysteine, folate, and vitamin B12 levels in patients with laryngeal cancer. Arch Otolaryngol. Head Neck Surg. **2008**, 134, 1328-1333.

90. Bo Y., Xu H., Zhang H., Zhang J., Wan Z., Zhao X., Yu Z. Intakes of Folate, Vitamin B6, and Vitamin B12 in Relation to All-Cause and Cause-Specific Mortality: A National Population-Based Cohort. Nutrients. **2022**, 14.

91. Wang Y., Yue C., Fang J., Gong L., Lian M., Wang R., Feng L., Ma H., Ma Z., Liu H. Transcobalamin I: a novel prognostic biomarker of neoadjuvant chemotherapy in locally advanced hypopharyngeal squamous cell cancers. Onco. Targets. Ther. **2018**, 11, 4253-4261.
