# Supplementary material for: High Plasma Vitamin B12 and Cancer in Human Studies: A Scoping Review to Judge Causality and Alternative Explanations
Source: Nutrients. 2022 Oct 25;14(21):4476. doi: 10.3390/nu14214476 (PMC9658086; doi:10.3390/nu14214476)
Supplement: Supplementary file 1 [file nutrients-14-04476-s001.zip › Tables S1 to S11_Vitamin B12 & Cancer.pdf]

High plasma vitamin B12 and cancer in human studies: a scoping review to judge causality and alternative explanations

Author: Rima Obeid

Supplemental Tables

Table S1. Search strings applied for PubMed

**Search #**	**Population –** Key words: patients with cancer, lymphoma, leukemia, blood cancers, solid cancers of any organ, tumors
#1 Population	Identical to #3
	**Intervention** – Key words: cyanocobalamin, cobalamin, hydroxocobalamin, vitamin B12 status, methylcobalamin, serum/plasma vitamin B12, plasma/serum holoTC, serum/plasma
#2 intervention	"Vitamin B12*"[tiab] OR cobalamin*[tiab] OR cyanocobalamin[tiab] OR "plasma cobalamin"[tiab] OR "serum cobalamin"[tiab] "Vitamin B12 intake"[tiab] OR "plasma vitamin B12"[tiab] OR "serum vitamin B12"[tiab] methylcobalamin[tiab] OR hydroxocobalamin[tiab] OR hydroxycobalamin[tiab] OR holotranscobalamin[tiab] OR "Vitamin B12 analogues"[tiab] OR transcobalamin[tiab] OR haptocorrin[tiab] OR "corrinoids"[tiab] OR "Vitamin B 12/blood"[Mesh] OR "Vitamin B 12/metabolism"[MAJR]
	**Comparison –** key words: no cancer, healthy controls, placebo or other non-B12 interventions
	**Outcome –** key words: cancer
#3 outcome	"cancer risk"[tiab] OR "cancer"[tiab] OR "tumor"[tiab] OR "Neoplasms/etiology"[MAJR] OR "Carcinoma/blood"[MAJR] OR "Neoplasms/blood"[Mesh]
#4	**Exclude animal studies**<br>Mouse[tiab] OR mice[tiab] OR rat[tiab] OR rats[tiab] OR murine[tiab] OR bacteria[tiab] OR "in vitro"[tiab]
Final Search	#2 AND #3 NOT #4
Additional filters	Publication date between 01.01.2005 and 06.03.2022 (**Search date:** 06.03.2022)

Search results: 238 articles qualified for title and abstract screening.

Table S2. Studies excluded in the full text stage and the reason for exclusion.		
#	PMID	Why excluded
1	33601391	B12 treatment in deficient patients with total gastrectomy for gastric cancer
2	31868326	Study design (oncologic patients with and without elevated plasma B12)
3	31802547	Prevalence of high B12, but no control group
4	31619709	No control group
5	31070145	did not report unit of measurements, not clear if the effect size was mean or median, no measure of data dispersity
6	26410155	letter to the editor
7	26746677	no B12 was shown or studied
8	25145486	study not showing appropriate analysis for the specific association between B12 and cervical neoplasia- it show only use of B12 as categorical factor and interaction with HPV
9	21612847	no data on B12
10	25157842	case only study, no controls
11	24500500	case only study no comparison or follow up
12	22729741	very likely double publication as 103 (large text overlap, very close sample size, no definition of recruitment time periods); results are principally the same as in 103
13	24485544	Letter to the editor, design not appropriate, although mostly reasons for high B12 are the same common one
14	24395112	prospective study of cases not including controls
15	24344032	low number of cases < 50 and no healthy control group (n = 6 of benign tumor were included)
16	17513884	only patients, no control group
17	16941173	B12 not presented/not analyzed
18	21111562	case report- showing that HC was indicative of disease progression
19	17474859	only patients no controls
20	20932223	not relevant
21	23029349	1)- possible overlap with the 2013 publication; 2)- Studied diseases associated with high B12 (not only cancer)
22	32895740	there is no control group to compare with
23	17449906	studied genotypes of TCN2 and others in relation to DNA methylation in patients with cancer

Table S3. Observational studies on plasma/serum B12 or B12 intake and **cancers of the esophagus and stomach**

Reference (PMID author year citation)	Exposure /outcome/population	High B12 vs. reference category	Effect size	Adjustments/confounders
33619628 Pan et al., 2021 [1]	B12 intake and esophageal squamous cell carcinoma. Squamous cell carcinoma, n = 100 patients and 100 controls<br>"China"	Reference: Q1 0.71-4.04 µg/d<br>Q2 (4.04–5.39) µg/d<br>Q3 (5.39–7.32) µg/d<br>Q4 7.32-22.38 µg/d	Median B12 intake in controls 5.38 (3.98–7.31) vs. in cases 5.40 (4.14–7.39) (ns)<br>OR (95%CI) =<br>1.44 (0.52–3.93)<br>1.41 (0.52–3.83)<br>1.66 (0.60–4.55)	adjusted for sex, age, BMI, education, annual income, smoking, alcohol drinking and consumptions of total vegetables, fruits and animal foods
	S-B12 concentrations, ng/L and esophageal squamous cell carcinoma	Reference: Q1; 98-303 ng/L<br>Q2 (302.75–401.50)<br>Q3 (401.50–526.50)<br>Q4 527-1202	Median B12 in controls 417.50 (334.00–535.75) vs. in cases 382.00 (257.25–506.25) ng/L, p = 0.026.<br>OR (95%CI) =<br>**0.37 (0.14–0.97)**<br>**0.31 (0.11–0.84)**<br>**0.30 (0.11–0.86)**	Same as above
	serum holoTC, pmol/L and esophageal squamous cell carcinoma	Reference: Q1; 49-73<br>Q2 (73.21–86.30)<br>Q3 (86.30–116.24)<br>Q4 116-675	Median holoTC in controls 101.75 (82.75–129.59) vs. in cases 77.85 (68.11–91.61) pmol/L, p <0.01.<br>OR (95%CI) =<br>0.85 (0.29–2.49)<br>**0.22 (0.08–0.64)**<br>**0.19 (0.07–0.51)**	Same as above
25607998 Chang et al., 2015 [2]	Plasma B12, pmol/L and esophageal cancer. Esophagus, n= 218 patients with esophageal cancer and 415 controls	Reference Q1 < 154 pmol/L;<br>Q2 (154-229)<br>Q3 (229-324)<br>Q4 (>324)	OR (95%CI) =<br>0.97 (0.49-1.90)<br>**2.16 (1.15-4.04)**<br>**2.80 (1.51-5.18)**	age, sex, BMI, education, smoking, H. Pylori (in stomach cancer), Hepatitis B infection and aflatoxin (in liver cancer), other micronutrients

	"China"			
	Plasma B12, pmol/L and stomach cancer (N = 206 stomach cancer)	Reference Q1 < 154 pmol/L<br>Q2 (154-229)<br>Q3 (229-324)<br>Q4 (>324)	OR (95%CI) =<br>0.90 (0.49-1.68)<br>1.09 (0.60-2.00)<br>**2.17 (1.21-3.89)**	Same as above
27748414 Ren et al, 2016 [3]	S-B12 and esophageal cancer. n = 498 OSCC, n =947 controls<br>"China"	quartiles of B12 concentrations weighted by the entire NIT cohort; not further specified in pmol/L (reference group is lowest quartile)	HR (95%CI) = all not adjust.<br>Q2 vs. Q1 = 0.79 (0.59-1.05)<br>Q3 vs. Q1 0.84 (0.62-1.12)<br>Q4 vs. Q1 0.84 (0.62-1.13)	Not adjusted
	S-B12 and Gastric cancer. Gastric cancer, n=255 GCA, n=947 controls	(reference group is lowest quartile)	HR (95%CI) = all not adjust.<br>Q2 0.75 (0.52-1.09)<br>Q3 0.75 (0.51-1.11)<br>Q4 0.95 (0.65-1.39)	Not adjusted
28568053 Miranti et al., 2017 [4]	S-B12 and upper gastrointestinal tract cancer: 127 cases of non-cardiagastric adeno-carcinoma (NCGA); 46 cases of esophago-gastric junctional adeno-carcinoma (EGJA); 60 of esophagal squamous cell carcinoma (ESCC) and 326 controls, "Finland"	Quartiles of S-B12 (based on the controls), pmol/L<br>Q1 < 291<br>Q2 291-349<br>Q3 349-438<br>Q4 > 438 **(reference**)	OR (95%CI)<br>non-cardiagastric adenocarcinoma<br>Q1 **5.77 (2.65-12.56**)<br>Q2 **3.38 (1.50-7.62**)<br>Q3 **5.25 (1.46-7.20)**<br>Esophago-gastric junctional adenocarcinoma<br>Q1 0.90 (0.35-2.34)<br>Q2 0.88 (0.34-2.31)<br>Q3 0.57 (0.19-1.72)<br>Esophagal squamous cell carcinoma<br>Q1 1.43 (0.62-3.27)	all analyses adjusted for several covariates: age, BMI, nr cigarettes, n years of smoking, education, alcohol, energy intake, fruits and vegetables intake

			Q2 1.22 (0.51-2.90)<br>Q3 1.00 (0.42,2.41)	
18006931 Vollset et al., 2007 [5]	P-B12 and gastric cancers. 247 gastric cancers and 631 controls<br>"Europe, Epic cohort"	Quartiles of P-B12<br>Lowest Q1 is the reference	OR (95%CI) =<br>Q2 vs. Q1 0.91 (0.60-1.40)<br>Q3 vs. Q1 **0.60 (0.38-0.96)**<br>Q4 vs. Q1 0.66 (0.40-1.07)	Adjusted for H. pylori status, smoking, and energy in conditional logistic model with stratification on matched sets.
	P-MMA and gastric cancer	Quartiles of P-MMA<br>Lowest Q1 is the reference	Q2 vs. Q1 0.90 (0.56-1.45)<br>Q3 vs. Q1 0.81 (0.50-1.31)<br>Q4 vs. Q1 1.35 (0.84-2.17)	
22185224 Jessri, 2011 [6]	B12 intake and esophagal squamous cell carcinoma. s hospital-based case-control study, 47 cases with incident ESCC and 96 controls<br>"Iran"	B12 intake into tertiles, cut off not further specified	OR (95%CI) =<br>T2 vs. T1 0.87 (0.10-2.61)<br>T3 vs. T1 1.33 (0.60-3.03)	Adjusted for age, sex, BMI, gastrointestinal reflux, smoking history, physical activity, and education. However, cases consumed significantly more hot foods and beverages and fried and barbecued meals, compared to the controls
24132576 Sharp et al., 2013 [7]	B12 intake and esophageal adenocarcinoma, reflux esophagitis, and Barrett's esophagus. A total of 256 controls and 223 Esophageal adenocarcinoma, 220 reflux esophagitis, and 219 Barrett's esophagus cases	Quartiles of B12 intake, µg/d<br>Q1: ≤ (reference)<br>Q2 6.5-7.8<br>Q3 7.9-9.6<br>Q4 ≥ 9.7	Vitamin B12 intake, µg/d = 7.7 (2.2) in the controls; 8.9 (3.0) in cancer; 8.1 (2.7) in Reflux patients; 7.9 (2.5) in Barrett esophagus p <0.01.<br><u>Multivariate ORs (95% Cis):</u><br>Esophageal adenocarcinoma;<br>Q2 vs. Q1 1.38 (0.76, 2.51)<br>Q3 vs. Q1 1.66 (0.92, 2.99)<br>Q4 vs. Q1 **3.94 (2.17, 7.14**).<br>Barrett's esophagus:<br>Q2 vs. Q1 0.94 (0.50, 1.74)<br>Q3 vs. Q1 1.21 (0.66, 2.22)	ORs adjusted for age, sex and total energy, social class, waist/hip ratio, history of hernia, and history of gallstones

			Q4 vs. Q1 **2.11 (1.12, 3.98)**<br>reflux esophagitis:<br>Q2 vs. Q1 0.67 (0.38, 1.18)<br>**Q3 vs. Q1 0.49 (0.27, 0.89)**<br>Q4 vs. Q1 0.98 (0.53, 1.81)	
21178085 Ibiebele et al., 2011 [8]	B12 intake in 2 population based case control studies in Australia on patients with Barrett's esophagus patients without (n= 266) or with (n= 101) dysplasia were compared with population controls (n = 577); similarly, esophageal adenocarcinoma (n = 636) or esophageal squamous cell carcinoma (n = 245) patients were compared with population controls (n = 1507)	B12 intake, μg/d<br>Q1 0.8 (0.0–1.1) Reference<br>Q2 1.3 (1.1–1.5)<br>Q3 1.8 (1.5–2.1)<br>Q4 2.5 (2.1, 7.8)	OR (95% CI)<br>Barrett's esophagus no dysplasia<br>Q2 vs. Q1 1.19 (0.72–1.98)<br>Q3 vs. Q1 0.85 (0.50–1.42)<br>Q4 vs. Q1 1.41 (0.86–2.32)<br>Barrett's esophagus + dysplasia<br>Q2 vs. Q1 0.67 (0.28–1.59)<br>Q3 vs. Q1 1.08 (0.48–2.47)<br>Q4 vs. Q1 1.66 (0.75–3.70)<br>esophageal adenocarcinoma<br>Q2 vs. Q1 0.81 (0.59–1.09)<br>Q3 vs. Q1 0.87 (0.64–1.17)<br>Q4 vs. Q1 0.96 (0.71–1.30)<br>esophageal squamous cell carcinoma<br>Q2 vs. Q1 0.79 (0.51–1.23)<br>Q3 vs. Q1 0.78 (0.50–1.22)<br>Q4 vs. Q1 0.89 (0.58–1.32)	Multivariable adjustment for age, gender, education, BMI 1 y previously, frequency of heartburn or acid reflux 10 y prior to diagnosis, lifetime alcohol intake, pack-years of smoking, NSAID use, and total energy intake
24481406 Xiao, 2014 [9]	B12 intake and gastric and esophageal cancer. Self-reported intakes of folate, methionine, vitamin	B12 intake in quintiles<br>Q1 median intake 2.5 μg/d<br>Q2 median intake 3.6 μg/d	RR (95%CI) Esophageal squamous cell carcinoma<br>Q1 vs. Q3 1.21 (0.78, 1.87)<br>Q2 vs. Q3 1.06 (0.67, 1.68)<br>Q4 vs. Q3 0.96 (0.59, 1.56)	Adjusted for age at baseline (continuous); sex; race/ethnicity; education; marital status; health status; body mass index; smoking status; smoking dose;

	B6, and vitamin B12 and gastric and esophageal cancer in 492 293 men and women	Q3 median intake 4.4 µg/d (Reference)<br>Q4 median intake 5.4 µg/d<br>Q5 median intake 7.3 µg/d	Q5 vs. Q3 0.85 (0.52, 1.41)<br>Esophageal adenocarcinoma<br>Q1 vs. Q3 1.02 (0.78, 1.34)<br>Q2 vs. Q3 1.04 (0.80, 1.34)<br>Q4 vs. Q3 1.08 (0.84, 1.39)<br>Q5 vs. Q3 1.04 (0.80, 1.34)<br>Gastric cardia- adenocarcinoma<br>Q1 vs. Q3 0.99 (0.72, 1.37)<br>Q2 vs. Q31.12 (0.83, 1.51)<br>Q4 vs. Q3 1.02 (0.76, 1.39)<br>Q5 vs. Q3 1.09 (0.81, 1.47)<br>Non-cardia gastric adenocarcinoma<br>Q1 vs. Q3 1.10 (0.83, 1.47)<br>Q2 vs. Q3 1.32 (1.00, 1.73)<br>Q4 vs. Q31.06 (0.79, 1.41)<br>Q5 vs. Q3 1.27 (0.96, 1.68)	time since quitting; physical activity; alcohol; multivitamin use; family history of any cancer; and total caloric intake
Mayne et al., 2001 [10]<br>Added from the Meta-analysis PMID 11588131	S-B12 and esophageal adenocarcinoma, gastric cardia adenocarcinoma, esophageal squamous cell carcinoma, noncardia gastric cancer.	Upper 75th vs. lowest 25th percentiles (reference group) in the individual cohorts, percentile cutoffs not further specified	OR (95%CI)<br>esophageal adenocarcinoma **1.39 (1.10–1.76)**<br>Gastric cardia adenocarcinoma **1.27 (1.01–1.60)**<br>Esophageal squamous cell carcinoma **1.51 (1.15–2.00)**<br>Noncardia gastric cancer **1.38 (1.13–1.68)**	Adjusted for sex; site; age; race; proxy status; income; education; BMI; cigarettes/d; years of consuming alcohol, energy intake
34470601<br>Khairan 2021 [11] (cohort study)	87,053 Japanese individuals who completed a food frequency questionnaire and	energy-adjusted B12 intake Quintiles<br>Q1 3.7 ± 2.6 (Ref.)<br>Q2 7.4 ± 0.6<br>Q3 9.3 ± 0.6	Hazard ratios (HRs) and 95%CI<br>1.13 (0.80–1.59)<br>1.43 (0.99–2.06)<br>1.58 (1.07–2.34)<br>1.75 (1.13–2.71)	Adjusted for age, sex, public health center area, body mass index, smoking, alcohol consumption, family history of cancer, and physical activity in

	were followed up from 1995-1998 to 2013 and 2015 esophageal cancer	Q4 11.6 ± 0.8<br>Q5 18.3 ± 6.7 μg/d	Subgroup analysis showed that the associations in Q4+Q5 were significant only for never drinkers, but there was no dose-response associations<br>1.45 (0.56–3.75)<br>2.31 (0.96–5.53)<br>3.27 (1.39–7.66)<br>2.82 (1.18–6.74)	METs. And mutually adjusted further for vitamin B6, folate, and methionine
26724465<br>Arendt et al., 2016 [12]<br>(cohort study)	S-B12 (1 yr pre-diagnosis) and mortality from gastric cancer (n = 497 gastric cancers), Health register data "Denmark"	S-B12, pmol/L<br>< 200 excluded<br>200-600 (Reference group)<br>601-800<br>> 800	Cancer mortality risk ratio 30 days<br>S-B12 601-800 vs. 200-600 pmol/L =1.5 (0.6–3.7)<br>S-B12 > 800 vs. 200-600 pmol/L = 2.4 (1.1–5.0).<br>Longer term mortality 31-90d, p value =0.07<br>91-365d mortality p value = 0.90	Adjusted for age, sex, calendar year, Charlson comorbidity score index, and cancer stage. Results do not support causality (only 30d mortality was high). Possible bias: excluded all values < 200 pmol/L, confounding by indication, and confounding by underreported supplements

PMID: 29954131<br>Qiang et al.,2018 [13]<br>(Meta-analysis)	Systematic review and meta-analysis on B12 intake and esophageal cancer	Vitamin B12 intake from 7 studies (PMIDs): 24481406, 22185224, 24132576, 21178085, 11588131 3562297, Oral. Oncol. 1999, 35, 22–26<br><br>Jessri et al., 2011*<br>Sharp et al, 2013*<br>Stefani et al., 1999<br>Xiao et al., 2014*<br>Tuyns et al., 1987<br>Ibiebele et al., 2011*<br>Mayne et al., 2001<br><br>* Included in our search	a positive correlation between B12 intake and the risk of esophageal cancer OR and 95%CI = 1.30 (1.05–1.62).<br>A dose-response analysis revealed a positive linear association between dietary B12 intake and cancer risk (p = 0.192).<br>Each 1 μg/day increase in dietary B12 intake increases the risk of esophageal cancer by 2% (OR = 1.02 (1.00–1.03)<br>Estimate (OR and 95%CI) of the individual studies included:<br>1,33 (0,59, 2,99)<br>3,94 (2,17, 7,15)<br>1,30 (0,89, 1,89)<br>0,53 (0,29, 0,96)<br>1,26 (1,01, 1,58)<br>1,77 (1,28, 2,45)<br>0,96 (0,71, 1,30)<br>0,89 (0,59, 1,34)<br>1,39 (1,10, 1,76)<br>1,51 (1,15, 1,99)<br>**1.30 (1.05, 1.62) = pooled effect size**	

35538710 He et al., 2022 [14] (meta-analysis)	Gastric cancer		B_{12} intake and the risk of gastric cancer<br>(OR = 0.88, 95% CI: 0.69-1.12).<br>B_{12} intake and gastric cancer in *Helicobacter pylori* (*Hp*)-negative people<br>(OR = 0.83, 95% CI: 0.62-0.99)<br>B_{12} intake and gastric cancer in *Helicobacter pylori* (*Hp+) positive* people<br>(OR = 1.66, 95% CI: 1.27-2.16)<br>vitamin B_{12} might increase the risk of non-cardia gastric cancer<br>(OR = 1.15, 95% CI: 1.01-1.33).<br>vitamin B_{12} intake and gastric cancer risk in nonsmokers (OR = 0.83, 95% CI: 0.71-0.96).<br>in smokers (OR = 1.08, 95% CI: 0.71-1.47).	

Table S4. Observational studies on plasma/serum B12 or B12 intake and **cancers of the pancreas**				
Reference (PMID author year citation)	Exposure /outcome	High B12 vs. reference category	Effect size	Adjustments/confounders
24590454 Jansen 2014 [15]	B12 intake and pancreas cancer. case-control study 384 cases with pancreas cancer and 983 controls frequency matched on recruitment age, race, sex, and residence area	Quintiles of B12 intake, µg per 1000 kcal<br>Q1 1.72 (0.53–2.05) = reference category<br>Q2 2.3 (2.05–2.49)<br>Q3 2.7 (2.49–2.91)<br>Q4 3.17 (2.91–3.53)<br>Q5 4.11 (3.53–12.88)	OR (95%CI)<br><br>Q2 vs. Q1 0.94 (0.65,1.36)<br>Q3 vs. Q1 0.62 (0.42,0.93)<br>Q4 vs. Q1 0.85 (0.58,1.24)<br>Q5 vs. Q1 0.72 (0.48,1.06)	odds ratios (OR) (95% CI) were adjusted for age, sex, cigarette smoking, body mass index, and diabetes<br>mellitus
21411310 Chuang et al., 2011 [16]	S-B12 and pancreas cancer. 463 cases and 464 controls Europe, nested CC study within the EPIC cohort	Quintiles of S- B12, pmol/L:<br>Q1 ≤267.3 (reference group)<br>Q2 267.3-330<br>Q3 330-391.7<br>Q4 391.7-493.6<br>Q5 > 493.6	OR (95%CI)<br>for Q2 vs. Q1 = 0.9 (0.6-1.4)<br>Q3 vs. Q1 = 0.9 (0.5-1.3)<br>Q4 vs. Q1 = 1.0 (0.7-1.7)<br>Q5 vs. Q1 = 0.9 (0.6-1.5)	The groups age matched for (age at blood collection, sex, center, date and time of blood collection, fasting status) and further adjusted for education, smoking, plasma cotinine, alcohol, BMI, diabetes
17545639 Schernhammer et al., 2007 [17]	S-B12 and B12 intake in relation to pancreas cancer. 208 cases, 623 controls from 4 cohorts; The Nurse Health Study; the Health Professionals Follow up study; the Physician	S-B12 and B12 intake (total intake and food B12 intake) were measured.<br>Quartiles of vitamin B12 in the controls of the specific cohorts (cut offs not defined).<br>Lowest quartile is the reference group	Mean (SD) of total B12 intake µg/d from foods and suppl. = 14.8 (42.3) in patients and 16.4 (45.0) in the controls. The mean (SD) of intake from only foods were 6.5 (3.6) in patients and 7.1 (4.5) in the controls (p values 0.9 and 0.2).<br>In the whole cohort incl. suppl users OR (95%CI)	the matching factors were [year of birth, smoking, fasting, month of blood draw] and cohort, BMI, physical activity, and a history of diabetes; and tHcy, folate, and B6

	Health Study; the Women Health Initiative		for Q2 vs. Q1 = 1.23 (0.76–1.99)<br>Q3 vs. Q1 = 1.40 (0.87–2.28)<br>Q4 vs. Q1 = 0.86 (0.50–1.46)<br>In the subcohort without suppl. users OR (95%CI) for Q2 vs. Q1 1.58 (0.77–3.24)<br>Q3 vs. Q1 1.23 (0.58–2.57)<br>Q4 vs. Q1 1.04 (0.49–2.19)	
19415507<br>Gong et al., 2009 [18]	Intake of B12 from foods and supplements and pancreatic cancer. 532 cases with pancreatic cancer and 1701 controls "USA"	Quartiles of B12 intake from food plus supplements, µg/d:<br>Q1 (<4.2)= reference category<br>Q2 (4.2-<6.4)<br>Q3 (6.4-<10.1)<br>Q4 (10.1-<14.5)<br>Q5 (≥14.5)<br>Quartiles of B12 intake from food, µg/d<br>Q1 (<3.4) = reference<br>Q2 (3.4-<4.4)<br>Q3 (4.4-<5.6)<br>Q4 (5.6-<7.5)<br>Q5 (≥7.5)	OR (95%CI) fully adjusted<br>Q2 vs. Q1 = 1.4 (0.97-1.9)<br>Q3 vs. Q1 =1.4 (0.99-1.9)<br>Q4 vs. Q1 =1.3 (0.93-1.8)<br>Q5 vs. Q1 =1.3 (0.92-1.8).<br><br>For intake from foods:<br>Q2 vs. Q1 = 1.3 (0.92-1.9)<br>Q3 vs. Q1 **1.8 (1.3 -2.5)**<br>**Q4 vs. Q1 1.7 (1.2 -2.5)**<br>**Q5 vs. Q1 1.9 (1.3 -2.6)**	Adjusted for age (in 5-year group), sex, and total energy intake (quartiles) and additionally adjusted for body mass index, history of diabetes, smoking, and alcohol consumption.
10088624<br>Stolzenberg-Solomon, 1999 [19]	Serum B12 and pancreatic cancer. Nested case control study, of male Finnish smokers; 126 with pancreatic	Serum vitamin B12 tertiles, pg/ml:<br>T1 <=427<br>T2 427 -550<br>T3 >550	OR (95%CI) =<br>T2 vs. T1 0.89 (0.51–1.55)<br>T3 vs. T1 1.26 (0.73–2.17)<br>Interaction with smoking (higher risk at high B12 in men who smoked < 20 ciga.	matched by date of baseline blood draw study center, age, trial intervention group, and completion of dietary history and adjusted for the matching variables (age, month of blood draw, completion of

	cancer matched to 247 controls		per day). But the associations were not consistent in heavy smokers.	dietary questionnaire, study center, and intervention group), and for serum folate.
29904184 Marley et al., 2018 [20]	B12 intake and pancreas cancer. Case-controlled study USA 150 cases with pancreas cancer and 459 controls	B12 intake from foods [median intake in the quartiles, µg/d 2.7, 4.4, 6.6, and 12.2]<br>Total B12 intake (food and supplements) [Median (µg/day) 3.6, 6.2, 10.0, and 17.8].<br>The lowest quartile was the reference category.<br>The intake ranges among the quartiles were not reported.	Cases and controls did not differ in dietary or total B12: Dietary intake (µg/d). Mean SD = 7.2 (4.9) vs. 7.0 (6.7); Total intake = 12.3 (15.8) vs. 11.0 (13.5).<br>Fully adjusted OR (95%CI) = For food B12 quartiles: Q2 vs. Q1 1.38 (0.74–2.59)<br>Q3 vs. Q1 1.62 (0.83–3.19)<br>Q4 vs. Q1 2.03 (0.98–4.26).<br>For total B12 intake Q2 vs. Q1 1.18 (0.65–2.16)<br>Q3 vs. Q1 0.90 (0.46–1.73)<br>Q4 vs. Q1 1.66 (0.89–3.15).	Adjusted for age, sex, race, education, cigarette smoking, alcohol consumption, and total physical activity; and additionally for intake of energy, total fat, fiber, vegetables, and fruits
26724465 Arendt et al., 2016 [12] (**Cohort study**)	serum B12 and mortality after diagnosing pancreas cancer. 835 pancreas cancer identified in health register with serum B12 measurement up to one year prior to diagnosis	S-B12 measured up to 1 year prior to diagnosis Reference group B12 200-600 pmol/L (Reference)<br>601-800<br>> 800	30 days mortality risk ratio B12 601-800 vs. 200-600 pmol/L 1.4 (0.9–2.1); plasma B12 > 800 pmol/L vs. 200-600 pmol/L = **1.7 (1.2–2.5).**<br>For 31-90d mortality, p value =0.93; 91-365d mortality p = 0.68	Adjusted for age, sex, calendar year, Charlson comorbidity score index, and cancer stage.<br>Possible selection bias (excluded all values < 200 pmol/L), confounding by indication, and confounding by underreported supplement usage)
26711329 Huang et al.,	B12 intake and incident pancreatic	Q1 median intake 1.43 µg/d (reference)	HR (95%CI)<br>Q2 vs. Q1 0.97 (0.69–1.35)	Adjusted for age, sex, year of interview, dialect group, education,

2016 [21] (**Cohort study**)	cancer. 63,257 men and women, 271 incident pancreatic cancer cases after 16.3 years follow up “China”	Q2 2.05 µg/d<br>Q3 2.52 µg/d<br>Q4 3.26 µg/d	Q3 vs. Q1 0.99 (0.70–1.38)<br>Q4 vs. Q1 0.88 (0.62–1.24)	BMI
11282796 Stolzenberg-Solomon, 2001 [22] (Cohort study)	Energy adjusted B12 intake and pancreatic cancer. male smokers completed a self-administered dietary questionnaire at baseline, 157 developed pancreatic cancer during up to 13 years of follow-up from 1985 to 1997	Quintiles of energy-adjusted dietary B12 intake in µg/d<br>Q1 ≤7.57 (reference)<br>Q2 (7.58–9.26)<br>Q3 (9.27–11.08)<br>Q4 (11.09–13.68)<br>Q5 >13.68	HR (95%CI)<br>Q2 0.73 (0.44, 1.22)<br>Q3 1.21 (0.77, 1.90)<br>Q4 0.93 (0.57, 1.53)<br>Q5 0.88 (0.53, 1.48)	Adjusted for age, intervention - tocopherol and beta-carotene supplement), and energy-adjusted folate
33012287 Wei et al., 2020 [23] (Meta-analysis)	6 studies on B12 intakes; and 3 on B12 concentrations and pancreas cancer	Six studies reported results on vitamin B12 intake [PMID: 19415507, 26711329, 11282796, 24590454, 29904184, and three studies reported blood vitamin B12 concentrations [10088624, 17545639, 24249744]	The summary RRs(95%CI) (random-effects models); 0.97 (0.78–1.16) for vitamin B12 intake<br>1.17 (0.64–1.70) for blood concentrations (both not significant)	

Table S5. Observational studies on plasma/serum B12 or B12 intake and **breast and cervical cancers**. All studies are case-control studies, except when otherwise indicated.				
Reference (PMID author year citation)	Exposure /outcome	High B12 vs. reference category	Effect size	Adjustments/confounders
30758268 [24]	B12 intake and breast cancer. 151 with breast cancer and 154 controls "Iran"	Quartiles of B12 intake from foods, µg/d<br>Q1 (≤2.86) -reference group-<br>Q2 (2.87-3.97)<br>Q3 (3.98-5.10)<br>Q4 (≥5.11) µg/d.<br>Quartiles of B12 intake from diet plus suppl.<br>Q1 (≤2.88)<br>Q2 (2.89-4.17)<br>Q3 (4.18-5.46)<br>Q4 (≥5.47) µg/d	OR (95% CI) all adjusted<br>For dietary B12 intake:<br>Q2 vs. Q1 = 0.48 (0.24-0.99)<br>Q3 vs. Q1 = 0.42 (0.20-0.86)<br>Q4 vs. Q1 = 0.31 (0.15-0.65)<br><br>For B12 intake from foods and suppl.<br>Q2 vs. Q1 = 0.48 (0.22-0.98)<br>Q3 vs. Q1 = 0.42 (0.20-0.86)<br>Q4 vs. Q1 = 0.20 (0.09-0.43)	Adjusted for education, physical activity, parity, occupation, total energy intake. Same associations tested for subgroup of estrogen receptor (+/-), progesterone receptor (+/-), and HER-2 ; generally similar results were obtained (some less significant due to low sample size)
27121532 Agnoli et al., 2016 [25]	S-B12 and breast cancer. 276 cases and 276 controls "Europe, nested C-C study within EPIC cohort"	Quartiles of S-B12 concentrations in the controls:<br>Q1 175-443 (reference group)<br>Q2 443-545<br>Q3 546-688<br>Q4 688-2310 pg/ml	RR (95%CI) for breast cancer<br>Q2 vs. Q1 = 0.67 (0.40, 1.11)<br>Q3 vs. Q1 = 0.69 (0.42, 1.15)<br>Q4 vs. Q1 = 0.88 (0.53, 1.45)	Matched for age, menopausal status, date at recruitment, micronutrients analysis in the same batch.<br>Adjustment for age, menopausal status, recruitment date, distance between hormones and diet, family history, age at menarche, oral contraceptive use, smoking status,

				education, alcohol and BMI
27905104 Matejcic et al., 2017 [26]	Plasma B12 and breast cancer. 2941 cases and 2521 controls "Europe, nested C-C study within EPIC cohort"	Quartiles of plasma B12<br>Q1(<293.6) the reference group<br>Q2(293.6-373.1)<br>Q3(373.1-460.0)<br>Q4(> 460) pmol/L	OR (95%CI) for<br>Q2 vs. Q1 = 1.00 (0.85-1.19)<br>Q3 vs. Q1 = 0.95 (0.80-1.13)<br>Q4 vs. Q1 = 1.14 (0.95-1.36)	Matched for age at blood donation, center, hormone therapy, menopausal status, fasting status. Adjusted for date of blood collection, education, BMI, height, physical activity, use of hormone replacement drugs, alcohol intake, parity, menopausal state at recruitment
18326613 Lin et al., 2008 [27]	P-B12 concentrations and B12 intake and breast cancer. 850 patients and 850 controls "USA, nested within the Women health study"	Measured p- B12 and B12 intake (from foods; and foods and supplements).<br>Quintiles of P-B12 concentrations in the controls<br>Q1 ≤337 (reference group)<br>Q2 338-414; Q3 415-512<br>Q4 513-686<br>Q5 >686 pg/ml.<br>Quintiles of total B12 intake (foods and suppl.)<br>Q1 ≤4.3 (reference group)<br>Q2 4.4-5.5; Q3 5.6-7.2<br>Q4 7.3-10.5<br>Q5 >10.5 μg/d.	RR (95%CI) for p- B12 concentrations<br>Q2 vs. Q1 = 1.12 (0.80-1.56)<br>Q3 vs. Q1 **1.45 (1.03-2.04)**<br>Q4 vs. Q1 1.23 (0.87-1.72)<br>Q5 vs. Q1 1.29 (0.92-1.82).<br>For total B12 intake<br>Q2 vs. Q1 = 0.99(0.69-1.42)<br>Q3 vs. Q1 = 1.2(0.85-1.7)<br>Q4 vs. Q1 = 0.97(0.68-1.38)<br>Q5 vs. Q1 = **1.44 (1.02-2.04)**.<br>For food B12 intake<br>Q2 vs. Q1 0.66 (0.39-1.10)<br>Q3 vs. Q1= 0.78 (0.46-1.33)<br>Q4 vs. Q1 = 1.19 (0.73-1.94)<br>Q5 vs. Q1 = 0.88 (0.54-1.44).	Matching for age (up to 5 y), ethnicity, menopausal status, fasting status, mo and yr of blood return, postmenopausal hormone use and trial randomization year. Adjustments for matching variables, randomized treatment assignment, BMI, family history, history of benign breast disease, smoking, physical activity, alcohol, age at menarche, age at menopause, parity, and

		Quintiles of food B12 intake in the controls:<br>Q1 ≤3.8 (reference group)<br>Q2 3.9-4.8<br>Q3 4.9-5.6<br>Q4 5.7-7.1<br>Q5 >7.1 µg/d.	B12 total intake did not differ between patients and controls; 8.6 (9) vs.8.3 (10) µg/d; P-B12 did not differ between patients and controls median (5,95th) = 467 (254-1039) vs. 460 (258-1044) pg/ml.	age at first birth.<br>The sporadic associations could be rather due to chance.
30346061 Houghton et al. 2019 [28]	Plasma B12 and breast cancer. 1874 incident breast cancer with complete blood concentrations and similar n of controls (367 had 2 blood measurements) Nested case control in the prospective Nurses Health Study (NHS) analyzed separately for 1990 blood collection and 2000 blood collection	Quintiles of P-B12, pg/ml<br>Q1 <311 (Reference)<br>Q2 311 to 403<br>Q3 404 to 497<br>Q4 498 to 618<br>Q5 ≥619 pg/ml	RR (95%CI) for<br>Q2 vs. Q1 = 1.09 (0.88–1.35)<br>Q3 vs. Q1 = 1.11 (0.91–1.37)<br>Q4 vs. Q1 = 0.92 (0.75–1.15)<br>Q5 vs. Q1 = 1.06 (0.85–1.31).	matched for year of birth, time of blood collection, fasting status, month of blood draw, menopausal status at blood collection and diagnosis, taking postmenopausal hormones in the last 3 months
19389261 Ma et al., 2009<br><br>[29]	B12 intake and breast cancer. 458 age-matched pairs of Brazilian women with and without breast cancer	B12 intake and polymorphisms<br><br>B12 intake T1 < 3.9 µg/d (reference group)<br>T2 3.9-<7.3 µg/d<br>T3 ≥ 7.3 µg/d	Vitamin B12 intake, µg/day 7.5 ± 8.7 in patients and 7.0 ± 5.7 µg/d in the controls p = 0.260.<br>OR (95%CI) for<br>T2 3.9-<7.3 µg/d vs. < 3.9 µg/d = 0.90 (0.64-1.25)<br>T3 ≥ 7.3 µg/d vs. < 3.9 µg/d = 0.90 (0.65-1.26).	Age matched. Adjusted for age group, ethnicity, smoking status, alcohol consumption, moderate physical activity in the preceding 5 years, and number of live births

19838916 Ma et al., 2009 [30] (only abstract is available to us)	B12 intake and MTR gene polymorphism and breast cancer. Case-control study in Nagano Prefecture, Japan, in 388 pairs of patients with invasive breast cancer and age- and area-matched controls	B12 intake<br>Polymorphisms MTR gene	No association between B12 intake and cancer; no association between MTR genotype and breast cancer	
**20030812**<br>Maruti 2009 [31]	Dietary and total b12 intake and breast cancer. 318 incident breast cancer cases and<br>647 participating in a nested<br>case-control study of postmenopausal women within the VITamins And Lifestyle (VITAL) cohort	Dietary vitamin B2 (µg/day)<br>≥ 1.81 ((CC and high intake is the reference group)<br><1.81µg/d.<br>Total B12 intake<br>≥ 2.70 µg/d (CC and high intake is the reference group)<br>< 2.7 µg/d	Reference group are MTHFR CC genotype with high B12 intake.<br>OR (95%CI) =<br>CC with low intake 0.85 (0.52-1.37)<br>CT with low intake 1.03 (0.63-1.68)<br>TT with low intake **2.53 (1.25-5.12)**.<br>For low total B12 intake =<br>CC with low intake 1.02 (0.64-1.63)<br>CT with low intake 1.23 (0.76-1.98)<br>TT with low intake **2.73 (1.40-5.33)**	age- and race-matched controls and adjusted for age, race, family history of breast cancer, mammography within 2 years preceding baseline, history of breast biopsy, age at menarche, age at first birth, age at menopause, years of combined estrogen and progestin hormones, height, BMI, physical activity, energy intake, and for non-alcohol exposures, past-year alcohol intake. Energy was added to the models for all dietary and total nutrient exposures.

23408942 Yang 2013 [32]	Energy adjusted total B12 intake and breast cancer. Primary breast cancer cases (n = 2,325) and n = 2,525 controls. The 4-Corners Breast Cancer Study, 1999–2004	Energy-adjusted total B12 intakes<br>Q1 ≤5.32 (reference)<br>Q2 5.32–9.78<br>Q3 >9.78–13.98<br>Q4 >13.98 µg/d	OR (95% CI) =<br>Q2 vs. Q1 0.75 (0.61-0.93)<br>Q3 vs. Q1 0.83 (0.62-1.11)<br>Q4 vs. Q1 0.73 (0.53-1.00)	Cases and controls were frequency-matched by ethnicity and age (±5 years). Models adjusted for age, center, ethnicity, education, body mass index, total MET hours per week, total energy intake per day, total daily fiber intake, cigarette status, alcohol intake, parity, family history, oral contraceptive use and menopausal status, and intakes of other nutrients (folate, B6, methionine, B2)
**21705842 Chou 2011 [33]**	B12 intake and breast cancer. 391 breast cancer cases and 782 control subjects enrolled at the Tri-Service General Hospital in Taipei, Taiwan	B12 intake based on the distribution in the controls.<br>T1 ≤5.28 (Reference)<br>T2 5.29–8.15<br>T3 >8.15 µg/d	OR (95%CI)<br>T2 vs. T1 0.89 (0.53–1.65)<br>T3 vs. T1 0.83 (0.73–2.54)	matching variables of date of enrollment and fasting status, and further adjusted for age at enrollment, age at menarche, age at first full-term pregnancy, parity, menopausal status, age at menopause, postmenopausal hormone use, family history of breast cancer, supplement use, total energy intake.

35495919 Xu et al., 2022 [34] (Cohort study)	Ovarian Cancer Survival Cohort Study in 635 newly diagnosed OC patients aged 18–79 y, median follow-up of 37.2 months, China	Tertiles of energy adjusted B12 intake μg/d (Adjusted for energy by the residual method)<br><0.05<br>0.05–0.14<br>> 0.14	hazard ratio (HR) and 95% confidence intervals (CIs)<br>1.00 (Ref)<br>1.42 (0.86–2.35)<br>1.02 (0.63–1.65)<br>As a continuous variable<br>1.02 (0.90–1.17)	Lower intake than in other studies
27465373 Kim et al., 2016 [35] (**Cohort study**)	Plasma B12 and breast cancer. 124 in total with only 14 breast cancer cases, "canada"	Plasma B12 >332 pmol/L vs. Plasma B12 ≤332 pmol/L	Adjusted incidence HR (95%CI) = 1.12 (0.27, 4.72)	Adjusted for BRCA genotype, alcohol consumption, smoking, parity, coffee consumption
26724465 Arendt et al., 2016 [12] (**Cohort study**)	Serum B12 and breast cancer. 1650 breast cancer identified in health register with serum B12 measurement up to one year prior to diagnosis "Denmark"	S-B12 measured up to 1 year prior to diagnosis<br>Reference group B12 200-600 pmol/L (Reference)<br>601-800<br>> 800	Mortality risk ratio and 95%CI<br>3.6 (1.3–10.0) for 601-800 pmol/L vs. the reference group.<br>3.9 (1.3–11.7) for > 800 pmol/L vs. the reference group.<br>For 31-90d mortality, p value =0.0248; 91-365d mortality p = 0.559	Adjusted for age, sex, calendar year, Charlson comorbidity score index, and cancer stage
31020446 Essen et al., 2019 [36] (**Cohort study**)	Serum B12 and breast cancer. 795 breast cancer out of 19,775 total population from the Swedish AMORIS Cohort	S-B12 tertiles: T2 150-650 pmol/L (Reference group)<br>T1 B12 < 150 pmol/L<br>T3 B12 > 650 pmol/L	HR (95%CI)<br>T1 vs. T2 0.65 (0.29–1.46)<br>T3 vs. T2 0.81 (0.53–1.24)	Adjusted for age, education, socioeconomic status, CCI, serum glucose, triglycerides, cholesterol and fasting status. Follow up time from baseline serum measurement until date of Prostate cancer or

				invasive BC diagnosis, death, emigration or study closing time whichever occurred earlier
**23686442** Bassett 2013 [37] (**Cohort study**)	B12 intake and breast cancer. Follow-up of 20,756 women from the Melbourne Collaborative Cohort Study for an average of 16 years, we ascertained 936 incident breast cancers	Vitamin B12 intake, µg/day<br>Q1 (median intake 1.66) = reference category<br>Q2 (2.33)<br>Q3 (3.04)<br>Q4 (4.61)	(HRs) and 95 % confidence intervals for overall cancer<br>Q2 vs. Q1 1.06 (0.89,1.27)<br>Q3 vs. Q1 0.94 (0.78,1.14)<br>**Q4 vs. Q1 1.21 (1.00,1.46)**<br>Linear model HR **per 1SD increase in B12 intake =** 1.07 (1.00,1.14)	Adjusted for ethnicity, menopausal status, age at menarche, parity and lactation, oral contraceptive use, hormone replacement therapy, physical activity, alcohol consumption, smoking, education and BMI
20410093 Stevens 2010 [38] (**Cohort study**)	B12 intake and breast cancer. 70,656 post-menopausal women for whom dietary information was collected in 1992. Of these, 3898 developed breast cancer within 16 years follow up	Total B12 intake, µg/d<br>Q1 < 1.94 µg/d (reference)<br>Q2 1.94 -<2.80<br>Q3 2.80 - <4.96<br>Q4 4.96 - <9.07<br>Q5 > 9.07	RR (95%CI)<br>Q2 vs. Q1 0.94 (0.85, 1.04)<br>Q3 vs. Q1 0.95 (0.86, 1.06)<br>Q4 vs. Q1 0.96 (0.79, 1.16)<br>Q5 vs. Q1 0.98 (0.80, 1.19)	stratified by age; includes alcohol use, multivitamin use, race, education, first-family history of breast cancer, history of breast lump, hormone replacement therapy, parity and age at first birth, age at menarche, age at menopause, physical activity, BMI, and energy intake.
21447479 Shrubsole 2011 [39](**Cohort**	B12 intake and breast cancer. Prospective cohort	Mean B12 intake in the quintiles, µg/day<br>Q1 1.00 (reference)	HR, hazard ratio (95%CI)<br><br>Q2 vs. Q1 0.89 (0.69, 1.14)	Adjusted for age at baseline, age at menarche, parity, age at

**study**)	Shanghai Women's Health Study (1997–2008) including 718 Chinese breast cancer cases; Non cases (n = 72,519)	Q2 1.83<br>Q3 2.44<br>Q4 3.11<br>Q5 4.60	Q3 vs. Q1 0.88 (0.68,1.15)<br>Q4 vs. Q1 0.91 (0.69, 1.19)<br>Q5 vs. Q1 0.83 (0.61, 1.12)	first livebirth, education, physical activity, use of a B vitamin supplement, height, and total daily intakes of energy, vegetables, and fat and menopausal status.
21736840 Zhang 2011 [40](**Cohort study**)	B12 intake and breast cancer. A hospital-based case–control study, with 438 cases and 438 controls	B12 intake in the controls in µg/d<br>P25 1·01<br>P50 1·54<br>P75 2·27 µg/d	OR (95% CI) =<br>Q2 vs. Q1 0·74 (0·49, 1·09)<br>Q3 vs. Q1 0·74 (0·50, 1·11)<br>Q4 vs. Q1 0·83 (0·56, 1·24)	Matching for age and residence. OR were adjusted for age at menarche, live births and age at first live birth, months of breast-feeding, BMI, history of benign breast disease, first grade relatives with breast cancer, physical activity, passive smoking and total energy intake
23907430 Wu et al, 2013 [41] (Meta-analysis)	S-B12 and B12 intake and breast cancer. 4 studies were identified on plasma concentrations; 14 studies on B12 intake	For S-B12; Wu 1999, Zhang 2003, Lin 2008. For B12 dietary intake: Shrubsole 2001, Lajous 2006, Cho 2007, Lin 2008, Ma 2009, Maruti 2009, Stevens 2010, Chou 2011, Shrubsole 2011, Zhang 2011, Yang 2013, Bassett 2013	RR (95%CI) for B12 concentrations overall 0.73 (0.44–1.22); in subgroups of Pre-menopausal 0.78 (0.34–1.81); in post-menopausal 0.79 (0.47–1.31). Studies on B12 intake:<br>0.88 (0.77–1.00)	

24848140<br>Pathak et al., 2014 [42]	S-B12 among 35 normal cervical controls; 32 squamous intraepithelial lesion; 38 untreated cervical cancer cases, “India”	Serum B12<br>Compared mean (SD) between patients and controls	32 squamous intraepithelial lesion; 38 untreated cervical cancer cases and 35 controls: Mean (SD) = in SILs 377 (121); in untreated cervical cancer 341 (129) pg/ml and 487 (132) in the controls <0.05 for both groups vs. the controls	
22519865<br>Ragasudha et al., 2012 [43]	S-B12 and low grade squamous intraepithelial lesion; and invasive cervical cancer. 92 women with low grade squamous intraepithelial lesion; 94 with invasive cervical cancer; 136 controls “India”	Serum B12<br>serum B12 < 160 pmol/L versus serum B12 >= 160 pmol/L (the high B12 is the reference category)	For the group with low grade squamous intraepithelial lesion the adjusted OR (95%CI) = 10.6 (4.11-27.6).<br><br>For invasive cervical cancer the adjusted OR (95%CI) = 10.4 (3.88-27.9)	Adjusted for age and HPV
17547077<br>Kwanbunjan et al., 2006 [44]	S-B12 and B12 intake and cervical cancers. 44 low grade, 70 high grade, and 95 controls “Thailand”	Serum B12 and B12 intake.<br>Serum B12; T3 > 452 pmol/L (reference cetagory)<br>T2; 277-452 pmol/L<br>T1; < 277 pmol/L<br>B12 intake; T3 > 5.6 µg/d (reference category)<br>T2; 1.22-4.58 µg/L<br>T1; < 277 pmol/L	For exposure S-B12:<br>For low grade cervical cancer and for T2 vs. T3 RR (95%CI) = **3.11 (1.04-9.28)**; For low grade cervical cancer and for T1 vs. T3 RR (95%CI) = **4.08 (1.41-11.79).**<br>For high grade cervical cancer for T2 vs. T3 RR (95%CI) =**2.82 (1.0-8.25)**; for high grade cervical cancer the RR (95%CI) for T1 vs. T3 =**3.53 (1.24-10.04).**<br>For exposure B12 intake:	

			low grade cervical cancer for T2 vs. T3 the R (95%CI) = 0.43 (0.14-1.33); for low grade cervical cancer for T1 vs. T3 the R (95%CI) = **0.11 (0.02-0.48**).<br>For high grade cervical cancer for T2 vs. T3 the R (95%CI) = **0.28 (0.09-0.85**); for high grade cervical cancer for T1 vs. T3 the R (95%CI) = **0.1 (0.02-0.38**).	
19542191 Piyathilake et al., 2009 [45]	S-B12 and cervical cancers. 273 non cases and 103 cases, "USA"	Serum B12	Median S-B12 374,0 vs. 403,0 pg/ml p= 0.361; % of patents with lowered B12 < 200.6 pg /ml n(%) = 19 (18.4%) in cases vs. 28 (10.3)% in the controls; p = 0.032	
21052817 Tong et al., 2011 [46]	S-B12 and cervical cancers. 155 cervical cancers; 165 with cervical intraepithelial neoplasia 1; 167 with cervical intraepithelial neoplasia2; 440 controls<br>"Korea"	Quartiles of serum vitamin B12;<br>Q1 (167–363) is the reference category<br>Q2 (363–514)<br>Q3 (514–680)<br>Q4 (680–1435) pmol/L	OR (95%CI) cervical intraepithelial neoplasia 1<br>Q2 vs. Q1 = 0.97 (0.55–1.74)<br>Q3 vs. Q1 = 0.71 (0.38–1.34)<br>Q4 vs. Q1 = 0.77 (0.41–1.45)<br>cervical intraepithelial neoplasia 2/3<br>Q2 vs. Q1 = 1.25 (0.74–2.13)<br>Q3 vs. Q1 = 0.71 (0.40–1.28)<br>Q 4 vs. Q1 = 0.98 (0.56–1.73).<br>Cervical cancer<br>Q2 vs. Q1 = 0.91 (0.51–1.61)<br>Q3 vs. Q1 = 0.61 (0.33–1.14)<br>Q4 vs. Q1 = 0.76 (0.42–1.38).<br>low B12 <518 pmol/L associated with neoplasia in TT carriers; low	

			B12 <518 pmol/L associated with cervical cancer in TT carriers	

Table S6. Observational studies on plasma/serum B12 or B12 intake and **prostate cancer**. All studies are of case-control design, unless otherwise indicated.				
Reference (PMID author year citation)	Exposure /outcome	High B12 vs. reference category	Effect size	Adjustments/confounders
22927849 Vidal et al., 2012 [47]	272 controls and 144 PC cases, USA	B12 intake high (> 25µg/d) vs. low/normal (≤25 mcg/day)	OR (95%CI) in all PC patients 1.29 (0.78–2.12).<br>In low grade PC: 1.12 (0.56–2.16)<br>In high grade PC: 1.38 (0.74–2.5)	Adjusted for age, race, family history of PC, BMI, and smoking and drinking status
23508410 de Vogel et al., 2013 [48] [overlap with 27061263	P-B12 and P-MMA and prostate cancer. 3000 patients with prostate cancer and	P-B12, pmol/L and P-MMA, µmol/L.<br>Studies OR (95%CI) in B12 Quintiles; <3 40 (Ref.), 340-< 412,412-< 483, 483-< 581, ≥	OR (95%CI) in Q2 vs. Q1 of B12 = 0.95 (0.80–1.13); Q3 vs. Q1 = 0.98 (0.83–1.16); Q4 vs. Q1 = 0.99 (0.83–1.18); Q5 vs. Q1 = 1.10 (0.93–1.31).	Matched for age at serum sampling (6 months), date of serum sampling (2 months) and county of residence

Price et al., 2016 [49]]	3000 controls from JANUS, a population-based bio bank linked to cancer register in Norway	581 pmol/L. MMA Quintiles; < 0.16 (Ref.), 0.16-< 0.18, 0.18-< 0.21, 0.21-< 0.26, ≥ 0.26 µmol/L	OR (95%CI) in Q2 vs. Q1 of MMA = 1.18 (0.99–1.40); Q3 vs. Q1 = 1.15 (0.97–1.36); Q4 vs. Q1 = **1.22 (1.02–1.45**); Q5 vs. Q1 = 1.10 (0.93–1.31). Median (5-95) B12 = 449.8 (252.2–804.0) in patients vs. 446.5 (252.1–780.3) in the controls. Median (5-95) MMA= 0.20 (0.13–0.35) for patients versus 0.20 (0.13–0.36) in the controls.	Adjusted for serum creatinine concentration, education, smoking, physical activity and body mass index
23724740 Collin, 2013 [50][overlap with 27061263 Price et al., 2016 [49]]	S-B12, holoTC, haptocorrin, total transcobalamin and prostate cancer. 3000 (1500 cases with prostate cancer and 1500 controls) From 9 cities in the UK	B12 quartiles; < 239 (Ref.), 239-299, 300-376, > 376pmol/L holoHC quartiles; < 187(Ref.), 187-240, 241-304, >304 pmol/L holoTC quartiles; <42 (Ref.), 42-56, 57-78, >78 pmol/L Total TC quartiles; <749 (Ref.), 749-874, 875-1024, >1024 pmol/L	OR (95%CI) for B12 quartiles Q2 vs. Q1 = 0.91 (0.74-1.12); Q3 vs. Q1 = 0.99 (0.80-1.22); Q4 vs. Q1 = 1.17 (0.95-1.43). holoHC quartiles: Q2 vs. Q1 = 0.94 (0.76-1.16); Q3 vs. Q1 = 0.99 (0.80-1.22); Q4 vs. Q1 = **1.27 (1.04-1.56).** holoTC quartiles: Q2 vs. Q1 = 1.08 (0.88-1.33); Q3 vs. Q1 = 1.09 (0.89-1.34); Q4 vs. Q1 = 1.04 (0.84-1.28). total tc quartiles: Q2 vs. Q1 = 1.05 (0.86-1.29); Q3 vs. Q1 = 1.02 (0.83-1.25); Q4 vs. Q1 =0.84 (0.68-1.05).	matching on 5-y age group and recruiting center, further adjusted for exact age (continuous), using quartile means as a linear variable
15499634 Hultdin et al., 2005 [51]	S-B12 and prostate cancer. 254 cases with prostate	S-B12 in pmol/L B12 quartiles; Q1 <238, Q2 238-302, Q3 302-370, Q4	Mean (SD) of B12 = 329 (100) in patients versus 300 (104) in the controls, p<0.001.	Matching for age, recruitment date, sub cohort

[overlap with 27061263 Price et al., 2016]	cancer and 514 controls<br><br>"Sweden"	>370 pmol/L	Unadjusted OR (95%CI) for Q2 vs. Q1 = **1.88 (1.17-3.01); for Q3 vs. Q1 = 2.12 (1.31-3.42); for Q4 vs. Q1 = 2.63 (1.61-4.29)**	
18268110 Johansson et al., 2008 [52]	S-B12 and prostate cancer. 869 cases with prostate cancer and 1174 controls. Combined 6 cohorts: ATBC, CARET, EPIC 1 and 2, Janus, NSHDC, ProtecT. Analysis of individual participant data from 6 cohorts "Europe"	S-B12 quintiles; Q1 <247.4 (reference); Q2 247.4-299.7; Q3 299.9-352.7; Q4 353.0-424.8; Q5 425.0 pmol/L	RR (95%CI) for Q2 vs. Q1 = 1.15 (0.85-1.55); for Q3 vs. Q1 =1.15 (0.85-1.55); for Q4 vs. Q1 = 1.23 (0.90-1.69); for Q5 vs. Q1 = 1.19 (0.87-1.63)	Matched for age, fasting status, center, blood collection time and further adjusted for BMI, smoking, alcohol, physical activity, marital status, education
27061263 Price et al., 2016 [49]	S-B12 and prostate cancer. 6735 cases and 7959 controls "Europe"	S-B12 and its study specific quintiles.	pooled estimate OR (95%CI) for the upper 20% versus the lowest 20% in the study specific B12 concentrations across 6 studies = 1.12 (1.02-1.24)	age at blood collection, BMI, education, height, marital status, smoking
26724465 Arendt et al., 2016 [12] (**Cohort study**)	S-B12 and mortality after prostate cancer diagnosis. 2499 prostate cancer identified in health register with S-B12	S-B12 measured up to 1 year prior to diagnosis Reference group B12 200-600 pmol/L (Reference)<br>601-800<br>> 800	mortality risk ratio and 95%CI<br>S-B12 601-800 pmol/L vs. the reference category =1.9 (0.7–5.2)<br>S-B12 > 800 pmol/L vs. the reference category = 2.8 (1.3–5.9).<br>In addition, the 31-90d	Adjusted for age, sex, calendar year, Charlson comorbidity score index, and cancer stage

	measurement up to one year prior to diagnosis		mortality, p value =0.83; 91-365d mortality p = 0.80	
31020446 Essen et al., 2019 [36] (**Cohort study**)	S-B12 and prostate cancer. 703 prostate cancer cases out of 8783 total population from the Swedish AMORIS Cohort	S-B12 tertiles<br>150-650 pmol/L (Reference group<br>B12 < 150 pmol/L<br>B12 > 650 pmol/L	HR (95%CI) for B12 < 150 pmol/L versus 150-650 pmol/L = 0.92 (0.43–1.95); HR (95%CI) for B12 > 650 pmol/L versus 150-650 pmol/L = 0.99 (0.61–1.62)	Adjusted for age, education, SES, CCI, serum glucose, triglycerides, cholesterol and fasting status. Follow up time is time from baseline serum measurement until date of Prostate cancer or invasive BC diagnosis, death, emigration or study closing time, whichever occurred earlier
20852008 Collin et al, 2010 [53] (**Cohort study**)	Serum B12, holoTC, holoHC, total transcobalamin. Outcome PSA velocity > 2 ng/ml/y (a proxy measure of progression of localized prostate cancer). 424 men followed for 2.5 years. Outcome PSA velocity > 2 ng/ml/y (a proxy measure of	Serum B12, holoTC, holoHC, total transcobalamin<br>The outcome was analyzed as a continuous variable and as a categorical variable.	OR (95%CI) for PSA velocity > 2 vs. ≤ 2 ng/ml/y per unit increase in log metabolite concentrations: B12: OR (95%CI)=0.64 (0.30-1.35). HoloHC: OR (95%CI)=0.63 (0.32-1.25). holoTC: OR (95%CI)=1.00 (0.57-1.77). total TC: OR (95%CI)=0.79 (0.23-2.77). Change in mean PSA velocity per unit increase in loge vitamin marker:<br>coefficient (95%CI) for B12 =-0.08 (-0.59-0.43). For holoHC: 0.08 (-0.55-0.40). HoloTC =-0.04 (-0.43-0.34). total TC =-0.30 (-1.14-0.53).	Adjusted for age and Gleason score

	progression of prostate cancer), "UK"			
27061263 Price et al., 2016 [49] (**Analysis of individual participant data**)	S-B12 and prostate cancer in ATBC, CARET, EPIC 1 and 2, Janus, NSHDC, ProtecT cohorts. 6735 cases and 7959 controls. Combination of several cohorts: ATBC, CARET, EPIC 1 and 2, Janus, NSHDC, ProtecT. Analysis of individual participant data from 6 cohorts.	pooled estimate OR (95%CI) for the upper 20% versus the lowest 20% in the study specific S-B12 concentrations across 6 studies	random effect model OR (95%CI) = 1.12 (1.02-1.24)	
23724740 Collin et al., 2013 [50](**Meta-analysis**)	Includes the following studies: Weinstein 2003; Hultdin 2005; Johansson 2008; Figueieredo 2009; Ebbing 2009; Protect 2009	Analyzed plasma B12	random effect model per 100 pmol/L higher B12 pooled OR = 1.10 (1.01-1.19)	

Table S7. Observational studies on plasma/serum B12 or B12 intake and **Kidney and urothelial bladder cancers**				
Reference (PMID author year citation)	Exposure /outcome	High B12 vs. reference category	Effect size	Adjustments/confounders
35129646 Boot et al., 2022 [54]	Pooled analysis of prospective cohort studies including data from 2915 bladder cancer and 530,012 controls	Intake of vitamin B12 among other B vitamins. The study used tertiles on the intake (low/moderate/high intake) based on the distribution of all included study participants per study separately	Hazard ratios (HRs) and corresponding 95% CI. Inverse associations were found HRB12: 0.79, 95% CI: 0.66–0.95. Slightly decreased risk for higher intake of vitamin B12 (p = 0.002)	Adjustments for age, sex, smoking, water (low/high).
31058547 Ben Fradj et al.,	S-B12 and Urothelial	S-B12, ng/L divided into tertiles	OR (95%CI) = estimated from the figure 2; for T2 vs. T1 OR =	Adjusted for age, sex, BMI, tobacco exposure

2019 [55]	bladder cancer		1,45 (0,9-2,3); for T3 vs. T1 OR = 1,5 (0,8-2,6)	
30694528 Vrieling et al., 2019 [56]	Vitamin B12 intake in µg/d and S-B12, pmol/L And urothelial bladder cancer. 824 pairs of cancer patients (urothelial bladder cancer) and controls "EPIC Cohort"	Vitamin B12 intake in µg/d and S-B12, pmol/L Quartiles of B12 intake in the controls; Q1 ≤4.76; Q2 4.76-6.58; Q3 6.63-8.93; Q4 ≥8.91 (reference category) µg/d. Quartiles of S-B12 concentrations in the controls; Q1 ≤ 214; Q2 215-270; Q3 271-346; Q4 ≥ 347 (reference category) pmol/L.	For B12 intake levels OR (95%CI) for urothelial bladder cancer in Q1 vs. Q4 = 0.79 (0.53-1.16); Q2 vs. Q4 = 0.94 (0.67-1.33); Q3 vs. Q4 = 0.92 (0.67-1.27). For S-B12 concentrations, OR (95%CI) for urothelial bladder cancer in Q1 vs. Q4 = 0.94 (0.96-1.28); Q2 vs. Q4 = 0.84 (0.61-1.15); Q3 vs. Q4 = 1.02 (0.76-1.37)	Matched for age at enrolment, sex, time of day blood collection, date of blood collection, fasting status
25376861 Johansson, et al., 2014 [57]	S-B12, pmol/L and renal cancer; Outcomes are renal cancer and all cause mortality. 556 cases with renal cancer and 556 controls both from the EPIC study; and a second control group of 553 controls (control group 2) matched to the	S-B12, pmol/L Quartiles of B12 concentrations in the controls; Q1 ≤281 (reference category); Q2 281-343; Q3 344-419; Q4 419-5000 pmol/L	OR (95%CI) for renal cancer in Q2 S-B12 vs. Q1 0.96 (0.67 to 1.37); Q3 vs. Q1 = 0.87 (0.61 to 1.26); Q4 vs. Q1 = 0.67 (0.46 to 0.99). All cause mortality HR (95%CI) for Q2 vs. Q1 = 0.96 (0.66 to 1.41); for Q3 vs. Q1 = 0.75 (0.49 to 1.14); for Q4 vs. Q1 = 0.93 (0.61 to 1.41)	Matched by sex, date of blood collection, and date of birth. Outcome renal cancer; analyses adjusted for waist to hip ratio, hypertension, education, smoking, plasma cotinine, alcohol intake. Outcome all cause mortality, the analyses were adjusted for country, sex, age at diagnosis, quartiles of vitamin B6, hypertension, waist-to-hip ratio, education, smoking, cotinine, alcohol,

	cases from a parallel study. "EPIC cohort"			BMI
20383577 Gibson et al., 2010 [58]	B12 intake and renal cell carcinoma. 224 patients and 224 matched controls "Finland"	Both B12 intake and S-B12 were measured. S-B12 divided into quartiles: Q1 ≤286.41 (Reference category); Q2 286.42 – 353.07; Q3 353.08 – 433.38; Q4 >433.38 pmol/L	B12 intake was median and interquartile ranges = 10.4 (7.3-13.0) in patients and 10.2 (7.9-12.7) in the controls, p = 0.53 The OR (95%CI) for incident renal cancer in Q2 vs. Q1 of vitamin B12 = 0.63 (0.34-1.16); Q3 vs. Q1 = 0.93 (0.49-1.76); Q4 vs. Q1 = 0.87 (0.46-1.63)	Matching for age at randomization, date of blood collection
29968964 Bock et al., 2018 [59]	B12 intake and Renal cell carcinoma. The US Kidney cancer case control study; 1142 cases and 1154 controls	B12 intake > 6.6 µg/d (reference category); 3.7-5.0 g/d; <=3.6 µg/day	B12 intake µg/d mean (SD) = in white cases 5.6 (0.08) vs. 5.5 (0.08) in while controls; in African American cases 5.5 (0.14) vs. 5.2 (0.13) in African American controls OR (95%CI) for overall renal cancer: B12 intake 5.1-6.6 µg/day vs. > 6.6 µg/d 1.1 (0.8-1.5); 3.7-5.0 g/d vs. > 6.6 µg/d 1.1 (0.8-1.5); <=3.6 µg/day vs.> 6.6 µg/d 1.0 (0.7-1.5).	Adjusted for total calories, region, sex, race, age, education, smoking history, BMI, family history of RCC, hypertension, and alcohol intake
33074725 Ben Fradj et al., 2021 [60](Cohort study)	S-B12 and bladder cancer. 177 patients with bladder cancer "Tunis"	Measured S-B12 and stratified by tertiles and studied the prognostic value of S-B12 in Non-Muscle-Invasive Bladder Cancer (lowest tertile is the reference group. Follow up was up to 5 years (median follow up	HR (95%CI) for progressive cancer in S-B12 tertile 2 versus the lowest B12 tertile = 1.28 (0.35-4.72); for tertile 3 versus the lowest B12 tertile =2.12 (0.63-7.21)	Adjusted for age, BMI, sex, smoking, alcohol, occupation, immunetherapy, tumor stage, grade, number and size

		66 months up to 5 years)		
26724465 Arendt et al., 2016 [12] (Cohort study)	Serum B12 and kidney cancer and urinary bladder cancers. 557 kidney cancer and 541 urinary bladder cancers identified in health register with serum B12 measurement up to one year prior to diagnosis	S-B12 measured up to 1 year prior to diagnosis<br>B12 200-600 pmol/L (Reference)<br>601-800<br>> 800<br>B12 deficiency excluded. Data source (health register data, DK)	For kidney cancer pB12 601-800 pmol/L vs. the reference category mortality risk ratio and 95%CI =2.9 (1.1–7.5); for S-B12 > 800 pmol/L, the mortality risk ratio and 95%CI = 0.9 (0.3–3.1).<br>31-90d mortality, p value =0.27; 91-365d mortality p = 0.535.<br>For urinary bladder cancers S-B12 601-800 pmol/L vs. the reference category mortality risk ratio and 95%CI = 0.6 (0.1–2.4); for S-B12 > 800 pmol/L, the mortality risk ratio and 95%CI = 3.0 (1.3–6.8).<br>31-90d mortality, p value = 0.281; 91-365d mortality p = 0.716.	Adjusted for age, BMI, sex, smoking, alcohol, occupation, immunetherapy, tumor stage, grade, number and size
23242637 Cho et al., 2013 [61] (Cohort study)	B12 intake and renal cell cancer. Women in the Nurses' Health Study (NHS) and men in the Health Professionals	B12 intake adjusted for caloric intake and divided into quintiles (lowest quintile is the reference category).	For total B12 intake, multivariate adjusted RR (95%CI) in NHS for Q2 vs. Q1 = 1.06 (0.67 – 1.68); Q3 vs. Q1 1.03 (0.65 – 1.62); Q4 vs. Q1 1.10 (0.70 – 1.72); Q5 vs. Q1 1.25 (0.81 – 1.93).<br>In HPFS: Q2 vs. Q1 1.65 (1.05	adjusted for age (months), smoking status (never, past, current), body mass index (continuous), history of hypertension (yes/no), history of diabetes (yes/no), physical activity (quintiles), fruit intake (continuous),

	(HPFS) Follow-up Study During follow-up of 24 years among 77,208 women (918,891 person-years) and 22 years among 47,886 men (1,731,752 person-years), we accrued 436 cases of RCC (225 women and 211 men).		– 2.59) ; Q3 vs. Q1 1.33 (0.84 – 2.12); Q4 vs. Q1 1.18 (0.74 – 1.90); Q5 vs. Q1 1.22 (0.76 – 1.96).<br>In both studies combined: Q2 vs. Q1 1.32 (0.86 – 2.05); Q3 vs. Q1 1.17 (0.84 – 1.61) ; Q4 vs. Q1 1.14 (0.82 – 1.58); Q5 vs. Q1 1.24 (0.90 – 1.70)<br>B12 intake from foods only gave similar insignificant results.	vegetable intake (continuous), and alcohol intake (continuous) in NHS and HPFS and parity (nulliparous, 1–2, 3, 4, 5+ children) in NHS.
32162043 Clasen et al., 2020 [62](Meta-analysis)	2 independent studies on intake (Cho 2013 and Bock 2018)<br>2 studies on B12 biomarkers (Johansson 2014 and Gibson 2010)	Intake levels and serum concentrations were analyzed separately.	The pooled effect size from studies on intake 1.14 (0.87, 1.49)<br><br>The estimate from 2 studies with biomarkers; 0.73 (0.51, 1.06)	Limitations due to low number of studies.

Table S8. Observational studies on plasma/serum B12 or B12 intake and **colorectal cancers**.				
Reference (PMID author year citation)	Exposure /outcome	High B12 vs. reference category	Effect size	Adjustments/confounders
1809232 Dahlin et al., 2008 [63]	P-B12 and colorectal cancer. Nested case control study with 226 patients with colorectal cancer and 436 controls based on 3 Swedish cohort studies	Plasma B12, pmol/L Quintiles of B12 in the controls: men 133.9–220.2, 220.2–258.7, 258.7–297.2, 297.2–351.2, 351.2–673.3 pmol/L and women: 130.0–232.1, 232.1–275.9, 275.9–	Adjusted OR (95%CI) for Q2 vs. Q1 =0.78 (0.46–1.31); Q3 vs. Q1 = **0.54 (0.30–0.95);** Q4 vs. Q1 = 0.82 (0.47–1.42); Q5 vs. Q1 = 0.82 (0.46–1.45). Median B12 in the patients = 282 (225-353) versus 290 (238-357) pmol/L in the controls, p = 0.225	Matched for age, sex, cohort, date of health survey and fasting status and additionally adjusted for BMI, smoking, physical activity, alcohol intake, and plasma folate and total homocysteine

		322.6, 322.6–391.9, 391.9–836.1. Q1 is the reference group.		
34645434 Ghorbani et al. 2021 [64]	S-B12 and colon cancer. 19 patients and 23 controls from Iran	S-B12, pg/ml	Compared median (absolute deviation) =298 (130) in the patients and 293 (128) in the controls, p =0.951	Confounding by imbalanced age; controls are sig. younger. The study has limitation; small sample size, confounding, etc
32686693 Liu et al., 2020 [65]	patients with colon cancer and non-tumor adjuvant tissues; Studied mRNA expression of TCNI in colon cancer tissues and adjacent tissues. . Studied 194 patients with colon cancer and n=349 non-tumor adjuvant tissues "China"	mRNA expression of TCNI	1- The relative mRNA expression level of TCN1 in cancer tissues was higher than in non-cancerous tissues (0.601 ± 0.024 vs. 0.335 ± 0.018, P < 0.001). protein expression of TCNI was also increased.<br>2- TCN1 gene expression in tumor tissues was decreased after chemotherapy (P = 0.009); the identified TCN1-related gene products were mainly expressed in the cell membrane, Golgi apparatus, endoplasmic reticulum, and vesicles, and are involved in cytokine production, protein glycosylation, apoptosis, exocytosis, and vesicle transport.	The study provides mechanistic explanation of high B12 in cancer.
26108676 Cheng et al., 2015 [66]	P-B12 and colon cancer 821 cases and 821 controls (nested case-control study)<br>"USA"	Plasma B12, pg/ml	mean (SD) B12 in patients = 515 (280) versus controls = 529 (264) pg/ml, p = 0.06	Matching by age, race/ethnicity, enrollment date, hysterectomy status, family history, time of blood draw

20813848 Eussen et al., 2010 [67]	P-B12 and P-MMA and colorectal cancers. 1365 incident CRC cases (colon n=846; rectal n=519) and 2319 controls nested within the EPIC cohort "Europe"	Measured P-B12, pmol/L and MMA, µmol/L.<br>B12; Q1 < 220 (Reference)<br>Q2 220-266<br>Q3 266-312<br>Q4 312-380<br>Q5 > 380<br>MMA Q1 < 0.14 (Reference)<br>Q2 0.14-0.16<br>Q3 0.16-0.18<br>Q4 0.18-0.22<br>Q5 > 0.22	B12: median (5-95th) percentiles in patients = 288 (162 - 498) vs. 288 (161 - 501) pmol/L in the controls, p = 0.97<br>Median (5-95th) percentiles of MMA in patients = 0.17 (0.12 - 0.32) vs. 0.17 (0.12 - 0.30) in the controls, p =0.32.<br>RR (95%CI) for B12 Q2 vs. Q1 0.86 (0.69; 1.08)<br>Q3 vs. Q1 1.02 (0.81; 1.28)<br>Q4 vs. Q1 1.08 (0.85; 1.35)<br>Q5 vs. Q1 1.02 (0.80; 1.29).<br>For MMA Q2 vs. Q1 1.09 (0.87; 1.36)<br>Q3 vs. Q1 1.16 (0.92; 1.46)<br>Q4 vs. Q1 1.18 (0.94; 1.49)<br>Q5 vs. Q1 0.94 (0.74; 1.21).	matched and co-variates adjusted for smoking status, education, physical activity, fiber intake, intake of red and processed meat, alcohol consumption, and BMI.
19736606 Bystrom et al., 2009 [68]	S-B12 and overall survival in patients with advanced colorectal cancer. 81 Cases with advanced colorectal cancer. Patients follow up during 5-FU therapy "Sweden"	S B12, pmol/L	patients with B12 values < 300 pmol/L at base-line had better overall survival (P 0.0002) and Time to progression (P 0.03) than patients with higher B12 value. Chemotherapy did not significantly affect serum B12	
19336555 Lee et al., 2009 [69]	P-B12 and colon cancer. 197 men cases with colorectal cancer and 371 controls nested within the Physician Health Study "USA"	Plasma B12, pg/ml	median (25,75th) percentiles men cases 434 (374-575) versus 467 (364-572)pg/ml in the controls, p =0.93	Matched for age and smoking (in all patients except 23), all were men

17921385 Al-Ghnaniem et al., 2007 [70]	S-B12 and cancer, adenoma, or hyperplastic polyps. 28 Cancer; 35 Adenoma 17 Hyperplastic polyps and 76 controls "UK"	S-B12, pmol/L	Median (range) in the controls = 311 (87-926) vs. in cancer =225 (95-515) pmol/L (p = 0.006 vs controls); in adenoma = 278 (64-791) (non sig. vs. the controls); 314 (52-941) in hyperplastic polype (non sig. vs. controls)	
23441607 Chen et al., 2013 [71]	S-B12 and colorectal polyps (adenomatous polyps, hyperplastic polyps). 48 cases with colorectal polyps (29 adenomatous polyps 19 hyperplastic polyps) and 96 controls "Taiwan"	S-B12, pmol/L	Mean (SD) of S-B12 in adenomatous polyps (considered precursors of cancer) = 334 (189) versus 373 (205) in the controls; mean (SD) = 355 (162) in hyperplastic polyps versus 373 (205) pmol/L in the controls (both p values are not significant.	age and sex matched controls
26724465 Arendt et al., 2016 [12] (cohort study)	Serum B12 and survival after diagnosis of colorectal cancers. 3441 colorectal cancer identified in health register with S-B12 measured up to one year prior to diagnosis (health register data, Denmark)	S-B12 measured up to 1 year prior to diagnosis. B12 = 200-600 pmol/L (Reference); 601-800; and > 800 pmol/L. B12 < 200 pmol/L excluded	plasma B12 601-800 pmol/L vs. the reference category mortality risk ratio and 95%CI =1.6 (1.0–2.5); for plasma B12 > 800 pmol/L, the mortality risk ratio and 95%CI =2.8 (1.9–4.1).<br>In addition, 31-90d mortality, p value =0.47; 91-365d mortality p = 0.066	Adjusted for age, sex, calendar year, Charlson comorbidity score index, and cancer stage
32753569 Zhu et al. 2020 [72] (cohort study)	Tissue TCNI and survival in patients with colorectal cancers. 123 patients with colorectal cancers "China"	Immunohistochemistry, semiquantitative assay of TCNI in tissues studied 5y survival	For the outcome 5-yrs survivals after colorectal cancer, staining with TCN1 in tissues has shown that the survival of patients in the low TCN1 expression group was longer than that in the medium and high TCN1	

			expression groups (P=0.045). 5-year survival in patients with low, medium, and high levels of TCN1 immunoexpression were 88.9%, 50.0%, and 40.0%, respectively. But, higher TCN1 immunoexpression was associated with larger and invasive tumors, higher tumor markers, metastasis to regional lymph.	

Table S9. Observational studies on plasma/serum B12 or B12 intake and lung cancers.				
Reference (PMID author year citation)	High B12 vs. reference category	Effect size	Calculations most contrasting groups	Adjustments/confounders
31738590 Lo-Bisgaard et al., 2020 [73]	728 (analyzed) with lung cancer and 161 controls "DK"	S-B12, pmol/L; serum Haptocorrin, pmol/L; serum transcobalamin, pmol/L; serum holoTC, pmol/L quartiles B12; Q1 (ref) 89–289, Q2 290–374, Q3 375–484, Q4 485–2655 pmol/L; quartiles haptocorrin; Q1	Mean (range) = 399 (133–1878) in patients with lung cancer, and 377 (89–2655) in the controls, p = ns. OR (95%CI) for S-B12 Q2 vs. Q1 = 0.95 (0.58–1.57); Q3 vs. Q1 = 0.96 (0.58–1.58); Q4 vs. Q1 =	Adjusted for gender, age and smoking.

		(ref) 230–500, Q2 501–611, Q3 612–739, Q4 740–3940 pmol/L; quartiles transcobalamin; Q1 (ref) 410–910 Q2 911–1050 Q3 1051–1240 Q4 1241–664; quartiles holoTC; Q1 (ref) 38–85 Q2 86–122 Q3 113–149 Q4 150–4560 pmol/L	1.12 (0.69–1.82).<br>OR (95%CI) for haptocorrin Q2 vs. Q1 = 0.90 (0.53–1.51); Q3 vs. Q1 = 1.05 (0.62–1.76); Q4 vs. Q1 = **1.84 (1.13–3.00).**<br>OR (95%CI) for transcobalamin Q2 vs. Q1 =1.28 (0.76–2.17); Q3 vs. Q1 =1.25 (0.74–2.11); **Q4 vs. Q1 = 1.78 (1.08–2.93).**<br>OR (95%CI) for holoTC Q2 vs. Q1 =0.77 (0.47–1.25); Q3 vs. Q1 = 0.57 (0.34–0.96) ; Q4 vs. Q1 = 0.86 (0.54–1.38).	
30499135 Fanidi et al., 2019 [74]	5183 cases and same n as controls. Nested within 20 individual prospective cohort studies participating in the Lung Cancer Cohort Consortium (LC3) "EU, Australia, Asia"	S-B12, pmol/L.<br>quartile of B12; Q1 (61.1-338.3) (Q1 = Ref), Q2 (338.4-425.6), Q3 (425.7-531.0), Q4 > 531.1	Overall lung cancer OR (95%CI) for Q2 vs. Q1 = 1.10 (0.98 - 1.23); Q3 vs. Q1 = **1.16 (1.03 - 1.3); Q4 vs. Q1 = 1.19 (1.05 - 1.34).**<br>OR-logB12 (95%CI) as a continuous variable adjusted for cotinine and education:<br>Overall lung cancer<br>1.15 (1.06-1.25)<br>Large cell carcinoma; **1.95 (1.16-3.27)**; small cell carcinoma = 1.20 (0.91-1.59); squamous cell carcinoma = 1.00 (0.81-1.23); Adenocarcinoma = **1.14 (1.00-1.30)**.	matched cases, adjusting for circulating cotinine (in quartiles) and education (in 7 categories)
30499135 Fanidi et al., 2019 [74]	Mendelian randomization study on Genetic data of 29266 cases	8 Genetic variants related to B12	The OR (95%CI) for<br>Overall lung cancer = **1.08 [1.00-1.16]);**<br>small cell carcinoma = 1.17 [0.96-	Not clear which genetic variants and in which direction is the association between the

	and 56450 controls		1.41];<br>squamous cell carcinoma =0.97 [0.86-1.10];<br>Adenocarcinoma =**1.23 [1.11-1.37].**	variants and the B12 concentrations
26422108 Tastekin et al., 2015 [75]	40 cases with lung cancer and 40 controls. "Turkey"	s- B12, ng/ml	mean (SD) of S-B12 in patients = 234 (99) versus 240 (104) ng/ml in the controls, p = 0.78	
26724465 Arendt et al., 2016 [12](cohort study)	3161 lung cancers identified in health register with serum B12 measurement up to one year prior to diagnosis	S-B12 measured up to 1 year prior to diagnosis of cancer and 30 days mortality risk ratio. Reference group B12 200-600 pmol/L. B12 deficiency excluded. Data source health register data, Denmark.	plasma B12 601-800 pmol/L vs. the reference category. Mortality risk ratio and 95%CI =1.4 (1.1–1.8); for plasma B12 > 800 pmol/L, the mortality risk ratio and 95%CI =1.9 (1.4–2.4).<br>In addition, 31-90d mortality, p value = 0.005; 91-365d mortality p = 0.33	Adjusted for age, sex, calendar year, Charlson comorbidity score index, and cancer stage
**32030745 Brasky et al., 2020 [76]** (cohort study)	B12 intake and primary lung cancers. 3836 (Histology data from 2,466 (64%) cases) primary lung cancers in the Women Health Initiative (prospective study including observational cohort and also 4 clinical trails) total cohort in the WHI	Studied B12 intake; no B12 intake from supplements; intake >= 25 µg/d; 25-106 µg/d; >= 106 µg/d; and measured serum B12 concentrations, pmol/L	Suppl. vitamin B12 was not associated with lung cancer risk among women. Use of B12 from individual suppl. sources, but not from multivitamins, was associated with a 30% to 40% increase in lung cancer risk among men. When the 10-y average supplement dose was evaluated, there was an almost two-fold increase in lung cancer risk among men in the highest categories of vitamin B6 (> 20 mg/d; hazard ratio, 1.82; 95% CI, 1.25 to 2.65) and B12 (> 55µg/d; hazard ratio, 1.98; 95% CI, 1.32 to	Adjusted for age, ethnicity, smoking status, years smoked, pack-years and pack-years squared. Analyses including dietary B vitamins additionally adjusted for total energy.<br>cases and those who remained free of the outcome were different in many risk factors such as age, alcohol, health eating index, hormone therapy, ethnic origin, etc

	(n = 159,232)		2.97)	
**33656837 Luu et al., 2021 [77]**	Singapore Chinese Health Study, a prospective cohort study of 63 257 Singaporean Chinese men and women, 45-74 years of age at enrollment during 1993-1998 and were followed up for incidence of lung cancer for up to 25 years.	Vitamin B12 intake (μg/d), Mean±SD<br>2.33±0.89 (Reference)<br>1.19±0.43<br>1.89±0.12<br>2.28±0.11<br>2.70±0.14<br>3.61±0.71	hazard ratio and 95% confidence interval (CI) – overall lung cancer<br>1.09 (0.95–1.25)<br>1.11 (0.96–1.28)<br>1.11 (0.97–1.29)<br>**1.18 (1.03–1.35)**<br>Adenocarcinoma<br>1.20 (0.96–1.51)<br>**1.34 (1.07–1.67)**<br>**1.25 (1.00–1.57)**<br>By follow up ≤ 2years<br>**1.77 (1.00–3.13)**<br>**1.84 (1.04–3.26)**<br>**2.34 (1.36–4.04)**	The association was more apparent in men than in women, in patients with adenocarcinoma, or in those with ≤ 2yrs follow-up than those with longer duration of follow-up. Adjusted for age, sex, education, dialect group, year of enrollment, smoking habits, dietary beta-cryptoxanthin, caffeine intake, caloric intake.

Table S10. All other cancers and studies with **mixed types of cancer** (case control studies or nested case control studies)

Reference (PMID author year citation)	High B12 vs. reference category	Effect size	Calculations most contrasting groups	Adjustments/confounding
35684053 Bo et al., 2022	total of 55,569 adults from the 3rd	Hazard Ratios (HRs) and 95% confidence intervals	In men<br>0.97 (0.84–1.11)	age, race/ethnicity, BMI, family income–poverty ratio,

[78]	National Health and Nutrition Examination Survey (NHANES III) and NHANES 1999–2014	(CIs) for all-cause and cause-specific mortality according to the quintiles of dietary vitamin	1.08 (0.93–1.27)<br>0.96 (0.79–1.16)<br>In women<br>1.03 (0.88–1.20)<br>1.05 (0.88–1.26)<br>1.05 (0.84–1.31)	smoking status, drinking status, leisure-time physical activity, total energy intake, diabetes, hypertension, and cardiovascular disease
25879336 Kharb et al., 2015 [79]	S-B12 and Osteosarcoma. 30 cases and 30 controls "India"	Serum B12, pg/ml	mean (SD) in patients = 236 (122) vs. 523 (207) pg/ml in the controls; p = <0.001	Patients were age and sex matched with the controls
21227009 Gorgulu et al., 2010 [80]	S-B12 and laryngeal squamous cell carcinoma. 60 patients with Laryngeal squamous cell carcinoma and 30 controls never smokers; and 30 controls current smokers, Turkey	Serum B12, pg/ml	mean (SD) B12 in patients = 207 (124) versus 254 (115) pg/ml in current smokers p <0.05; and mean (SD) = 207 (124) in patients versus 292 (104) pg/ml in never smokers p <0.05	No adjustments
15593092 Almadori et al., 2005 [81]	S-B12 and head and neck squamous cell carcinoma, and laryngeal leukoplakia. 144 patients with head and neck squamous cell carcinoma, 40 patients with laryngeal	Serum B12, pg/ml mean (SD) were compared between patients and controls (the controls were stratified according to smoking status)	mean (SD) = 429 (281) for head and neck squamous cell carcinoma 480 (256) pg/ml in 120/non smokers controls;<br>mean (SD) = 429 (281) in for head and neck squamous cell carcinoma versus 472 (225) pg/ml in 90/smokers;<br>Mean (SD) = 373 (152) in laryngeal leukoplakia versus 480	age, sex, and smoking matched

	leukoplakia and 210 controls, Italy		(256) pg/ml in 120/non smokers controls; or vs. 472 (225) pg/ml in in 90/smokers.<br>All p for case-control comparisons not significant	
19075131 Nacci et al., 2008 [82]	S-B12 and laryngeal Squamous cell carcinoma. 25 cases with Laryngeal Squamous cell carcinoma and 80 controls "Italy"	Serum B12, pg/ml Mean (SD) were compared	Mean (SD) in patients = 385 (278) versus 498 (125) pg/ml in the controls, p <0.01	Regression analyses adjusted for smoking status and plasma homocysteine and folate concentrations
31202497 Wu et al., 2019 [83]	S-B12 among 131 patients with oral precancers **(early stage of cancer)** and 131 matched controls, Taiwan	Serum B12, pg/ml	mean (SD) in the patients = 531 (254) versus 623 (181) pg /ml in the controls, p =0,001.<br>43,5% of the patients had S-B12 ≤450 pg/ml (vs. 13% of the controls); p <0.001	Age and sex matched controls
29220583 Oh et al., 2018 [84]	Cross sectional study on patients with several kinds of cancer "Korea"	The association between S-B12 and survival time in patients with various solid tumors. Studied 30 and 90 days survivals in patients with normal B12 211-911 vs. those with high B12 > 911 pg/ml	percentage 30 days mortality 38.9% (32.6–44.8) at high B12 vs. 10.6% (7.3–14.6) at normal B12; percentage 90 days mortality 63.8% (57.5–70.6) at high B12 vs. 34.1% (29.5–40.1) at normal B12	Patients with low B12 were excluded (will overestimate the association, especially that a U shape curve is possible), patients treated with B12 were excluded
23550564 Ryg et al., 2013 [85](cohort study)	Longitudinal within the health register data (n = 40391), compared to age-specific cancer	P-B12 > 1200 pmol/L (total n = 490) and cancer diagnosis within 6-36 months from B12 measurement; outcome	Cancer incidence among subjects with elevated B12 (6.7%, 95% CI: 4.7–9.3%) in the first 6 months after B12 test versus (2.6%, 95% CI: 2.4–2.7%) for people with	Indirectly standardized by age (5 yrs) and sex (observed to expected cancer cases). SMR (morbidity ratio) in the first 6 months after B12 assay= 15.1

	incidence in the general Danish population (retrieved from NORDCAN) "DK"	detection of any cancer after measurement of B12. Plasma B12 > 1200 pmol/L (total n = 490) and cancer diagnosis within 6 months from B12 measurement; or cancer diagnosis within 6-12 months from B12 measurement; or within 12-24 months from B12 measurement; or within 24-36 mon: or within > 36 months from B12 measurement.	normal B12. 33 of 490 with high B12 (vs. 1024 of 40104 with normal B12) developed cancer from 0-6 months; 6 persons with high B12 (vs. 309 with normal B12) developed cancer at 6-12 months after B12 assay; 5 persons with high B12 (vs. 410 with normal B12) developed cancer at 12-24 months after B12 assay; 5 persons with high B12 (vs. 268 with normal B12) developed cancer at 24-36 after B12 assay; 5 persons with high B12 (vs. 523 with normal B12) developed cancer at > 36 months post B12 assay.	(CI 10.4–21.2) for subjects with high B12 and 5.0 (CI 4.7–5.3) for those with B12 < 1200 pmol/L
26365156 Maritsi et al., 2016 [86]	P-B12 and solid tumors in children. 42 children with solid tumors and 42 controls; mean age 4.5 yrs "Greece"	Plasma B12, pg/ml and compare the mean (SD) between patients and controls	mean (SD) = 260 (116) in patients versus 574 (139) pg/ml in the controls, p = 0.009	Age and sex matched controls
26237587 Ilhan et al, 2015 [87]	P-B12 and pediatric cancers not further specified, age 2-16 yrs "Turkey"	Plasma B12, pg/ml and compare the mean (SD) between patients and controls	Mean (SD) =575 (430) pg /ml and 595 (405) pg /ml at 6 months later in patients versus 301 (81) pg/ml in the controls; p =<0.001	
18376090 Adiga et al., 2008 [88]	Plasma B12 and Pediatric acute Lymphoblastic Leukemia. 30 cases	Plasma B12	mean (SD) of p B12 = 274 (111) versus 297 (109)in the controls, p = 0.527	Age and sex matched controls

	with Pediatric acute Lymphoblastic Leukemia and 30 controls, "India"			
32050436 Urbanski et al., 2020 [89]	750 patients with cancer and normal S-B12 and 750 with cancer and high B12. A hospital based case-control study of patients with indication for B12 test	S-B12 >= 1000 ng/ml or below this cutoff.	OR (95%CI) for B12 > = 1000 ng/ml for Colon and rectum cancers = 3.02 [1.35–6.75]; Pancreas 4.00 [1.02–15.65]; Lungs 2.89 [1.14–7.35]; Prostate 2.17 [1.02–4.63]; Urothelium 7.40 [1.77–30.87].	Patients from the intensive care unit, hemodialysis, infection or metabolic changes? Were excluded. The controls were matched to the patients for the department, age and sex.
30642843 Arendt et al., 2019 [90] (cohort study)	P-B12 and cancer first diagnosis or death from cancer. Overall 33367 cancer cases (mixed cancers) identified (4% of all) out of 757185. "UK The health improvement network (THIN) primary care database" outcome is cancer first diagnosis or death from cancer	P-B12, pmol/L stratified Reference category is 150-600 pmol/L. tested groups;<br>601-800 vs. 150-600 pmol/L; 801- 1000 vs. 150-600 pmol/L; >1000 vs. 150-600 pmol/L<br>B12 below 150 pmol/L excluded	adjusted IRR and 95%CI<br>for B12 601-800 vs. 150-600 pmol/L overall; **1.31 (1.21-1.41); <1 yr 1.74 (1.54-1.96); 1-2yrs 1.39 (1.15-1.69).**<br>for B12 801- 1000 vs. 150-600 pmol/L overall; **1.88 (1.64-2.15); <1 yr 2.90 (2.39-3.51); 1-2yrs 1.39 (0.93-2.07)**.<br>for B12 >1000 vs. 150-600 pmol/L overall; 2**.24 (2.11-2.77); <1 yr 4.72 (3.99-5.58); 1-2yrs 1.58 (1.04-2.41)**	Adjusted for sex, age, alcohol, BMI, smoking
26724465 Arendt et al., 2016 [12]	S- B12 among 25017 patients overall cancer cases (mixed	S-B12 measured up to 1 year prior to diagnosis of cancer and 30 days	S-B12 601-800 pmol/L vs. the reference category mortality risk ratio and 95%CI	Adjusted for age, sex, calendar year, Charlson comorbidity score index, and

(cohort study)	population) identified in health register data from Denmark	mortality risk ratio. Reference group B12 200-600 pmol/L (B12 deficiency excluded)	=1.9 (1.6-2.2); for plasma B12 > 800 pmol/L, the mortality risk ratio and 95%CI =2.7 (2.4-3.1). In addition, 31-90d mortality, p value = 0.0003; 91-365d mortality p = 0.0179	cancer stage
33032595 Wolffenbuttel et al. 2020 [91] (cohort study)	3023 had cancers or died from cancer and 21239 (still alive). Based on the all NHANES surveys 1999–2000 through 2013–2014	Measured plasma B12 and B12 intake The outcome was cancer and cancer related mortality. pB12 < 140 pmol/L 140-300 pmol/L 300-700 pmol/L (Reference group) > 700 pmol/L (in the full cohort with available MMA). Also studied B12 intake from supplements : no B12 supplements (Reference group); ≤4.9 µg/d (all from suppl); 5.0-24.9 µg/d; 25-99.9g/d; 100-999 µg/d; > 999 µg/d	HR and 95%CI for (cancer mortality) P- B12 < 140 pmol/L vs. 300-700 pmol/L = 1.65 (0.93-2.93); B12 140-300 pmol/L vs. 300-700 pmol/L = **1.38 (1.01-1.87);** > 700 vs. 300-700 pmol/L = 1.28 (0.83-1.98). HR and 95%CI for (cancer mortality) B12 from supplements (vs. no supplements as a reference group); data shown only as Figures: significantly lower cancer mortality in the ≤4.9 µg/d and the 100-999 µg/d intake groups versus the no supplement group in the fully adjusted model; otherwise, the associations were generally inconsistent and did not show dose-response associations.	adjusted for age; gender; ethnicity; BMI group; family income; education; former and current smoking; alcohol consumption; eGFR < 60 ml/min/1.73m2; diagnosis of diabetes, hypertension, cardiovascular disease, cancer, and lung disease; medication use (as a proxy for other comorbidities); white blood cell count; hemoglobin; and serum folate
33455102 Djurovic et al., 2020 [92]	27 Patients with malignant brain tumor who	P-B12 measured preoperative, and at 1, 3, 6 months after the	Plasma B12 did not differ between patients who developed relapse compared to those who	

(cohort study)	developed relapse over the follow up time	operation	remained free of relapse.	
24249744 Arendt et al., 2013 [93] (cohort study)	P-B12 among 333667 persons (22652 incident cancers) mixed cancers (hematology, immune related, hormone related, lifestyle related) Data source Health register study, Denmark	the incidence of cancer diagnoses among patients with elevated P-B12 concentrations. SIR, standardized incidence ratio (SIR; ie, the ratio of observed cancers to expected cancers). Expected cancers were calculated by multiplying person-years of follow-up by the population-based cancer incidence rates	Overall SIR and B12: 200-600 pmol/L = 1.23 (1.21-1.24). Overall SIR and B12: 601-800 pmol/L = 1.61 (1.51 to 1.71). Overall SIR and B12: > 800 pmol/L = 2.38 (2.22 to 2.56).	In patients got diagnosed with cancer and had B12 measured in the last year pre diagnosis, 6.6 % had B12 > 800 pmol/L; those who got diagnosed after more than 1 year 4,4% had B12 > 800 pmol/L.
25985325 Zhang et al., 2015 [94]	Meta-Analysis of 83 Case-Control Studies on plasma B12 and several cancers in different populations	S-B12 concentrations	vitamin B12 concentrations were inversely associated with cancer risk for only urinary-system tumors (OR= -10.71; 95% CI -16.36 to -5.05) and digestive-system carcinomas (-31.14; -49.13 to -13.15) versus the controls.	urinary-system and gastrointestinal carcinomas and for Asian and Middle Eastern patients

Table S11. Studies on genetic factors and **mendelian randomization**		
Reference (PMID author year citation)	High B12 vs. reference category	Effect size
Collin et al, 2010 [53] 20852008	424 men followed for 2.5 years. Outcome PSA velocity > 2 ng/ml/y (a proxy measure of progression of localized **prostate cancer**), "UK"	Polymorphisms related to B12<br>The outcome was analyzed as a continuous and as categorical variable.<br>MTR 2756A>G (rs1805087)<br>MTRR 66A>G (rs1801394)<br>TCN2 776C>G (rs1801198)<br>TCN1 372 T>C (rs526934)<br>CUBN 758C>T (rs 1801222)<br>MUT 1595 G>A (rs1141321).<br>**MTRR 66A>G** (rs1801394) recessive allele OR for PSA > 2 = 0.33 (0.11-0.97).<br>All other genotype did not show significant associations
17119065 Semmler, et al., 2006 [95]	328 patients with glioblastoma multiforme and 400 controls. Outcome glioblastoma multiforme, "Germany"	MTR c.2756 A>G<br>Tc2 c.776C>G.<br>The MTR c.2756AG and GG allelotypes) was significantly less frequently found among glioblastoma multiforme patients when compared with controls (28% versus 43%; odds ratio, 0.52; 95% confidence interval, 0.37-0.71; Chi2 = 17.4; P = 0.00003.<br>for Tc2 c.776C>G Chi2 = 0.18; P = 0.912
20237949 Kurzwelly et al., 2010 [96]	185 immunocompetent patients and 212 controls. Primary central nervous system lymphoma (PCNSL)	MTR c.2756A>G (D919G), Pearson chi2 analysis: Chi 2= 4.17, p = 0.125. The MTR c.2756A [G polymorphism (G-allele) was less frequent in PCNSL patients than in controls: OR (95%CI) = 0.65 (0.43–0.99).<br>Tc2 c.776C>G Pearson chi2 analysis: Chi 2= 2.17, p = 0.338. MTR c.2756A>G (D919G), Pearson chi2 analysis: Chi 2= 4.17, p = 0.125. The MTR c.2756A [G polymorphism (G-allele) was less frequent in PCNSL patients than in controls: OR (95%CI) = 0.65 (0.43–0.99).<br>Tc2 c.776C>G Pearson chi2 analysis: Chi 2= 2.17, p = 0.338.
19389261 Ma et al., 2009	458 age-matched pairs of Brazilian women with breast	MTR AG vs. AA OR (95%CI) = 0.81 (0.59–1.10);<br>GG vs. AA = 1.99 (1.01–3.92).

[29]	cancer. MTR AA (Ref), AG, GG	The GG genotype is associated with a higher risk of breast cancer (vs. AA). Adjusted for smoking status (never/ever), alcohol consumption (never/ever), moderate physical activity in the preceding 5 years (no/yes), and number of live births (nulliparous/1–2/≥3)
32770085 Zhang et al., 2020 [97]	Methionine synthase expression and the overall survival time. Totally, 10,666 prostate cancer patients and 40,750 controls were included. Methionine synthase rs1805087 A/G variant	Methionine synthase rs1805087 A/G variant is associated with an elevated risk of prostate cancer (G-allele vs. A-allele: OR = 1.06, 95% CI = 1.01-1.11, heterozygous model: OR = 1.08, 95% CI = 1.02-1.14; dominant model: OR = 1.08, 95% CI = 1.02-1.14).<br>Methionine synthase rs1805087 A/G variant may be associated with susceptibility of prostate cancer, especially in Asian populations, hospital-based studies and that with high quality and large sample size. Furthermore, Methionine synthase rs1805087 A/G variant may be related to the prognosis of prostate cancer.
32064992 Gohari et al., 2021, [98] Meta-analysis	Three studies with 283 cases with **retinoblastoma** and 485 controls on MTR 2756 A > G (all studies on children). Included 20310006 de lima et al., 2010; 26595280 Akbari et al., 2015; and 31610671 Gohari et al., 2019	The pooled estimate OR = 3.51 (1.52-8.07) fixed effect model for MTR GG versus (AA+AG)
32722923 Bai et al., 2020 [99] (only abstract available)	MTR A2756G polymorphism and hematological malignancies	lack of association between the risk of hematological malignancies and MTR A2756G polymorphism under the allele model (G vs A: odds ratio = 1.001, 95% CI: 0.944–1.061; p = 0.983), recessive model (GG vs GA + AA: odds ratio = 1.050, 95% CI: 0.942–1.170; p = 0.382)
30559146 Ma et al., 2019 [100] Meta-analysis	*MTR* A2756G polymorphism and pediatric ALL risk. Included ten available studies with 3224 ALL cases and 4077 matched controls	AG vs. AA: OR = 1.13, 95%CI = 1.02-1.26, *P* = 0.02; AG+GG vs. AA: OR = 1.13, 95%CI = 1.02-1.25, *P* = 0.01; G allele vs. A allele: OR = 1.10, 95%CI = 1.01-1.20, *P* = 0.03

20111902 Lu et al., 2010 [101](Meta-analysis)	A2756G polymorphism in the methionine synthase (MTR) and breast cancer risk. Eleven published case-control studies, including 8,438 breast cancer cases and 10,515 controls were identified	No significant associations between the MTR A2756G polymorphism and breast cancer risk were found for GG versus AA (OR = 0.98, 95% CI: 0.84-1.15), AG versus AA (OR = 0.95, 95% CI: 0.89-1.01), GG/AG versus AA (OR = 0.95, 95% CI = 0.89-1.01), and GG versus AG/AA (OR = 1.00, 95% CI: 0.86-1.17). However, in the stratified analysis, significantly decreased breast cancer risks were found among Europeans (AG versus AA, OR = 0.90, 95% CI = 0.83-0.98; GG/AG versus AA, OR = 0.90, 95% CI = 0.82-0.97) and studies with population-based controls (AG versus AA, OR = 0.93, 95% CI = 0.86-1.00; GG/AG versus AA, OR = 0.93, 95% CI = 0.86-1.00).
19826453 Yu et al., 2010 [102] (Meta-analysis)	A total of 24 896 cancer patients and 33 862 controls from 52 articles for *MTR A2756G* were investigated.	Carrying *MTR* 2756GG genotype had a subtly reduced cancer risk under a recessive genetic model (odds ratio (OR), 0.92; 0.84-1.00. In the subgroup analyses by ethnicity, 2756GG was associated with a significantly reduced cancer risk in European populations (OR, 0.83, 0.74–0.93. However, in Asian populations, a significant association between 2756GG genotype and cancer risk was observed (OR, 1.33, 1.06–1.65). There was significantly lower risk of acute lymphoblastic leukemia OR, 0.54, 0.29- 1.00) and colorectal cancer (OR, 0.63, 0.47–0.87) in European populations.
23845785 Zhong et al., 2013 [103](Meta-analysis)	Methionine synthase A2756G polymorphism and breast cancer risk. 16 available studies with 9866 cases and 11,702 controls estimating the association between MTR A2756G and breast cancer risk was conducted.	The 2756G allele was associated with a decreased risk in Caucasians, PB (population-based) subgroup, and large studies. But the associations disappeared after removing the studies not in HWE. On the contrary, an increased risk was found in small studies. In conclusion, the findings suggest that MTR A2756G polymorphism is not associated with altered susceptibility to breast cancer, while the observed decreased risk in Caucasians, PB subgroup, and large studies and increased risk in small studies may be due to selection bias or other unknown factor
32355161 Guo et al., 2020 [104] Mendelian randomization	25509 cases with epithelial ovarian cancers and 40941 controls	Genetically predicted vitamin B12 concentration was not significantly associated with low malignant epithelial ovarian cancers in the IVW method (OR, 1.16; 95% CI, 0.96–1.41)- only after one outlier was removed
18447718 Semmler et al.,	290 patients and 287 controls Grade I-III meningioma MTR	Prevalence is not significantly different between patients and controls (However all patients with Grade III had AA genotype)

2008 [105]	c.2756A>G	
33740060 Tsilidis et al., 2021 [106] "Mendelian randomization"	126,000 participants of European ancestry from the GECCO, CORECT, and CCFR consortia provided the genetic effects of the selected instruments on risk of colorectal (58,221 cases and 67,694 controls), colon (31,083 cases), rectal (15,775 cases), proximal colon (13,857 cases), and distal colon (15,306 cases) cancer	rs602662 and rs1801222 associated with vitamin B-12, A 2-sampleMR using summary association data from GWASs of circulating micronutrients (first sample) and colorectal cancer risk (second sample).<br>1-SD (173 pmol/L) increase in the genetically predicted concentration of vitamin B-12 was associated with a 12% OR: 1.12 (1.04, 1.19), 10% OR 1.10 (1.02,1.19), and 21% OR 1.21 (1.09, 1.34) higher risk of colorectal, colon, and rectal cancer, respectively, but not cancer in other subsites.
33837300 Yuan S, et al., 2021 [107]	A UK Biobank-based study including 367,561 individuals and FinnGen consortium comprising up to 176,899 participants. (45,576 individuals of European ancestries) for B12)	Average concentrations 391 pmol/L for vitamin B12.<br>Genetically predicted high serum B12 was associated with an elevated risk of colorectal cancer in UK Biobank OR 1.16 (1.06, 1.26) and both the direction and magnitude of the association remained in FinnGen (OR 1.19 (0.99, 1.43). The combined OR of colorectal cancer was 1.16 (1.08, 1.25). Did not detect any SNPs driving the associations of vitamin B12 with overall digestive system cancer or colorectal cancer. Genetically predicted serum vitamin B12 was not associated with overall cancer, or oesophageal, gastric or pancreatic cancer risk
35046699 Wang et al., 2022 [108]	MTR rs1805087 A>G and breast cancer in Chinese women	Several genotypes related to C1 metabolism. Only MTR is relevant to B12.<br>GA/GG vs. AA<br>Adj OR (95%CI) =1.33 (0.93–1.89); Additive model<br>Adjusted OR = 1.29 (0.92–1.79). Adjusted for age and menopausal status

Table S12. **Randomized controlled trials** on intervention including vitamin B12 and cancer or cancer-related outcomes.

Reference (PMID author year citation)	High B12 vs. reference category	Effect size
30341095 Araghi et al., 2019 [109]	Long-term follow-up of B-PROOF trial participants (N ¼ 2,524), a multicenter, double-blind randomized placebo-controlled trial designed to assess the effect of 2 to 3 years daily supplementation with folic acid (400 mg) and vitamin B12 (500 mg) versus placebo on fracture incidence. median follow up of 78 months; IQR: 74–83	Allocation to B vitamins was associated with a higher risk of overall cancer [171 (13.6%) vs. 143 (11.3%); HR 1.25 (1.00–1.53). B vitamins were significantly associated with a higher risk of colorectal cancer [43(3.4%) vs. 25(2.0%); HR 1.77 (1.08–2.90).<br>This positive finding might be related to an altered vitamin B12 metabolism caused by carcinogenesis prior to clinical cancer diagnosis. Possible bias because the allocation to the intervention and control group was no longer blinded to the researchers. Secondary analyses of a randomized controlled trial primarily designed to study the effect on fracture risk
18984888 Zhang, 2008 [110]	the Women's Antioxidant and Folic Acid Cardiovascular Study, 5442 US female health professionals aged 42 years or older, with preexisting cardiovascular disease or 3 or more coronary risk factors, were randomly assigned to receive either a daily combination of folic acid, vitamin B(6), and vitamin B(12) or a matching placebo. They were treated for 7.3 years from April 1998 through July 31, 2005.<br>Daily supplementation of a combination of 2.5 mg of folic acid, 50 mg of vitamin B(6), and 1 mg of vitamin B(12) (n = 2721) or placebo (n = 2721).	379 women developed invasive cancer (187 in the active treatment group and 192 in the placebo). The active treatment had similar risk of developing total invasive cancer vs. placebo (101.1/10,000 PY for the active treatment group vs 104.3/10,000 py for placebo group; hazard ratio [HR], 0.97; 95% CI], 0.79-1.18; P = .75), breast cancer (37.8/10,000 PY vs 45.6/10,000 PY, respectively; HR, 0.83; 95% CI, 0.60-1.14; P = .24), or any cancer death (24.6/10,000 PY vs 30.1/10,000 PY, respectively; HR, 0.82; 95% CI, 0.56-1.21; P = .32).

22474057 Hankey, et al 2012 [111]	8164 patients (B Vitamin n= 4089, Placebo n = 4075) with recent stroke or transient ischemic attack were randomly allocated to double-blind treatment with 1 tablet daily of placebo or B vitamins (2 mg folic acid, 25 mg vitamin B(6), 500 µg vitamin B(12)) and followed for a median of 3.4 years for any cancer as an adverse event.	no significant difference in the incidence of any cancer among participants assigned B vitamins compared with placebo (4.04% versus 4.59%; risk ratio, 0.86; 95% CI, 0.70-1.07) and no difference in cancer mortality (2.35% versus 2.09%; risk ratio, 1.09; 0.81-1.46). Among 1899 patients with diabetes, the incidence of cancer was higher among participants assigned B vitamins compared with placebo (5.35% versus 3.28%; adjusted risk ratio, 2.21; 1.31-3.73), whereas among 6168 patients without diabetes, the incidence of cancer was lower among participants assigned B vitamins compared with placebo (3.66% versus 5.03%; adjusted risk ratio, 0.67; 0.51-0.87; P for interaction=0.0001).
23066166 Song et al., 2012 [112]	The Women's Antioxidant and Folic Acid Cardiovascular Study was a randomized, double-blind, placebo-controlled trial of 5442 female health professionals at high risk for cardiovascular disease from April 1998 through July 2005. Participants were randomly assigned to receive a combination pill of folic acid (2.5mg), vitamin B6 (50mg), and vitamin B12 (1mg) or placebo. This study included 1470 participants who were followed up for as long as 9.2 years and underwent an endoscopy at any point during follow-up	The risk of colorectal adenoma was similar among participants receiving treatment (24.3%, 180 of 741 participants) vs placebo (24.0%, 175 of 729 participants) (multivariable adjusted relative risk = 1.00, 95% confidence interval = 0.83 to 1.20).<br>Treatment was not associated with the risk of adenoma when data were analyzed by subsite, size, stage, and the number of adenomas. There was no statistically significant effect modification by alcohol intake, history of cancer or adenoma, or baseline plasma concentrations or intakes of folate, vitamin B6, or vitamin B12.
19920236 Ebbing, 2009 [113]	combined analyses and extended follow up from 2 RCTs Norwegian Vitamin Trial and Western Norway B Vitamin Intervention Trial (WENBIT and NORVIT trials).<br>folic acid 0.8 mg + B12 0.4 mg+ B6 40 mg or folic acid 0.8 mg+ B12 0.4 mg or B6 40 mg or placebo	cancer incidence in folic acid vs. Non-folic acid group HR = **1.21 (1.03-1.41);** cancer mortality HR = **1.38 (1.07-1.79)**

20937919 Clark et al., 2010 [114] (Meta-analysis)	Meta-analysis of 8 large, randomized, placebo-controlled trials of folic acid supplementation involving 37 485 individuals at increased risk of cardiovascular disease. There were 9326 major vascular events (3990 major coronary events, 1528 strokes, and 5068 revascularizations), 3010 cancers, and 5125 deaths.	There was no significant effect on the rate ratios (95% confidence intervals) for overall cancer incidence (1.05 [0.98-1.13]), cancer mortality (1.00 [0.85-1.18]) or all-cause mortality (1.02 [0.97-1.08]) during the whole scheduled treatment period or during the later years of it.

Reference List

1. Pan D, Wang S, Su M, Sun G, Zhu X, Ghahvechi CM, Guo Z, Wang N, Zhang Z, Cui M (2021) Vitamin B12 may play a preventive role in esophageal precancerous lesions: a case-control study based on markers in blood and 3-day duplicate diet samples. Eur J Nutr 60:3375-3386.

2. Chang SC, Goldstein BY, Mu L, Cai L, You NC, He N, Ding BG, Zhao JK, Yu SZ, Heber D et al. (2015) Plasma folate, vitamin B12, and homocysteine and cancers of the esophagus, stomach, and liver in a Chinese population. Nutr Cancer 67:212-223.

3. Ren J, Murphy G, Fan J, Dawsey SM, Taylor PR, Selhub J, Qiao Y, Abnet CC (2016) Prospective study of serum B vitamins levels and oesophageal and gastric cancers in China. Sci Rep 6:35281.

4. Miranti EH, Stolzenberg-Solomon R, Weinstein SJ, Selhub J, Mannisto S, Taylor PR, Freedman ND, Albanes D, Abnet CC, Murphy G (2017) Low vitamin B12 increases risk of gastric cancer: A prospective study of one-carbon metabolism nutrients and risk of upper gastrointestinal tract cancer. Int J Cancer 141:1120-1129.

5. Vollset SE, Igland J, Jenab M, Fredriksen A, Meyer K, Eussen S, Gjessing HK, Ueland PM, Pera G, Sala N et al. (2007) The association of gastric cancer risk with plasma folate, cobalamin, and methylenetetrahydrofolate reductase polymorphisms in the European Prospective Investigation into Cancer and Nutrition. Cancer Epidemiol Biomarkers Prev 16:2416-2424.

6. Jessri M, Rashidkhani B, Hajizadeh B, Jessri M, Gotay C (2011) Macronutrients, vitamins and minerals intake and risk of esophageal squamous cell carcinoma: a case-control study in Iran. Nutr J 10:137.

7. Sharp L, Carsin AE, Cantwell MM, Anderson LA, Murray LJ (2013) Intakes of dietary folate and other B vitamins are associated with risks of esophageal adenocarcinoma, Barrett's esophagus, and reflux esophagitis. J Nutr 143:1966-1973.

8. Ibiebele TI, Hughes MC, Pandeya N, Zhao Z, Montgomery G, Hayward N, Green AC, Whiteman DC, Webb PM (2011) High intake of folate from food sources is associated with reduced risk of esophageal cancer in an Australian population. J Nutr 141:274-283.

9. Xiao Q, Freedman ND, Ren J, Hollenbeck AR, Abnet CC, Park Y (2014) Intakes of folate, methionine, vitamin B6, and vitamin B12 with risk of esophageal and gastric cancer in a large cohort study. Br J Cancer 110:1328-1333.

10. Mayne ST, Risch HA, Dubrow R, Chow WH, Gammon MD, Vaughan TL, Farrow DC, Schoenberg JB, Stanford JL, Ahsan H et al. (2001) Nutrient intake and risk of subtypes of esophageal and gastric cancer. Cancer Epidemiol Biomarkers Prev 10:1055-1062.

11. Khairan P, Sobue T, Eshak ES, Zha L, Kitamura T, Sawada N, Iwasaki M, Inoue M, Yamaji T, Shimazu T et al. (2021) Association of dietary intakes of vitamin B12, vitamin B6, folate, and methionine with the risk of esophageal cancer: the Japan Public Health Center-based (JPHC) prospective study. BMC Cancer 21:982.

12. Arendt JF, Farkas DK, Pedersen L, Nexo E, Sorensen HT (2016) Elevated plasma vitamin B12 levels and cancer prognosis: A population-based cohort study. Cancer Epidemiol 40:158-165.

13. Qiang Y, Li Q, Xin Y, Fang X, Tian Y, Ma J, Wang J, Wang Q, Zhang R, Wang J et al. (2018) Intake of Dietary One-Carbon Metabolism-Related B Vitamins and the Risk of Esophageal Cancer: A Dose-Response Meta-Analysis. Nutrients 10.

14. He J, Fu H, Li C, Deng Z, Chang H (2022) Association between Vitamin B12 and Risk of Gastric Cancer: A Systematic Review and Meta-Analysis of Epidemiological Studies. Nutr Cancer 1-11.

15. Jansen RJ, Robinson DP, Frank RD, Anderson KE, Bamlet WR, Oberg AL, Rabe KG, Olson JE, Sinha R, Petersen GM et al. (2014) Fatty acids found in dairy, protein and unsaturated fatty acids are associated with risk of pancreatic cancer in a case-control study. Int J Cancer 134:1935-1946.

16. Chuang SC, Stolzenberg-Solomon R, Ueland PM, Vollset SE, Middtun O, Olsen A, Tjonneland A, Overvad K, Boutron-Ruault MC, Morois S et al. (2011) A U-shaped relationship between plasma folate and pancreatic cancer risk in the European Prospective Investigation into Cancer and Nutrition. Eur J Cancer .

17. Schernhammer E, Wolpin B, Rifai N, Cochrane B, Manson JA, Ma J, Giovannucci E, Thomson C, Stampfer MJ, Fuchs C (2007) Plasma folate, vitamin B6, vitamin B12, and homocysteine and pancreatic cancer risk in four large cohorts. Cancer Res 67:5553-5560.

18. Gong Z, Holly EA, Bracci PM (2009) Intake of folate, vitamins B6, B12 and methionine and risk of pancreatic cancer in a large population-based case-control study. Cancer Causes Control 20:1317-1325.

19. Stolzenberg-Solomon RZ, Albanes D, Nieto FJ, Hartman TJ, Tangrea JA, Rautalahti M, Sehlub J, Virtamo J, Taylor PR (1999) Pancreatic cancer risk and nutrition-related methyl-group availability indicators in male smokers. J Natl Cancer Inst 91:535-541.

20. Marley AR, Fan H, Hoyt ML, Anderson KE, Zhang J (2018) Intake of methyl-related nutrients and risk of pancreatic cancer in a population-based case-control study in Minnesota. Eur J Clin Nutr 72:1128-1135.

21. Huang JY, Butler LM, Wang R, Jin A, Koh WP, Yuan JM (2016) Dietary Intake of One-Carbon Metabolism-Related Nutrients and Pancreatic Cancer Risk: The Singapore Chinese Health Study. Cancer Epidemiol Biomarkers Prev 25:417-424.

22. Stolzenberg-Solomon RZ, Pietinen P, Barrett MJ, Taylor PR, Virtamo J, Albanes D (2001) Dietary and other methyl-group availability factors and pancreatic cancer risk in a cohort of male smokers. Am J Epidemiol 153:680-687.

23. Wei DH, Mao QQ (2020) Vitamin B6, vitamin B12 and methionine and risk of pancreatic cancer: a meta-analysis. Nutr J 19:111.

24. Hatami M, Vahid F, Esmaeil AM, Sadeghi M, Ameri F, Eini-Zeinab H, Jamshidi-Naeini Y, Hossein DS (2020) The Vitamins Involved in One-Carbon Metabolisms are Associated with Reduced Risk of Breast Cancer in Overall and Subtypes. Int J Vitam Nutr Res 90:131-140.

25. Agnoli C, Grioni S, Krogh V, Pala V, Allione A, Matullo G, Di GC, Tagliabue G, Pedraglio S, Garrone G et al. (2016) Plasma Riboflavin and Vitamin B-6, but Not Homocysteine, Folate, or Vitamin B-12, Are Inversely Associated with Breast Cancer Risk in the European Prospective Investigation into Cancer and Nutrition-Varese Cohort. J Nutr 146:1227-1234.

26. Matejcic M, de BJ, Ricci C, Biessy C, Perrier F, Huybrechts I, Weiderpass E, Boutron-Ruault MC, Cadeau C, His M et al. (2017) Biomarkers of folate and vitamin B12 and breast cancer risk: report from the EPIC cohort. Int J Cancer 140:1246-1259.

27. Lin J, Lee IM, Cook NR, Selhub J, Manson JE, Buring JE, Zhang SM (2008) Plasma folate, vitamin B-6, vitamin B-12, and risk of breast cancer in women. Am J Clin Nutr 87:734-743.

28. Houghton SC, Eliassen AH, Zhang SM, Selhub J, Rosner BA, Willett WC, Hankinson SE (2019) Plasma B-vitamin and one-carbon metabolites and risk of breast cancer before and after folic acid fortification in the United States. Int J Cancer 144:1929-1940.

29. Ma E, Iwasaki M, Junko I, Hamada GS, Nishimoto IN, Carvalho SM, Motola J, Jr., Laginha FM, Tsugane S (2009) Dietary intake of folate, vitamin B6, and vitamin B12, genetic polymorphism of related enzymes, and risk of breast cancer: a case-control study in Brazilian women. BMC Cancer 9:122.

30. Ma E, Iwasaki M, Kobayashi M, Kasuga Y, Yokoyama S, Onuma H, Nishimura H, Kusama R, Tsugane S (2009) Dietary intake of folate, vitamin B2, vitamin B6, vitamin B12, genetic polymorphism of related enzymes, and risk of breast cancer: a case-control study in Japan. Nutr Cancer 61:447-456.

31. Maruti SS, Ulrich CM, White E (2009) Folate and one-carbon metabolism nutrients from supplements and diet in relation to breast cancer risk. Am J Clin Nutr 89:624-633.

32. Yang D, Baumgartner RN, Slattery ML, Wang C, Giuliano AR, Murtaugh MA, Risendal BC, Byers T, Baumgartner KB (2013) Dietary intake of folate, B-vitamins and methionine

and breast cancer risk among Hispanic and non-Hispanic white women. PLoS ONE 8:e54495.

33. Chou YC, Chu CH, Wu MH, Hsu GC, Yang T, Chou WY, Huang HP, Lee MS, Yu CP, Yu JC et al. (2011) Dietary intake of vitamin B(6) and risk of breast cancer in Taiwanese women. J Epidemiol 21:329-336.

34. Xu HL, Gong TT, Liu FH, Wei YF, Chen HY, Yan S, Zhao YH, Gao S, Jiao YS, Wu QJ (2022) Pre-diagnosis Dietary One-Carbon Metabolism Micronutrients Consumption and Ovarian Cancer Survival: A Prospective Cohort Study. Front Nutr 9:873249.

35. Kim SJ, Zuchniak A, Sohn KJ, Lubinski J, Demsky R, Eisen A, Akbari MR, Kim YI, Narod SA, Kotsopoulos J (2016) Plasma folate, vitamin B-6, and vitamin B-12 and breast cancer risk in B. Am J Clin Nutr 104:671-677.

36. Essen A, Santaolalla A, Garmo H, Hammar N, Walldius G, Jungner I, Malmstrom H, Holmberg L, Van HM (2019) Baseline serum folate, vitamin B12 and the risk of prostate and breast cancer using data from the Swedish AMORIS cohort. Cancer Causes Control 30:603-615.

37. Bassett JK, Baglietto L, Hodge AM, Severi G, Hopper JL, English DR, Giles GG (2013) Dietary intake of B vitamins and methionine and breast cancer risk. Cancer Causes Control 24:1555-1563.

38. Stevens VL, McCullough ML, Sun J, Gapstur SM (2010) Folate and other one-carbon metabolism-related nutrients and risk of postmenopausal breast cancer in the Cancer Prevention Study II Nutrition Cohort. Am J Clin Nutr 91:1708-1715.

39. Shrubsole MJ, Shu XO, Li HL, Cai H, Yang G, Gao YT, Gao J, Zheng W (2011) Dietary B vitamin and methionine intakes and breast cancer risk among Chinese women. Am J Epidemiol 173:1171-1182.

40. Zhang CX, Ho SC, Chen YM, Lin FY, Fu JH, Cheng SZ (2011) Dietary folate, vitamin B6, vitamin B12 and methionine intake and the risk of breast cancer by oestrogen and progesterone receptor status. Br J Nutr 106:936-943.

41. Wu W, Kang S, Zhang D (2013) Association of vitamin B6, vitamin B12 and methionine with risk of breast cancer: a dose-response meta-analysis. Br J Cancer 109:1926-1944.

42. Pathak S, Bajpai D, Banerjee A, Bhatla N, Jain SK, Jayaram HN, Singh N (2014) Serum one-carbon metabolites and risk of cervical cancer. Nutr Cancer 66:818-824.

43. Ragasudha PN, Thulaseedharan JV, Wesley R, Jayaprakash PG, Lalitha P, Pillai MR (2012) A case-control nutrigenomic study on the synergistic activity of folate and vitamin B12 in cervical cancer progression. Nutr Cancer 64:550-558.

44. Kwanbunjan K, Saengkar P, Cheeramakara C, Tangjitgamol S, Chitcharoenrung K (2006) Vitamin B12 status of Thai women with neoplasia of the cervix uteri. Southeast Asian J Trop Med Public Health 37 Suppl 3:178-183.

45. Piyathilake CJ, Macaluso M, Alvarez RD, Bell WC, Heimburger DC, Partridge EE (2009) Lower risk of cervical intraepithelial neoplasia in women with high plasma folate and sufficient vitamin B12 in the post-folic acid fortification era. Cancer Prev Res (Phila) 2:658-664.

46. Tong SY, Kim MK, Lee JK, Lee JM, Choi SW, Friso S, Song ES, Lee KB, Lee JP (2011) Common polymorphisms in methylenetetrahydrofolate reductase gene are associated with risks of cervical intraepithelial neoplasia and cervical cancer in women with low serum folate and vitamin B12. Cancer Causes Control 22:63-72.

47. Vidal AC, Grant DJ, Williams CD, Masko E, Allott EH, Shuler K, McPhail M, Gaines A, Calloway E, Gerber L et al. (2012) Associations between Intake of Folate, Methionine, and Vitamins B-12, B-6 and Prostate Cancer Risk in American Veterans. J Cancer Epidemiol 2012:957467.

48. de Vogel S, Meyer K, Fredriksen A, Ulvik A, Ueland PM, Nygard O, Vollset SE, Tell GS, Tretli S, Bjorge T (2013) Serum folate and vitamin B12 concentrations in relation to prostate cancer risk--a Norwegian population-based nested case-control study of 3000 cases and 3000 controls within the JANUS cohort. Int J Epidemiol 42:201-210.

49. Price AJ, Travis RC, Appleby PN, Albanes D, Barricarte GA, Bjorge T, Bueno-de-Mesquita HB, Chen C, Donovan J, Gislefoss R et al. (2016) Circulating Folate and Vitamin B and Risk of Prostate Cancer: A Collaborative Analysis of Individual Participant Data from Six Cohorts Including 6875 Cases and 8104 Controls. Eur Urol .

50. Collin SM (2013) Folate and B12 in prostate cancer. Adv Clin Chem 60:1-63.

51. Hultdin J, Van GB, Bergh A, Hallmans G, Stattin P (2005) Plasma folate, vitamin B12, and homocysteine and prostate cancer risk: a prospective study. Int J Cancer 113:819-824.

52. Johansson M, Appleby PN, Allen NE, Travis RC, Roddam AW, Egevad L, Jenab M, Rinaldi S, Kiemeney LA, Bueno-de-Mesquita HB et al. (2008) Circulating concentrations of folate and vitamin B12 in relation to prostate cancer risk: results from the European Prospective Investigation into Cancer and Nutrition study. Cancer Epidemiol Biomarkers Prev 17:279-285.

53. Collin SM, Metcalfe C, Refsum H, Lewis SJ, Smith GD, Cox A, Davis M, Marsden G, Johnston C, Lane JA et al. (2010) Associations of folate, vitamin B12, homocysteine, and folate-pathway polymorphisms with prostate-specific antigen velocity in men with localized prostate cancer. Cancer Epidemiol Biomarkers Prev 19:2833-2838.

54. Boot IWA, Wesselius A, Yu EYW, Brinkman M, van den Brandt P, Grant EJ, White E, Weiderpass E, Ferrari P, Schulze MB et al. (2022) Dietary B group vitamin intake and the bladder cancer risk: a pooled analysis of prospective cohort studies. Eur J Nutr 61:2397-2416.

55. Ben Fradj MK, Mrad DK, Kallel A, Bibi M, Ben RS, Sanhaji H, Nouira Y, Feki M (2019) Interaction Effects of Plasma Vitamins A, E, D, B9, and B12 and Tobacco Exposure in Urothelial Bladder Cancer: A Multifactor Dimensionality Reduction Analysis. Nutr Cancer 71:1382-1389.

56. Vrieling A, Bueno-de-Mesquita HB, Ros MM, Kampman E, Aben KK, Buchner FL, Jansen EH, Roswall N, Tjonneland A, Boutron-Ruault MC et al. (2019) One-carbon metabolism biomarkers and risk of urothelial cell carcinoma in the European prospective investigation into cancer and nutrition. Int J Cancer 145:2349-2359.

57. Johansson M, Fanidi A, Muller DC, Bassett JK, Midttun O, Vollset SE, Travis RC, Palli D, Mattiello A, Sieri S et al. (2014) Circulating biomarkers of one-carbon metabolism in relation to renal cell carcinoma incidence and survival. J Natl Cancer Inst 106.

58. Gibson TM, Weinstein SJ, Mayne ST, Pfeiffer RM, Selhub J, Taylor PR, Virtamo J, Albanes D, Stolzenberg-Solomon R (2010) A prospective study of one-carbon metabolism biomarkers and risk of renal cell carcinoma. Cancer Causes Control 21:1061-1069.

59. Bock CH, Ruterbusch JJ, Holowatyj AN, Steck SE, Van Dyke AL, Ho WJ, Cote ML, Hofmann JN, Davis F, Graubard BI et al. (2018) Renal cell carcinoma risk associated with lower intake of micronutrients. Cancer Med 7:4087-4097.

60. Ben Fradj MK, Ouanes Y, Hadj-Taeib S, Mrad DK, Bibi M, Jmal K, Sanhaji H, Nouira Y, Feki M (2021) Prognostic Significance of Plasma Folate and Cobalamin Concentrations in Non-Muscle-Invasive Bladder Cancer: A Prospective Cohort Study. Cancer Invest 39:240-250.

61. Cho E, Giovannucci EL, Joh HK (2013) Nutrients related to one-carbon metabolism and risk of renal cell cancer. Cancer Causes Control 24:373-382.

62. Clasen JL, Heath AK, Scelo G, Muller DC (2020) Components of one-carbon metabolism and renal cell carcinoma: a systematic review and meta-analysis. Eur J Nutr 59:3801-3813.

63. Dahlin AM, Van GB, Hultdin J, Johansson I, Hallmans G, Palmqvist R (2008) Plasma vitamin B12 concentrations and the risk of colorectal cancer: a nested case-referent study. Int J Cancer 122:2057-2061.

64. Ghorbani M, Azghandi M, Khayami R, Baharara J, Kerachian MA (2021) Association of MTHFR C677T variant genotype with serum folate and Vit B12 in Iranian patients with colorectal cancer or adenomatous polyps. BMC Med Genomics 14:246.

65. Liu GJ, Wang YJ, Yue M, Zhao LM, Guo YD, Liu YP, Yang HC, Liu F, Zhang X, Zhi LH et al. (2020) High expression of TCN1 is a negative prognostic biomarker and can predict neoadjuvant chemosensitivity of colon cancer. Sci Rep 10:11951.

66. Cheng TY, Makar KW, Neuhouser ML, Miller JW, Song X, Brown EC, Beresford SA, Zheng Y, Poole EM, Galbraith RL et al. (2015) Folate-mediated one-carbon metabolism genes and interactions with nutritional factors on colorectal cancer risk: Women's Health Initiative Observational Study. Cancer 121:3684-3691.

67. Eussen SJ, Vollset SE, Hustad S, Midttun O, Meyer K, Fredriksen A, Ueland PM, Jenab M, Slimani N, Boffetta P et al. (2010) Plasma vitamins B2, B6, and B12, and related genetic variants as predictors of colorectal cancer risk. Cancer Epidemiol Biomarkers Prev 19:2549-2561.

68. Bystrom P, Bjorkegren K, Larsson A, Johansson L, Berglund A (2009) Serum vitamin B12 and folate status among patients with chemotherapy treatment for advanced colorectal cancer. Ups J Med Sci 114:160-164.

69. Lee JE, Li H, Giovannucci E, Lee IM, Selhub J, Stampfer M, Ma J (2009) Prospective study of plasma vitamin B6 and risk of colorectal cancer in men. Cancer Epidemiol Biomarkers Prev 18:1197-1202.

70. Al-Ghnaniem R, Peters J, Foresti R, Heaton N, Pufulete M (2007) Methylation of estrogen receptor alpha and mutL homolog 1 in normal colonic mucosa: association with folate and vitamin B-12 status in subjects with and without colorectal neoplasia. Am J Clin Nutr 86:1064-1072.

71. Chen FP, Lin CC, Chen TH, Tsai MC, Huang YC (2013) Higher plasma homocysteine is associated with increased risk of developing colorectal polyps. Nutr Cancer 65:195-201.

72. Zhu X, Yi K, Hou D, Huang H, Jiang X, Shi X, Xing C (2020) Clinicopathological Analysis and Prognostic Assessment of Transcobalamin I (TCN1) in Patients with Colorectal Tumors. Med Sci Monit 26:e923828.

73. Lo-Bisgaard T, Espelund U, Frystyk J, Rasmussen TR, Nexo E, Arendt JFH (2020) Vitamin B12 and its binding proteins in patients with non-small cell lung cancer referred to fast-track diagnostic work-up for lung cancer. Scand J Clin Lab Invest 80:14-19.

74. Fanidi A, Carreras-Torres R, Larose TL, Yuan JM, Stevens VL, Weinstein SJ, Albanes D, Prentice R, Pettinger M, Cai Q et al. (2019) Is high vitamin B12 status a cause of lung cancer? Int J Cancer 145:1499-1503.

75. Tastekin D, Erturk K, Bozbey HU, Olmuscelik O, Kiziltan H, Tuna S, Tas F (2015) Plasma homocysteine, folate and vitamin B12 levels in patients with lung cancer. Exp Oncol 37:218-222.

76. Brasky TM, Ray RM, Navarro SL, Schenk JM, Newton AM, Neuhouser ML (2020) Supplemental one-carbon metabolism related B vitamins and lung cancer risk in the Women's Health Initiative. Int J Cancer 147:1374-1384.

77. Luu HN, Wang R, Jin A, Koh WP, Yuan JM (2021) The association between dietary vitamin B12 and lung cancer risk: findings from a prospective cohort study. Eur J Cancer Prev 30:275-281.

78. Bo Y, Xu H, Zhang H, Zhang J, Wan Z, Zhao X, Yu Z (2022) Intakes of Folate, Vitamin B6, and Vitamin B12 in Relation to All-Cause and Cause-Specific Mortality: A National Population-Based Cohort. Nutrients 14.

79. Kharb S, Kumar S, Kundu ZS (2015) Homocysteine, a biomarker of osteosarcoma. J Cancer Res Ther 11:51-53.

80. Gorgulu O, Selcuk T, Ozdemir S, Sayar C, Beyazit Y, Akbas Y (2010) Evaluation of the roles of serum vitamin B(12), folate and homocysteine levels in laryngeal squamous cell carcinoma. J Int Med Res 38:2047-2052.

81. Almadori G, Bussu F, Galli J, Cadoni G, Zappacosta B, Persichilli S, Minucci A, Giardina B, Maurizi M (2005) Serum levels of folate, homocysteine, and vitamin B12 in head and neck squamous cell carcinoma and in laryngeal leukoplakia. Cancer 103:284-292.

82. Nacci A, Dallan I, Bruschini L, Traino AC, Panicucci E, Bruschini P, Mancini V, Rognini F, Fattori B (2008) Plasma homocysteine, folate, and vitamin B12 levels in patients with laryngeal cancer. Arch Otolaryngol Head Neck Surg 134:1328-1333.

83. Wu YH, Wu YC, Chu FY, Cheng SJ, Sun A, Chen HM (2019) Significantly higher frequencies of hematinic deficiencies and hyperhomocysteinemia in oral precancer patients. J Formos Med Assoc 118:1299-1307.

84. Oh HK, Lee JY, Eo WK, Yoon SW, Han SN (2018) Elevated Serum Vitamin B12 Levels as a Prognostic Factor for Survival Time in Metastatic Cancer Patients: A Retrospective Study. Nutr Cancer 70:37-44.

85. Ryg J, Nybo M, Hallas J (2013) Cancer incidence in persons with elevated cobalamin levels. Eur J Clin Invest 43:557-561.

86. Maritsi DN, Vartzelis G, Dana H, Kosmidis H, Douna V, Kossiva L (2016) Plasma Folate, Vitamin B12 and Homocysteine Levels in Children with Solid Tumors at Diagnosis; Results from a Pediatric Referral Centre. Indian J Pediatr 83:483-484.

87. Ilhan IE, Sari N, Yesil S, Eren T, Tacyildiz N (2015) Anthropometric and Biochemical Assessment of Nutritional Status in Pediatric Cancer Patients. Pediatr Hematol Oncol 32:415-422.

88. Sadananda Adiga MN, Chandy S, Ramaswamy G, Appaji L, Krishnamoorthy L (2008) Homocysteine, vitamin B12 and folate status in pediatric acute lymphoblastic leukemia. Indian J Pediatr 75:235-238.

89. Urbanski G, Hamel JF, Prouveur B, Annweiler C, Ghali A, Cassereau J, Lozac'h P, Lavigne C, Lacombe V (2020) Strength of the Association of Elevated Vitamin B12 and Solid Cancers: An Adjusted Case-Control Study. J Clin Med 9.

90. Arendt JFH, Sorensen HT, Horsfall LJ, Petersen I (2019) Elevated Vitamin B12 Levels and Cancer Risk in UK Primary Care: A THIN Database Cohort Study. Cancer Epidemiol Biomarkers Prev 28:814-821.

91. Wolffenbuttel BHR, Heiner-Fokkema MR, Green R, Gans ROB (2020) Relationship between serum B12 concentrations and mortality: experience in NHANES. BMC Med 18:307.

92. Djurovic Z, Jovanovic V, Obrenovic R, Djurovic B, Soldatovic I, Vranic A, Jakovljevic V, Djuric D, Zivkovic V (2020) The importance of the blood levels of homocysteine, folate and vitamin B12 in patients with primary malignant brain tumors. J BUON 25:2600-2607.

93. Arendt JF, Pedersen L, Nexo E, Sorensen HT (2013) Elevated plasma vitamin B12 levels as a marker for cancer: a population-based cohort study. J Natl Cancer Inst 105:1799-1805.

94. Zhang D, Wen X, Wu W, Guo Y, Cui W (2015) Elevated homocysteine level and folate deficiency associated with increased overall risk of carcinogenesis: meta-analysis of 83 case-control studies involving 35,758 individuals. PLoS ONE 10:e0123423.

95. Semmler A, Simon M, Moskau S, Linnebank M (2006) The methionine synthase polymorphism c.2756A>G alters susceptibility to glioblastoma multiforme. Cancer Epidemiol Biomarkers Prev 15:2314-2316.

96. Kurzwelly D, Knop S, Guenther M, Loeffler J, Korfel A, Thiel E, Hebart H, Simon M, Weller M, Linnebank M et al. (2010) Genetic variants of folate and methionine metabolism and PCNSL incidence in a German patient population. J Neurooncol 100:187-192.

97. Zhang W, Zhang Z, Wu H, Xu K, Yuan W, Mi YY, Shi L, Zuo L, Shi YF (2020) Update analysis on the association between Methionine synthase rs1805087 A/G variant and risk of prostate cancer. Sci Rep 10:13384.

98. Gohari M, Moghimi M, Aarafi H, Shajari A, Jafari-Nedooshan J, Lookzadeh MH, Mirjalili SR, Neamatzadeh H (2021) Association of MTHFR 677C > T, 1298A > C and MTR 2756A > G Polymorphisms with Susceptibility to Childhood Retinoblastoma: A Systematic Review and Met-Analysis. Fetal Pediatr Pathol 40:612-625.

99. Bai Y, Drokow EK, Waqas Ahmed HA, Song J, Akpabla GS, Kumah MA, Agyekum EB, Neku EA, Sun K (2020) The relationship between methionine synthase rs1805087 polymorphism and hematological cancers risk. Future Oncol 16:2219-2233.

100. Ma LM, Yang HP, Yang XW, Ruan LH (2019) Methionine synthase A2756G polymorphism influences pediatric acute lymphoblastic leukemia risk: a meta-analysis. Biosci Rep 39.

101. Lu M, Wang F, Qiu J (2010) Methionine synthase A2756G polymorphism and breast cancer risk: a meta-analysis involving 18,953 subjects. Breast Cancer Res Treat 123:213-217.

102. Yu K, Zhang J, Zhang J, Dou C, Gu S, Xie Y, Mao Y, Ji C (2010) Methionine synthase A2756G polymorphism and cancer risk: a meta-analysis. Eur J Hum Genet 18:370-378.

103. Zhong S, Xu J, Li W, Chen Z, Ma T, Zhao J (2013) Methionine synthase A2756G polymorphism and breast cancer risk: an up-to-date meta-analysis. Gene 527:510-515.

104. Guo Y, Lu Y, Jin H (2020) Appraising the role of circulating concentrations of micro-nutrients in epithelial ovarian cancer risk: A Mendelian randomization analysis. Sci Rep 10:7356.

105. Semmler A, Simon M, Moskau S, Linnebank M (2008) Polymorphisms of methionine metabolism and susceptibility to meningioma formation: laboratory investigation. J Neurosurg 108:999-1004.

106. Tsilidis KK, Papadimitriou N, Dimou N, Gill D, Lewis SJ, Martin RM, Murphy N, Markozannes G, Zuber V, Cross AJ et al. (2021) Genetically predicted circulating concentrations of micronutrients and risk of colorectal cancer among individuals of European descent: a Mendelian randomization study. Am J Clin Nutr 113:1490-1502.

107. Yuan S, Carter P, Vithayathil M, Kar S, Mason AM, Burgess S, Larsson SC (2021) Genetically predicted circulating B vitamins in relation to digestive system cancers. Br J Cancer 124:1997-2003.

108. Wang X, Xiong M, Pan B, Cho WCS, Zhou J, Wang S, He B (2022) Association Between SNPs in the One-Carbon Metabolism Pathway and the Risk of Female Breast Cancer in a Chinese Population. Pharmgenomics Pers Med 15:9-16.

109. Oliai AS, Kiefte-de Jong JC, van Dijk SC, Swart KMA, van Laarhoven HW, van Schoor NM, de Groot LCPG, Lemmens V, Stricker BH, Uitterlinden AG et al. (2019) Folic Acid and Vitamin B12 Supplementation and the Risk of Cancer: Long-term Follow-up of the B Vitamins for the Prevention of Osteoporotic Fractures (B-PROOF) Trial. Cancer Epidemiol Biomarkers Prev 28:275-282.

110. Zhang SM, Cook NR, Albert CM, Gaziano JM, Buring JE, Manson JE (2008) Effect of combined folic acid, vitamin B6, and vitamin B12 on cancer risk in women: a randomized trial. JAMA 300:2012-2021.

111. Hankey GJ, Eikelboom JW, Yi Q, Lees KR, Chen C, Xavier D, Navarro JC, Ranawaka UK, Uddin W, Ricci S et al. (2012) Treatment With B Vitamins and Incidence of Cancer in Patients With Previous Stroke or Transient Ischemic Attack: Results of a Randomized Placebo-Controlled Trial. Stroke 43:1572-1577.

112. Song Y, Manson JE, Lee IM, Cook NR, Paul L, Selhub J, Giovannucci E, Zhang SM (2012) Effect of combined folic acid, vitamin B(6), and vitamin B(12) on colorectal adenoma. J Natl Cancer Inst 104:1562-1575.

113. Ebbing M, Bonaa KH, Nygard O, Arnesen E, Ueland PM, Nordrehaug JE, Rasmussen K, Njolstad I, Refsum H, Nilsen DW et al. (2009) Cancer incidence and mortality after treatment with folic acid and vitamin B12. JAMA 302:2119-2126.

114. Clarke R, Halsey J, Lewington S, Lonn E, Armitage J, Manson JE, Bonaa KH, Spence JD, Nygard O, Jamison R et al. (2010) Effects of lowering homocysteine levels with B vitamins on cardiovascular disease, cancer, and cause-specific mortality: Meta-analysis of 8 randomized trials involving 37 485 individuals. Arch Intern Med 170:1622-1631.
